# Experiences and service-uptake factors of receiving and providing care for meningitis and its sequelae: a qualitative systematic review

**DOI:** 10.1186/s12916-026-04765-4

**Published:** 2026-04-13

**Authors:** Margarita Andreeva, Maria Pyatnitskaya, Karina Kochneva, Ekaterina Sviatskaia, Maria Spryshkova, Melissa Meyer, Dina Baimukhambetova, Mark Kosenko, Aigun Mursalova, Nina Avdeenko, Elena Kondrikova, Jonathan Zheng, Ka Yan Cheung, Audrey Dunn Galvin, Tom Jewell, Zoe Moula, Sarah Neill, Mikhail Zinchuk, Alla Guekht, Daniel Munblit

**Affiliations:** 1 University of British Columbia, Vancouver, Canada; 2 Erasmus School of Health Policy & Management, Rotterdam, Netherlands; 3 Moscow Research and Clinical Center for Neuropsychiatry, Moscow, Russia; 4 University College Cork, Cork, Ireland; 5 Department of Paediatrics and Paediatric Infectious Diseases, Institute of Child’s Health, I.M. Sechenov First Moscow State Medical University, Sechenov University, Moscow, Russia; 6 Department of Electrical and Electronic Engineering, Imperial College London, London, UK; 7 Faculty of Medicine, Imperial College London, London, UK; 8 Care for Long Term Conditions Division, Florence Nightingale Faculty of Nursing, Midwifery and Palliative Care, King’s College London, London, UK; 9 University of Plymouth, Plymouth, UK

**Keywords:** Barriers to care, Care provision, Facilitators of care, Healthcare provider experiences, Meningitis, Meningitis sequelae, Patient experiences, Qualitative evidence, Service uptake, Systematic review

## Abstract

**Background:**

Meningitis is a life-threatening condition with substantial morbidity and mortality, especially in low- and middle-income countries (LMICs). While quantitative research has enhanced the understanding of diagnostic and treatment efficacy, qualitative data regarding the lived experiences remain underexplored. This systematic review synthesises qualitative evidence on the experiences of patients, caregivers, and healthcare providers regarding meningitis diagnosis, treatment, and care, as well as the barriers and facilitators influencing service uptake and provision. The review aimed to inform the development of World Health Organization guidelines for improving meningitis care.

**Methods:**

This systematic review was registered in PROSPERO (CRD42024514413). A comprehensive search of EMBASE, MEDLINE, PsycINFO, and CINAHL was conducted on February 13–14, 2024. Studies were included if they explored patient, caregiver, or healthcare provider experiences with meningitis or sequelae diagnosis, treatment, or long-term management. Thematic synthesis was used for data analysis. Quality assessment of each included study was performed using the Critical Appraisal Skills Programme checklist. Confidence of evidence was assessed using the GRADE-CERQual approach.

**Results:**

Out of 4735 identified studies, 19 were included in the final synthesis; seven were conducted in LMICs and 12 in high-income countries (HICs). The synthesis identified four overarching analytical constructs: diagnostic uncertainty and disparities in initial help-seeking; systemic and operational constraints on acute care delivery; the relational and psychological burden of meningitis; and barriers to long-term equity and continuity of care. Key themes included diagnostic challenges, delays in care-seeking due to cultural and socioeconomic barriers, and miscommunication between patients and healthcare providers. Critical barriers to care included systemic healthcare limitations, financial constraints, and inadequate public awareness of meningitis symptoms. Limited data on facilitators of timely care uptake were found.

**Conclusions:**

This synthesis highlights substantial context-specific disparities in meningitis care. In LMICs, the primary points of failure relate to access and resources, with financial barriers, cultural reliance on traditional healing, and systemic operational limitations impeding timely diagnosis and treatment. In HICs, the challenges are rooted in clinical processes and support systems, particularly healthcare workers’ diagnostic uncertainty, failures in patient-provider communication, and the absence of psychological support. Addressing these barriers through culturally sensitive interventions, improved healthcare infrastructure, enhanced patient-provider communication, and public health education is essential.

**Supplementary Information:**

The online version contains supplementary material available at 10.1186/s12916-026-04765-4.

## Background

Meningitis is a serious, life-threatening condition characterised by the inflammation of the meninges, the protective membranes covering the brain and spinal cord. It can be caused by a variety of infections, including bacterial, viral, fungal, or parasitic, with bacterial meningitis considered the most severe and often leading to significant morbidity and mortality [1]. Despite advances in medical science with high-quality diagnostic tools and treatment modalities in place, meningitis remains a global health challenge, particularly in low- and middle-income countries (LMICs), where access to healthcare and preventive measures can be limited [2].

Existing literature predominantly focuses on the analysis of quantitative evidence, such as the effectiveness of specific diagnostic tools and interventions [3–5]. Qualitative evidence, which encompasses the values, beliefs, and experiences of patients, caregivers, and healthcare providers, remains sparse. To date, no systematic review has synthesised this qualitative evidence, which is essential for a holistic understanding of the impact of meningitis and its sequelae on all relevant stakeholders; planning appropriate response from the public health and policy making perspective; and for the development of care strategies.

The experiences of individuals who have undergone diagnosis, treatment, and care for meningitis are critical in understanding potential barriers to care as well as the broader impact of the disease. Patients and their families often face substantial physical, emotional, and financial burdens. These experiences are varied and influenced by numerous factors, including the severity of the disease, the timeliness of diagnosis and treatment, and the support systems available. The sequelae of meningitis, such as neurological symptoms, hearing loss, and/or cognitive impairments, may have long-lasting effects on quality of life [6]. It is also important to consider healthcare provider experiences and perspectives as they are essential in identifying the challenges and barriers within health systems that affect the provision of care. Understanding these experiences can inform strategies to improve service delivery, enhance patient outcomes, and support healthcare workers in their roles. There may be important differences in experiences between high-income countries (HICs) and low- and middle-income countries (LMICs) which may inform policies development/interventions tailored to specific populations and settings [7].

This systematic review aimed to comprehensively assess qualitative evidence on the experiences of end-users, barriers and facilitators to healthcare delivery of diagnosis and management of meningitis and its sequelae, to inform the scope of new World Health Organization (WHO) guidelines on meningitis care. This qualitative systematic review was undertaken to identify the values and experiences of individuals regarding the diagnosis, treatment, and care for meningitis, as well as the barriers and facilitators influencing the uptake and provision of these services.

## Methods

This systematic review employs a qualitative evidence synthesis to explore the experiences of end-users and healthcare providers concerning the diagnosis, treatment, and care for meningitis, and factors influencing uptake or provision of these services. The review followed the Preferred Reporting Items for Systematic Reviews and Meta-Analyses (PRISMA) guidelines. The review protocol was registered with the National Institute for Health Research’s PROSPERO (PROSPERO 2024 CRD42024514413 available from https://www.crd.york.ac.uk/prospero/display_record.php?ID=CRD42024514413) on February 22, 2024.

### Search and screening

We conducted a comprehensive electronic search at EMBASE on February 13, 2024, MEDLINE, PsycINFO, and CINAHL databases on February 14, 2024. The search strategy incorporated a combination of free-text words and Medical Subject Headings (MeSH) terms related to meningitis, qualitative research, and healthcare experiences. The search strategies are provided in Additional file 1: Box 1. At the screening phase, further studies were traced through reference lists of included studies to ensure that no original published data had been missed. Authors of identified papers and experts in the field were contacted for missing data when appropriate.

The identified items were imported into Covidence (Veritas Health Innovation, Melbourne, Australia). Pairs of authors (MA, MP, KK, ES, MS, DB, MK, and AM) independently conducted the title and abstract screening, followed by full-text screening. Any disagreements between the screeners were resolved via consensus or a third reviewer (MA, MP, and/or DM).

### Eligibility criteria and selection of papers

This project has been designed to meet the needs of the WHO Guideline Development Group on Meningitis, and eligibility criteria were aligned to those used in the guideline development process.

#### Types of studies

Any studies that employed qualitative methods of data collection, such as individual interviews, focus group discussions, qualitative surveys and questionnaires, observations, and diaries, along with qualitative data analysis methods, including thematic analysis, grounded theory, framework analysis, and thematic network analysis. Mixed-methods studies with qualitative component available were also considered.

Studies were excluded if they reported on cryptococcal, tuberculous, hospital-acquired, or non-infectious meningitis; involved participants younger than 1 month; or were books, abstracts, case reports, conference papers, theses, reviews, and manuscripts without full text available. Only studies published in English were considered for inclusion in this systematic review.

#### Types of participants

End-users (adults, adolescents, and children older than 1 month) defined as persons who have undergone diagnosis, treatment, or care for meningitis. Caregivers and family members of people with community-acquired meningitis and health and care workers delivering care for meningitis.

#### Types of interventions/exposures

We included the studies, which were collecting qualitative evidence looking at values and experiences related to the main outcomes of diagnosis, treatment, and care; views of end-users, caregivers, and healthcare professionals on acceptability of the services; feasibility, accessibility, and affordability of the services; and on how would implementation of interventions for diagnosis, treatment, and care impact health equity, equality, and non-discrimination.

#### Outcomes of interest

Views and experiences of patients, carers, and healthcare providers of engagement with services in diagnosis, treatment, and care of acute, community-acquired meningitis and its sequelae as well as factors influencing the uptake and/or provision of the diagnosis, treatment, and care services.

### Data extraction and thematic analysis

All relevant data from the articles included in the study were extracted into Microsoft Excel spreadsheets. Data were extracted on the first author, year of publication, country, and method of data collection and analysis. Additionally, information was gathered on study characteristics, including number of participants, demographics, and the aetiology of meningitis.

A thematic synthesis approach allowed for flexible and rigorous integration and synthesis of individuals’ views and experiences. The process has been conducted in Microsoft Excel as a three-stage process: (1) line-by-line coding; (2) generation of descriptive themes; and (3) development of analytical themes, supported by codes and descriptive themes, and then applied to the WHO evidence-to-decision framework.

For each study, the ‘results’ or ‘findings’ sections, including both author narratives and participant quotations, were coded by five team members (MA, MP, ES, MS, and KK) independently to promote reflexivity and enhance rigour. An inductive and iterative approach to analysis was prioritised throughout, allowing significant themes, topics, or models identified directly from the raw data. In the final stage of synthesis, a deductive phase was introduced to address the review question. Rigour and reflexivity were reinforced through critical dialogue between reviewers. A reflexivity statement is provided in Additional file 1: Box 2.

Separate themes were developed for the (a) ‘acute phase of meningitis’ (stratified into pre-hospitalisation and hospitalisation period) and (b) ‘meningitis sequelae’. ‘Experiences and values’, ‘barriers to care delivery’, and ‘facilitators of service uptake’ were considered (Additional file 1).

### Risk of bias and certainty of evidence assessment

We performed a quality assessment of each study using the Critical Appraisal Skills Programme (CASP) checklist for qualitative studies. Any discrepancies in the quality assessment were addressed through team discussion. Studies were assessed, but not excluded based on the outcome of their quality assessment.

The GRADE-CERQual approach (Confidence in the Evidence from Reviews of Qualitative Research, https://www.cerqual.org/) was used for the evaluation of the confidence of evidence presented in the review findings across four domains: methodological limitations, coherence, adequacy of data, and relevance, and was informed by the quality assessment of the included papers.

#### Role of the funding source

The funder of the study, the World Health Organization, was involved in the discussion of the inclusion criteria, but had no role in the study design, data collection, data analysis, and/or data interpretation.

## Results

**Fig. 1:**
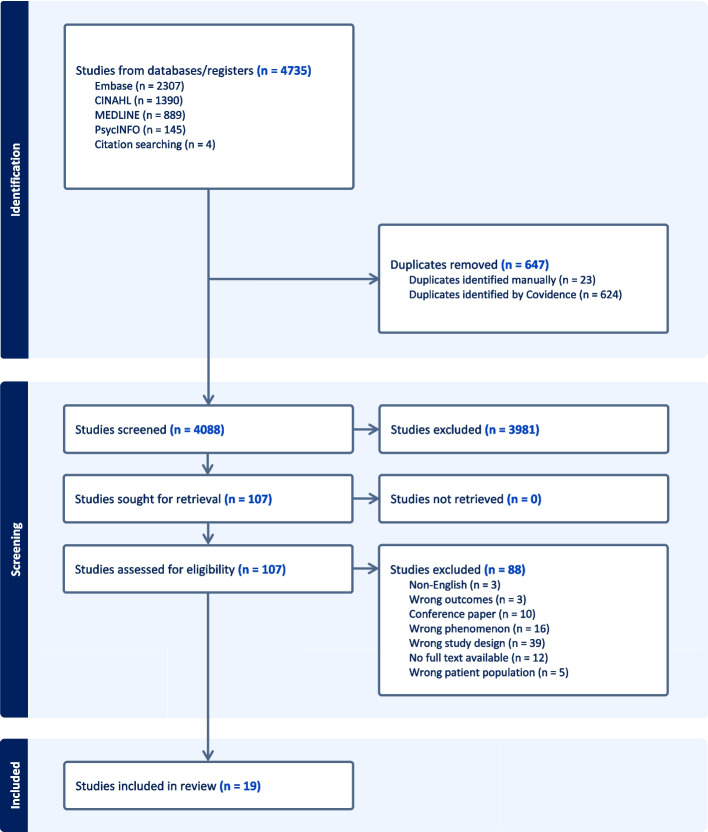
PRISMA flowchart

The initial searches found 4735 publications; after removing duplicates (*n* = 647), 4088 records were identified, and titles and abstracts were screened. Of these, 107 met the inclusion criteria and were eligible for full-text assessment. Eighty-eight studies were further excluded, and 4 additional publications were identified through reference list screening (Fig. 1), with 19 papers included in this review [8–26].

### Study characteristics

Studies were conducted in a range of countries (Table 1), with seven in LMICs: Nigeria (*n* = 3), Burkina Faso (*n* = 1), Malawi (*n* = 1), Senegal (*n* = 1), and Zambia (*n* = 1). Twelve studies were undertaken in HICs: the UK (*n* = 5), USA (*n* = 2), France (*n* = 2), and Australia (*n* = 2), with a single study conducted in the UK and Ireland.

**Table 1:** Characteristics of included studies

First author, year	Country	Participant type	Method of data collection	Time between meningitis episode and interview	Method of data analysis	Sample size, age, sex	Participant characteristics	Themes suggested by the author
Low- and middle-income countries								
Adedini et al, 2021 [9]	Nigeria	C, HCW	Focus group discussions and in-depth interviews	NR	Thematic analysis	*N* = 259 (84 interviews: 60 in-depth interviews, 24 focus group discussions) Age: 18–34 years Female = 86.1%	Pregnant women/nursing mothers, older women, men, traditional birth attendants/traditional medicine practitioners, faith-based healers, skilled healthcare providers, religious and community leaders Ethnicity: Hausa/Fulani, Igbo, Yoruba	• Respondents’ perception and knowledge about the causes of pneumonia, diarrhoea, malaria, and meningitis • Respondents’ perception and knowledge about prevention of PDMM • Respondents’ perception and knowledge of symptoms and fatality of PDMM • Caregivers’ preventive practices and managements of childhood pneumonia, diarrhoea, malaria, and meningitis
Colombini et al, 2009 [20]	Burkina Faso	P, C, Com	Interviews with structured standardised questionnaires and focus group discussions	NR	Qualitative data analysis (specific method not reported)	People with lived experience *N* = 116; community members *N* = 165 Age: all ages	People with lived experience, carers, community members (including religious, administrative, and traditional community leaders) Ethnicity: Lyélé, Moaga	• Social constructions of meningitis • Preventive and therapeutic practices
Desmond et al, 2013 [11]	Malawi	P, C, HCW, Com	Narrative in-depth interviews (P, C), semi-structured interviews (with HCWs), and focus group discussions (Com)	NR	Thematic content analysis using an initial coding framework that further guided the development of a treatment-seeking framework	*N* = 133 P/C *N* = 17; Com *N* = 96; HCWs *N* = 20 Age: adults—median 28 years (range 18 to 48); children < 5 years	Patients identified as adult and paediatric hospital in-patients, carers, community (established community-based social groups such as women’s or microfinance groups), HCWs (private and government primary and community health service workers)	• Recognition of meningitis as dangerous • Recognition of severe illness • Delays in treatment seeking • Following recognition of severe illness • Need to validate severity • Household economics • Perceptions of service quality
Elafros et al, 2022 [21]	Zambia	P, C, HCW	Semi-structured interviews	NR	Modified thematic approach	*N* = 173 (24 adult patients, 36 carers of adult patients, 63 carers of paediatric patients, 20 doctors, and 30 nurses) Age: ‘most were aged 24–48 years’ Sex: female 66%	Included participant groups: patients with a suspected CNS infection (meningitis), caregivers, doctors, nurses	LP barriers: • Community apprehensions • Proxy family consensus for LP consent • Competing clinical demands LP enablers: • Perceived utility of LP • Perception of HCW comfort with LP • In-person counselling
Griffiths et al, 2012 [19]	Senegal	C	Interviews using detailed sociodemographic and cost questionnaires	Median 5 years 5 months (IQR: 54–75 months)	Qualitative—NR Quantitative: cost data analysis	*N* = 66 Age at follow-up: average = 6 years and 8 months (4–10 years)	Included participant groups: caregivers of children with meningitis. Family members interviewed: mother—62%; grandmother—17%; father—12%; grandfather—5%; other caregivers—5% Ethnicity: 9 different ethnicities; nearly half of the children—Wolof	• None (Separate quotes on financial burden of treatment costs, unaffordable treatment costs, and productivity costs of caregivers’ time)
Mahmoud et al, 2022 [17]	Nigeria	P, C, Com	In-depth interviews (using multi-stage sampling technique and an informant-based survey; Triangulation techniques in the form of interviewing medical personnel)	NR	Thematic framework approach	*N* = 43 (20 meningococcal meningitis patients, their carers (20), and 3 traditional healers) Age (patients): average age 72.4 (SD: 1.25) Age (caregivers): 25–49 years Sex (carers): female 50%	Included participant groups: patients (older patients), caregivers (daughters, sons, wives, spouses, ex-wives, other family members), traditional healers	• Causes and symptoms of meningitis • Traditional treatment and management methods of meningitis • Family caregivers for older patients suffering from meningitis
Omoleke et al, 2018 [13]	Nigeria	C, HCW, Com	In-depth interview, focus group discussions	NR	Thematic framework approach; content analysis method	*N* = 40 Age: 25–70 years	Carers, HCWs (health practitioners from the communities and health facilities in the communities), community members (household heads, Quranic School Mallams)	• Environmental factor as a driver or risk factor for CSM transmission • Economic factors • Sociocultural factors
High-income countries								
Brennan et al, 2003 [8]	UK	HCW	Semi-structured interviews	NR	Grounded theory	*N* = 26	General practitioners	• Fear about meningitis and septicaemia • Reaching a diagnosis of meningitis or septicaemia • Treating suspected cases and the value of guidelines
Clark et al, 2013 [10]	UK, Ireland	C	Multiple choice questionnaire and semi-structured interviews	Median 5 years	Grounded theory (constant comparison method)	*N* included = 194. *N* interviewed = 18; age of children at the time of illness: mean 3 years 10 months	Parent/legal guardian of children who had survived meningitis or septicaemia Only those parents reporting permanent after-effects, and who had accessed aftercare and support, were invited to the interview	• Accessing appropriate support and follow-up care • Communication
Erickson et al, 2001 [26]	USA	P	Telephone interviews	NR	None	Number of interviewed participants included in the qualitative part of the study *N* = 17	Patients (college students)	None
Granier et al, 1998 [12]	UK	HCW	Semi-structured interviews	Mean 61 (SD 39) weeks	Qualitative data analysis (specific method not reported)	*N* (HCW) = 26; number of included cases of IMD (general practice sample) *n* = 31 Age: mean 42 years (range 29–55 years) Sex (HCW): female 38%	General practitioners who referred children and adolescents under 16 years old with IMD to hospitals	• Rashes • Abnormal illness • Puzzling findings • Misleading information • Role of parents • Management in primary care
Haines et al, 2005 [14]	UK	C	Focused interviews	1 month following discharge from the hospital	Heideggerian phenomenological approach Colaizzi’s interpretation process	*N* = 7	Parents of childhood IMD survivors admitted to PICU	• Complications/side effects of the disease • Emotional turmoil • Child’s physical appearance • Family disruption • Fear of death • Loss of parenting role • Need for support and understanding • Need and value of communication/information/publicity • Parental intuition • Technological interventions • The impact of care delivery
Jarvinen et al, 2005 [25]	Australia	HCW	Structured phone interviews (based on the PRECEDE-PROCEED model), free discussion was encouraged	NR	None (some qualitative data was included)	Total *N* = 29 Interviewed GPs *N* = 20 Sex: female 25%	GPs	None
Kupst et al, 1983 [24]	USA	C	Semi-structured interviews including follow-up interviews	NR (other assessments were performed 1–2 years post-diagnosis)	Quantitative analysis of semi-structured interviews (qualitative data)	*N* total = 28 families	Parents of childhood bacterial meningitis survivors	None
Neill et al, 2022 [22]	UK	C	In-depth interviews, focus groups (parents of meningitis patients were interviewed only in focus groups—stage II of the study)	NR (stage II data were collected for participants who have had experience of meningitis between 2011 and 2018)	An explanatory modified grounded theory analysis; Glaser’s 6 Cs coding frame (5 Cs were applied in the study: context, conditions/antecedents, causes, contingencies/influencing variables, and consequences)	*N* = 18 Age (caregivers): 30–50 years (30–39 years—11/18 (61%); 40–49 years—5/18 (28%)) Sex: female 78% Families with experience of meningitis—14/16 (88%; based on the *N* of families engaged in stage II (*N* = 16))	Included participant groups: caregivers of children with a serious infectious disease (mothers *n* = 15, fathers *n* = 2) Ethnicity: White British 67%, White other 17%	• Navigating uncertain illness trajectories • The family and the health services: context • Social expectations and social hierarchies: the antecedents • Influencing variables or contingencies • Consequences
Scanferla et al, 2020 [23]	France	P	Semi-structured interviews	Average 8.9 years (SD = 8.2)	Interpretative phenomenological analysis	Number of participants: *N* total = 9 Sex: female 78% Age at follow-up: 18–48 years old (mean = 28.3, SD = 11.4)	People with lived experience of meningitis	• Meningitis disease (non-spontaneous theme) • Repercussions of the meningitis experience • Memory, memories • Knowledge/ignorance • Temporality • Emotions • Relationships • Healthcare and professionals
Scanferla et al, 2021 [15]	France	C	In-depth semi-structured interviews	Average 9.39 (SD = 5.4) years	Interpretative phenomenological analysis	*N* = 11 Age of survivors: 3–30 years old (mean = 13.45, SD = 9.37) at the time of the interviews Sex of survivors: female—73%	Carers of childhood meningitis survivors (10 mothers and 1 grandmother)	• Meningitis disease • Healthcare services and professionals • Knowledge/ignorance • Repercussions of the meningitis experience: ‘life afterwards’ • Sick child attitudes/behaviour • Sibling attitudes/behaviour
Sweeney, 2013 [16]	UK	C	Structured interviews (three open-ended questions)	NR	Qualitative content analysis of free-text responses	*N* = 244	Carers of childhood meningitis survivors	• Information provision • Symptom awareness • Medical follow-up care • Recognition of and provision for additional needs • Impact of the disease • Reassurance
Wisemantel, 2018 [18]	Australia	C	Review of medical records and semi-structured interviews with parents; structured interview of a key informant (social worker)	5–6 years	Thematic analysis with inductive and deductive techniques	*N* = 6	Carers of childhood IMD survivors	• Unclear about IMD • Support needs • Emotional turbulence • Personal growth

*P*, patients; *C*, carers; *CNS*, central nervous system; *Com*, community; *CSM*, cerebrospinal meningitis; *HCW*, healthcare workers; *LMICs*, low- and middle-income countries; *HICs*, high-income countries; *IMD*, invasive meningococcal disease; *IQR*, interquartile range; *N*, number; *NR*, not reported; *PICU*, paediatric intensive care unit; *SD*, standard deviation

The sample sizes ranged from 6 to 281 individual participants or groups of participants. The types of participants varied, with healthcare providers being included in 7 studies, patients in 6 studies, and caregivers in 14 studies. Some studies involved patients, caregivers, and healthcare workers (*n* = 2), while others included specific groups such as meningitis survivors, religious/traditional leaders, and community members (*n* = 4). Participant age varied widely across the included studies. Paediatric patients ranged from infants to young adults aged 30 years at the time of interview. Among studies enrolling adult patients, ages spanned university students to older adults. Carers were between 18 and 70 years old and provided support for both paediatric and adult patients. Several studies that recruited parents of child survivors, however, did not specify carers’ ages (Table 1).

A variety of data collection methods were used across the studies. Semi-structured interviews were the most commonly used method (*n* = 8), followed by in-depth interviews (*n* = 4). Focus group discussions were held in three studies, while mixed methods were employed in a single study. Other methods included structured interviews (*n* = 2), narrative interviews combined with other approaches (*n* = 1), and telephone interviews (*n* = 2). Two studies involved triangulation techniques or used detailed sociodemographic and cost questionnaires in addition to interviews.

All studies were evaluated as per the CASP checklist assessment (Additional file 1: Table S1). The most common concern was related to uncertainty in adequacy of the relationship between researcher and participants, which was present in 13 (68%) studies. In some studies, the research design was not entirely appropriate to address the aims of the research or unclear (7 (37%) studies). The GRADE-CERQual assessment of confidence of review findings is available in Additional file 1: Table S2.

#### Final themes

**Fig. 2:**
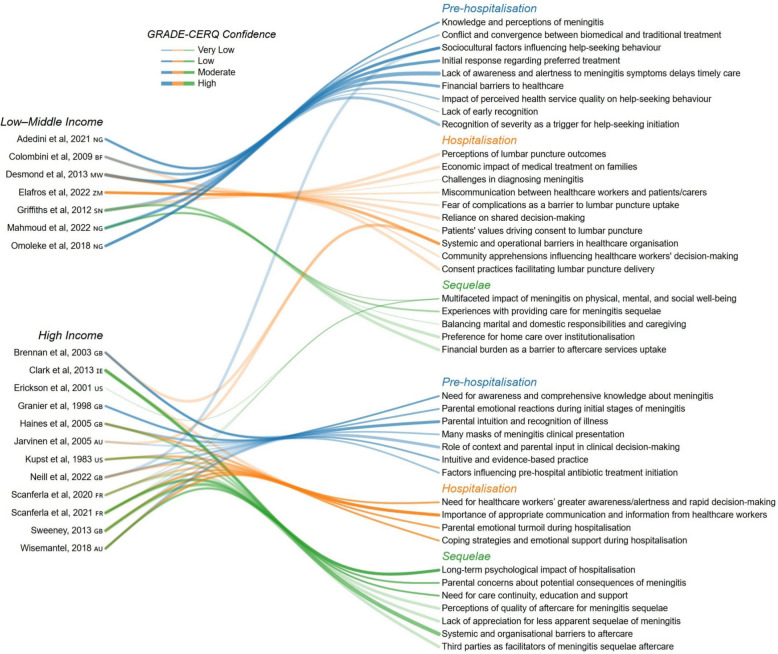
Identified themes and associated GRADE-CERQual assessment

We developed 45 themes (18 related to pre-hospitalisation, 14 to hospitalisation, and 13 to post-hospitalisation/sequelae), defined in Fig. 2 and Additional file 1: Table S3.

To enhance narrative coherence and support policy translation, the detailed findings have been synthesised into four overarching analytical constructs, which delineate the principal challenges across the care pathway and within different contexts: (A) diagnostic uncertainty and disparities in initial help-seeking; (B) systemic and operational constraints on acute care delivery; (C) the relational and psychological burden of meningitis; and (D) barriers to long-term equity and continuity of care.

### Acute phase of meningitis

#### Pre-hospitalisation period

##### Healthcare workers

Evidence regarding experiences and values of healthcare workers (HCWs) were available from HICs only, with three themes identified.

### Experiences and values

#### Many masks of meningitis clinical presentation

Meningitis presents a range of symptoms, making it difficult for HCWs to recognise it, especially when the clinical presentation does not follow classic ‘textbook cases’. The usual meningitis symptom of a purpuric non-blanching rash was often considered a defining feature of the disease that prompted general practitioners (GPs) to admit patients to the hospital. However, many cases are less clear-cut, and GPs sometimes struggle with patients who exhibit vague or non-specific symptoms. One GP recounted how a patient’s symptoms initially seemed unrelated to meningitis but raised concern due to a characteristic sign and the speed of deterioration:

*I really didn’t have a clue what was wrong with him... The only thing that made me seriously think about meningitis was the fact he’d had this ache in his neck the night before and became extremely ill quickly.* (Granier, 1998)

GPs also encountered cases where the symptoms suggested other conditions, such as Henoch-Schönlein purpura and thrombocytopenic purpura, or where the child appeared unusually well despite having a serious illness. The variety of presentations—from mild rashes to severe neurological symptoms—makes diagnosing meningitis a challenging task, especially in primary care settings where early and subtle signs can be easily missed.

#### Role of context and parental input in clinical decision-making

GPs mentioned the role of parental concerns in influencing their decisions, especially when they had an established relationship with the family. Parental emotional cues, such as visible distress and anxiety, were often used as key indicators to guide clinical action. In some cases, parents directly prompted GPs to reconsider their initial assessments.

Parental fear of meningitis often influenced consultations; many parents expressed concerns raised by awareness campaigns, leading GPs to consider meningitis as a cause of febrile illness, even if they had not initially considered that. GPs recognised that while parental fears were sometimes disproportionate, they were understandable given the potential severity of the disease. The awareness of parental fears, combined with clinical judgement, seems to play an important role in guiding GPs towards an appropriate diagnosis, even when the presenting symptoms are ambiguous.

#### Intuitive and evidence-based practice

GPs often balance evidence-based guidelines with intuitive, ‘experience-driven’ decision-making. While GPs recognised the value of evidence-based guidelines, many emphasised the importance of intuition and gut feeling in diagnosing serious illnesses like meningitis. The unpredictable nature of the disease often calls for experience-based judgements, as guidelines may not always capture the nuances of early-stage presentations:

*Experience-based practice as opposed to evidence-based practice... There is an awful lot of general practice that is based on experience. You make an awful lot of decisions just for reasons that you can’t define.* (Brennan, 2003)

In particular, GPs frequently rely on less systematic indicators, such as the overall appearance of the child or changes in behaviour, to identify serious illnesses. While guidelines provided a framework for managing cases, many GPs felt that they can ‘depersonalise’ the doctor-patient relationship and limit the role of intuition.

**Barriers and facilitators**

There is a complex interplay between logistical challenges, healthcare providers’ confidence in their expertise, and certainty in diagnosis, leading to two themes related to barriers and facilitators. The data were available from HICs only.

#### Systemic and operational barriers in healthcare organisations

Healthcare providers in HICs faced systemic and operational barriers that impact their ability to provide immediate care for children suspected of meningitis. These barriers come from lack of experience, concerns about protocol, and logistical challenges, all of which contributed to delays in timely intervention.

One of the most significant barriers reported by HCWs is a lack of experience with administering parenteral antibiotics to children, which generates considerable anxiety among practitioners. Many GPs expressed discomfort with this responsibility, preferring instead to transfer the child to secondary care where more experienced clinicians could take over.

External advice, particularly from nearby emergency departments, also played a significant role in GPs’ hesitation to initiate treatment. Concerns about overprescribing antibiotics, reinforced by messages from the National Prescribing Service, further discouraged GPs from administering antibiotics. The combination of logistical issues, concerns about proper antibiotic use, and anxiety over lack of experience represents a significant systemic and operational obstacle to effective pre-hospital intervention in suspected cases of meningitis.

#### Factors influencing pre-hospital antibiotic treatment initiation

GPs were more likely to administer antibiotics pre-hospital when they were confident in their diagnosis, particularly when classic symptoms such as a haemorrhagic rash were present. However, even in cases where the diagnosis was less certain, the presence of severe symptoms often prompted GPs to act.

The presence of a non-blanching rash was identified as one of the most reliable indicators that led to the initiation of antibiotic treatment. This symptom significantly influenced the decision-making process and was viewed as an urgent sign requiring hospital admission and initiation of antibiotics.

GPs expressed a desire for clarity in symptoms before administering antibiotics. Many were hesitant to initiate treatment in the absence of definitive signs, preferring to wait until the diagnosis was clear.

##### Patients, carers, and community members

**Experiences and values**

Five themes were identified, with two representing data from LMICs and three arising from HIC settings.

#### Knowledge and perceptions of meningitis

In LMICs, in rural communities, the causes of meningitis are frequently linked to spiritual or supernatural influences. For example, in northern Nigeria, respondents commonly believed that meningitis was an act of God or the result of spiritual forces. This perception was motivated by traditional beliefs in sorcery, Jinn, or witchcraft:

*Participants believe that some bad Jinn cause deadly diseases to people, such as meningitis, and suggest that Jinn might have possessed those suffering from the disease.* (Mahmoud, 2022)

While spiritual explanations were prevalent, some communities also attributed meningitis to environmental factors. The heat, sun, and wind, particularly the harmattan winds during epidemic seasons, were cited as causes for the disease. Dietary factors, such as food contaminated by wind during epidemic seasons, leftover food from an ill person, and undercooked or oily food, were also perceived to elevate the risk of meningitis. Eating green mangos and natural causes in general were perceived to especially affect children*.*

Knowledge about meningitis symptoms varied across communities. Seizures, stiff neck, and fever were the most commonly recognised symptoms, with stiff neck often serving as a primary indicator. Additionally, there was widespread recognition of the disease’s potential for causing serious consequences: respondents feared death and the long-term sequelae of meningitis, including hearing loss, mutism, and mental impairment.

Conflict and convergence between biomedical and traditional treatment

Despite the persistence of traditional beliefs in LMICs, many caregivers and patients expressed a preference for biomedical treatment when it came to managing meningitis.

Sometimes the sentiment that biomedicine is the most effective in diagnosing and treating meningitis coexisted with spiritual beliefs about the cause of the disease. For example, one caregiver noted:

*While I understand that there are evil-doers and Jinns, I still prefer medical treatments to traditional approaches.* (Mahmoud, 2022)

Despite the growing acceptance of biomedical care, there remains a deep-rooted conflict with traditional beliefs about the illness, which were described in the previous theme. Some patients expressed scepticism towards the medical diagnosis of meningitis, attributing their illness to curses, dreams, or old age.

#### Need for awareness and comprehensive knowledge about meningitis

The theme of knowledge of meningitis causes and symptoms was also apparent in HICs, but connotation of the theme was different. Parents reported having very little or no prior knowledge of meningitis before their child’s diagnosis. Many participants shared that they had heard of the disease but did not fully understand its symptoms or severity. As one mother explained:

*I didn’t even know what it was. I didn’t even know there were three forms of meningitis; I didn’t even know what ‘purpura fulminans’ was... I’d never even heard of it.* (Scanferla, 2021)

Once meningitis was diagnosed, many parents expressed a strong desire to gain more knowledge about the disease, often approaching the internet or support groups to fill in the gaps. Some families also shared that healthcare providers did not always provide sufficient details, prompting them to seek out this information on their own. Parents were highlighting the importance of better public education and awareness campaigns to ensure that more families could recognise the signs of meningitis early and beyond the commonly known rash.

Parents in HICs experience intense emotional reactions when their child first shows symptoms of meningitis. These emotions range from fear and frustration to helplessness, as parents try to navigate the healthcare system and manage their child’s deteriorating condition.

Parents expressed feelings of anger and disbelief when their concerns were initially dismissed by healthcare providers. Parents also described a profound sense of helplessness when they were unable to improve their child’s condition. This loss of control was a common theme, as many felt that their role as a caregiver was undermined by the severity of the illness:

*I’m the Mum, I should be able to make my child better, but I couldn’t.* (Neill, 2022)

Even when their child was transferred to the hospital by a specialist team, some parents continued to feel anxious and distressed as they were unable to accompany their child.

Parental intuition and recognition of illness

In HICs, parents’ intuition prompted them to seek medical attention, even when the symptoms did not immediately suggest meningitis. Many parents described how they knew their child was seriously ill based on changes in their behaviour or physical condition, often stemming from a deep, instinctual sense that something was wrong:

*The first thing I say to people is he never had spots... he never vomited, he was sick and he was sick to the point where I knew something wasn’t right, it wasn’t a normal virus that he had.* (Wisemantel, 2018)

In some cases, parents were able to identify worrying symptoms that healthcare professionals initially missed. They used specific language to describe changes in their child’s behaviour, which indicated that the illness had progressed from mild to serious, through phrases, such as ‘not herself’ or ‘not there behind the eyes’.

**Barriers and facilitators**

Several themes describe barriers to diagnosis and management of meningitis in pre-hospitalisation period. All themes apart from ‘sociocultural factors’ are described by study participants from LMICs.

A combination of cultural, financial, and systemic factors contributed to delay in seeking timely medical care for meningitis in LMICs.

Sociocultural factors play a critical role, impacting health-seeking behaviour regardless of the settings. These factors can either delay or lead to timely medical intervention depending on family structures, societal norms, and community expectations.

In LMICs, health-seeking behaviour was often influenced by collective decision-making within families and communities. Individual recognition of illness severity was frequently insufficient to initiate treatment seeking. Instead, decisions are made through social validation from senior family members or community leaders. Gender roles further complicate decision-making, particularly for women, who often require confirmation from male relatives before seeking medical care. This delays treatment, as women must navigate familial hierarchies:

*My brother-in-law said “you must force him” but I explained that he wouldn’t listen to me so he told him that he should go to hospital. So he my husband said “I have heard, I will go”* (Desmond, 2013)

Cultural practices and religious beliefs also shape health-seeking behaviour. In many cases, individuals prefer to seek treatment from traditional healers or religious leaders, particularly when the illness is believed to have spiritual causes. Socioeconomic factors, such as the cost of healthcare, also play a significant role in delaying medical intervention. In resource-constrained settings, families turn to traditional healers due to lower costs and shorter waiting times.

In HICs, sociocultural factors such as balancing parental responsibilities and perceptions of social norms around healthcare use influence health-seeking behaviour. Parents experience conflict between meeting daily family obligations and recognising the severity of their child’s illness. This conflict can lead to delayed medical care.

The fear of overusing medical resources or misjudging the severity of the illness can lead to further delays. Parents may hesitate to seek medical care due to uncertainty about whether the severity of child’s disease warrants the use of healthcare services. The appropriateness of using healthcare services is perceived to be dictated by social expectations and norms, and some parents feel pressured to follow them.

#### Initial response regarding preferred treatment

As already described, the prevalent belief in many LMICs communities is that diseases like meningitis are caused by spiritual influences and should be treated with traditional means. Most families use prayers and consultations with traditional healers as the first line of treatment, particularly in cases of mild symptoms. Families also turn to alternative care (e.g. traditional healers) providers due to shorter waiting times and lower costs, with hospitals being viewed as a last resort. However, some participants noted that they seek advice from traditional healers regardless of the availability of other resources, underscoring the strong influence of cultural beliefs on medical decision-making.

Self-medication is another common initial response, particularly when symptoms are perceived as minor. Families administer over-the-counter drugs like paracetamol or use herbal remedies like anointing oil to manage symptoms, seeking medical care only when the condition worsens.

#### Lack of awareness and alertness to meningitis symptoms delays timely care

The lack of awareness and understanding of meningitis symptoms often leads to delays in seeking medical care. Seizures and headaches are often mistaken for conditions prevalent in LMICs, such as malaria or epilepsy. This problem is also illustrated by the lack of meningitis recognition as a possible diagnosis in the elderly, with symptoms like weakness or loss of appetite attributed to old age.

#### Financial barriers to healthcare

Many families in LMICs delayed seeking treatment due to financial constraints. In order to cover emergency expenses, including transportation and medical costs, families may have to borrow money, which leads to further delays. Even in countries with universal healthcare, the indirect costs such as transportation remain prohibitive for many families:

*The wife delayed because of borrowing money, and the child died on the way.* (Desmond, 2013)

Lack of funds also influences the type of treatment families pursue, with many initially choosing alternative care providers due to financial constraints.

#### Impact of perceived health service quality on help-seeking behaviour

The perceived quality of healthcare services significantly impacts decisions to seek medical treatment. Long waiting times, unreliable diagnosis, poor communication, and inconsistent availability of drugs often deter families from seeking care at public healthcare facilities. Patients often turned to traditional healers to avoid delays and high costs.

#### Lack of early recognition

As described in the section related to ‘knowledge and perception of meningitis’, many families did not recognise the early symptoms of the disease and only sought medical attention when the condition had progressed to a critical stage.

In some cases, patients and carers did not suspect meningitis until it was diagnosed at the hospital. Previous experience with the disease was rare, and many families failed to recognise the seriousness of the symptoms early on.

#### Recognition of severity as a trigger for help-seeking initiation

An important aspect that facilitated the decision to seek medical care in LMICs was often influenced by the severity of the illness. Carers in LMICs often base their decision to seek medical treatment on the visible deterioration of the patient’s condition rather than early symptoms. As long as the illness allowed the patient to maintain some level of normal activity or is amenable to traditional methods of treatment, carers may delay treatment. However, when the patient’s illness becomes severe—marked by the inability to perform basic tasks or a noticeable decline in strength—carers are more likely to seek biomedical care:

*When he became very sick, he failed to sit down, he failed to stand up... It’s when he (neighbour) suggested, ‘this person should not stay here.’ Yes, I can say the severity of the illness was what made us go.* (Desmond, 2013)

Some carers also stated that their decision to seek medical care was often motivated by the disruption of the patient’s ability to fulfil daily household duties. This disruption resulted in urgent action.

##### Hospitalisation period

###### Healthcare workers

**Experiences and values**

Data around experiences of HCWs were available from LMICs only.

#### Challenges in diagnosing meningitis

HCWs from a malaria-endemic region noted some challenges in diagnosing meningitis, despite claiming sufficient knowledge of its signs and symptoms. Challenges stemmed from misdiagnoses of meningitis as malaria in both adult and paediatric cases, leading to the initial prescription of anti-malarial treatment.

#### Miscommunication between healthcare workers and patients/carers

HCWs revealed dismissive attitude to patients’ and carer’s concerns, particularly when they returned multiple times with unresolved symptoms. In some cases, patients and caregivers, especially mothers of sick children, were blamed for the persistence of illness, with HCWs assuming that the prescribed medications had not been properly administered. Consequently, HCWs failed to take patient or carer concerns seriously, dismissing their reports of persistent symptoms without adequate investigation. This underestimation may prevent timely diagnosis of meningitis, leading to delays in treatment.

**Barriers and facilitators**

We identified two themes describing barriers to care delivery and one describing actions that may result in facilitation, with all evidence coming from LMICs.

#### Systemic and operational barriers in healthcare organisation

One of the challenges faced by healthcare centres in both epidemic and non-epidemic settings in LMICs was the shortage of medicines, including those that are supposed to be provided free of charge. The inconsistent availability of medications not only delayed treatment but also diminished patients’ trust in the healthcare system. Moreover, in an epidemic setting, HCWs were often unaware of official guidelines regarding different payment schedules, leading to increased out-of-pocket expenses for medications, despite official government policy.

HCWs faced barriers in performing critical diagnostic procedures, such as lumbar punctures (LPs). These barriers included time constraints, delays caused by the need for additional procedures, such as computed tomography scans, and difficulties in locating necessary consumables or even patients, which may indicate suboptimal hospital logistics. In some cases, a lack of proper sterile conditions during LP also contributed to the reluctance of HCWs to perform the procedure. This may result in missed opportunities for timely diagnosis and treatment.

Some doctors reported incorrect knowledge about LP contraindications, which affected their willingness to perform LP. The uncertainty in expertise was further underscored by the desire to consult colleagues about the need for LP, potentially causing additional delays in the procedure. Moreover, some HCWs indicated that a perceived lack of expertise among doctors could lead to their reluctance to undergo LP themselves or to recommend the procedure to others:

*No, I would not do it [an LP on myself] and, no, I would not advise it because I am not comfortable with the expertise of most doctors.* (Elafros, 2022)

#### Community apprehensions influencing healthcare workers’ decision-making

HCWs were sometimes hesitant to perform LPs on patients in critical condition due to fears that, if the patient died soon after, other caregivers and patients would perceive the procedure as the cause of death. This worry reflected a broader community belief that LP can be fatal.

In situations where a child is already in a terminal condition, some physicians opted not to perform the procedure, believing it would only exacerbate existing fears:

*If a child is in the terminal stage, then it is appropriate not to do it, as it will reinforce the belief of death.* (Elafros, 2022)

In some cases, this hesitancy was dictated by a personal, perhaps emotional, judgement of HCWs. While caregivers were often more willing to consent to LPs later in the admission, HCWs believed that at this stage, the procedure would no longer have an impact on patient outcomes.

#### Consent practices facilitating lumbar puncture delivery

HCWs minimised or even omitted the potential risks of the procedure to encourage caregivers to consent to LPs and prevent refusal. HCWs framed LPs as safe and necessary for accurate diagnosis, which helped alleviate patients’ and caregivers’ concerns:

*I told them, I am going to do a lumbar puncture and it’s safe.* (Elafros, 2022)

It was noted that consent was obtained verbally rather than in writing, which was a norm in this area. By omitting written consent, HCWs believed to prevent patients from having misconceptions about the procedure. While consent was obtained only verbally, LP refusal was formally documented in medical records.

HCWs found that providing a simple explanation of the LP procedure and allowing for multiple conversations with caregivers increased the likelihood of obtaining consent. In some cases, however, HCWs saw these discussions as protracted and delaying urgent patient care and, therefore, overlooked the consent process altogether. While this practice may pragmatically facilitate patient throughput, it simultaneously highlights the ethical challenges of navigating profound relational barriers and the potential compromise of the principle of fully informed consent.

The communal environment in hospitals provided an opportunity for HCWs to use the experiences of other patients who have successfully undergone LPs to reassure caregivers. Seeing other patients go through the procedure without complications may help build a sense of trust and confidence in the caregivers, making them more likely to consent.

##### Patients, carers, and community members

Two themes around patient/carer experience in LMICs and four in HICs were synthesised.

**Experiences and values**

#### Perceptions of lumbar puncture outcomes

Death and paralysis were the most reported outcomes of LPs. Patients and caregivers often believed that complications occurred only when the procedure was performed by an inexperienced clinician. Caregivers believed that death was tied to the timing of the procedure, particularly if the LP was performed too late or when the patient was perceived as too weak. Paralysis was linked to the positioning of the patient during or after the LP.

These fears were reinforced by personal or family experiences. One caregiver reported the death of a relative following an LP as a reason for concern:

*I fear that my child might die because my father died after the procedure.* (Elafros, 2022)

Despite these fears, patients and caregivers commented on the improvement of LP outcomes in recent years.

#### Economic impact of medical treatment on families

Families cited the overwhelming financial burden of hospitalisation and medical treatment, particularly due to the high cost of prescriptions. The financial strain was especially severe for those caregivers who were unemployed. These families were unable to afford basic household expenses, with all available funds directed towards the child’s treatment:

*I borrowed some money from my friend, but I only managed this situation with difficulties because I was unemployed. The hospitalization was a heavy financial charge and now we haven’t got the household expenses. I used my only money for the child’s treatment.* (Griffiths, 2012)

#### Need for healthcare workers’ greater awareness/alertness and rapid decision-making

Both adult and paediatric patients in HICs initially received other diagnoses, suggesting suboptimal preparedness of HCWs for meningitis. Patients and carers recognised the issue and emphasised the need for greater vigilance and faster responses from medical staff. Families described how healthcare providers initially dismissed or misinterpreted symptoms, which led to frustration, especially when they felt symptoms were clear:

*It lasted 7 to 8 hours and when they (the healthcare providers) got to my room, there was panic on board... it’s not normal. They could have considered my symptoms. Neck pain, intolerance to light and sound, vomiting and fever, they could have done at least one lumbar puncture to remove the doubt.* (Scanferla, 2020)

Parents reported that sometimes HCWs were not well-prepared to manage meningitis, making families feel concerned or desperate:

*The doctors didn’t know much.... When I did ask the doctor on the ward at the time something about it [IMD], she said she couldn’t answer me because she wasn’t familiar with it and she’d never treated it... very annoyed with the fact that they didn’t know what they were doing because then they’re treating him for something that they know nothing about... frustrating when you’re in the moment and you had a question and they were like... “we don’t know”.* (Wisemantel, 2018)

In contrast, parents and carers were grateful and satisfied with the provided care when healthcare professionals responded to meningitis with quick actions, for example, by promptly referring to a hospital after accurate recognition or by initiating empirical treatment.

#### Importance of appropriate communication and information from healthcare workers

Carers in HICs reported feeling frustrated and distressed when HCWs provided insufficient or vague explanations about meningitis, both in general terms and in relation to their child’s condition. A lack of detailed communication during hospital stays led families to seek information on their own. Others felt that health workers were dismissive or failed to address their concerns.

In contrast, parents whose healthcare providers took the time to explain the situation clearly noted feeling satisfied with the support and a sense of control over the situation.

*It was faultless from start to finish - from the paramedic to the hospital. They kept us informed - the good and the bad.* (Sweeney, 2013)

#### Parental emotional turmoil during hospitalisation

The experience of having a child diagnosed with meningitis was described as an intense emotional journey for parents and carers. Parents described feelings of shock, disbelief, and fear upon facing the meningitis diagnosis. The initial emotions persisted throughout the hospitalisation as many parents were confused about what could possibly happen and fearing for their child’s life:

*…I was completely shocked by his condition. I thought he was dying. In fact, I know he barely avoided death. I’ve always felt that my son was close to death.* (Scanferla, 2021)

The experience of hospitalisation in intensive care unit (ICU) added to the emotional burden of parents whose children had a particularly severe illness. Parents were distressed, anxious, and emotionally unprepared to see changes in their child’s appearance and behaviour caused by support equipment and treatments:

*I freaked because... he was hooked up to everything, all the monitors... I had a bit of a breakdown*. (Wisemantel, 2018)

Although many parents described the experience of hospitalisation as traumatising, some of them found a relief once their child was in a controlled hospital environment and receiving medical attention and care.

#### Coping strategies and emotional support during hospitalisation

Parents in HICs reported that HCWs, particularly nurses, helped provide emotional support during their child’s hospital stay. Parents who received attentive care from nurses felt supported and expressed appreciation for the relationships they developed with them.

However, the quality of support from HCWs varied, with some participants feeling that more consistent and structured support, such as counselling services, was needed. Emotional support was especially critical at the time of diagnosis and hospitalisation in the intensive care unit, where the lack of psychological support left parents struggling to cope.

Some parents expressed gratitude for the practical assistance provided by hospitals. Special accommodations, like offering a place for parents to stay at the hospital, eased stress and helped families feel more supported. Despite the importance of the support from HCWs, most parents cited family and friends as key sources of emotional and practical support during hospitalisation. For many, this support was seen as sufficient, and they did not feel the need for additional professional support.

**Barriers and facilitators**

Two themes describing barriers to care uptake and one that may reflect actions facilitating the uptake were synthesised, with all evidence coming from LMICs and the Elafros et al. [21] study only.

#### Fear of complications as a barrier to lumbar puncture uptake

Fear of LP-related complications, particularly death and paralysis, was a significant barrier to consent for the procedure. This fear was reported universally among adult patients and caregivers, and some paediatric caregivers, and was influenced by prior negative experiences.

HCWs acknowledged that LPs had gained a negative reputation during the human immunodeficiency virus epidemic when the procedure was associated with high mortality rates. This historical context contributed to a deep-rooted apprehension surrounding LP, making it challenging for healthcare workers to obtain consent.

#### Reliance on shared decision-making

The importance of shared decision-making within families and communities played a role in consenting to LP. Caregivers consulted extended family members, especially older male relatives or grandmothers. This reliance on collective decision-making could lead to delays, especially when the family lived far away and was not immediately available to consult. Some patients rescinded their consent after being influenced by their family or other patients and caregivers in the hospital ward, further complicating care delivery.

#### Patients’ values driving consent to lumbar puncture

Confidence in the competence of the treating physician influenced the consent process, enabling patients and caregivers to agree to LP. Trust in the healthcare provider’s technical skills and intentions helped patients overcome fears associated with the procedure.

Additionally, patients and caregivers cited obtaining a clear diagnosis as a strong motivator for consenting to the LP.

Concerns about patient’s health and disease severity and progression also stimulated families to proceed with the LP, often as a last resort for adult patients. As symptoms worsened, patients and caregivers saw the procedure as a necessary step in identifying the underlying cause of the illness.

### Meningitis sequelae

#### Healthcare workers

No studies in either LMICs or HICs provided any data regarding experiences of HCWs or described barriers/facilitators to care delivery.

#### Patients, carers, and community members

Six themes around patient/carer experience were synthesised with one shared between LMICs and HICs, one LMIC-related, and four HIC-related.

#### Experiences and values

##### Multifaceted impact of meningitis on physical, mental, and social well-being

Meningitis survivors from LMICs were experiencing long-term physical impairments, cognitive and mental health decline, and substantial changes in social roles and personal independence.

Older adults reported deterioration in physical health, including heart problems, partial paralysis, hearing and visual impairments, and decline in cognitive and mental functioning:

*I think I am experiencing mental fatigue because what I could do in the past with ease, I cannot do them anymore... I do not know anything. I do not know what is happening around me... Everything is blank* (Mahmoud, 2022)

In children, the disease severely impacts developmental progress, including motor and speech and language. Some parents also noted behavioural changes and inability of children to care for themselves due to meningitis consequences.

*The child does not speak, does not walk, he cannot even sit down. At the age of eight years he cannot do anything, but is totally dependent. Since the attack of meningitis he is no longer at school.* (Griffiths, 2012)

Meningitis sequelae severely interfered with social participation and personal independence, which is especially evident in the elderly. Increased dependency was associated with a burden on families, who sometimes struggled to support their loved ones due to economic and social limitations.

In HICs, the impact of meningitis sequelae was no less devastating, with survivors experiencing severe physical sequelae, emotional trauma, and disruptions to their life plans and careers.

Meningitis survivors in HICs faced physical impairments, including amputations, cardiac, hearing, and vision problems in adults and neurodevelopmental delay in children. Cognitive impairments, such as memory loss and executive dysfunction, were also reported.

The emotional toll of meningitis sequelae was also severe, with survivors experiencing depression, unresolved grief, and anger. The psychological trauma of the disease extended beyond physical health, causing phobias, social isolation, and long-term insecurity.

Participants reported how meningitis damaged their future, professional and personal lives. Some meningitis survivors had to change careers, education and family plans, while others experienced social isolation and a decline in personal motivation. Caregivers also faced disruptions, with family members often needing to quit their jobs to care for affected individuals.

*Everything was going great, I was going to settle down, and it was the first time in my life that everything was going well. The meningitis ruined everything...* (Scanferla 2020)

##### Experiences with providing care for meningitis sequelae

This theme, synthesised from the caregiver experiences in LMICs, was characterised by difficulties balancing caregiving responsibilities for ailing family members with the need to maintain jobs to afford essential medications and healthcare services. This tension between caregiving and financial obligations caused some frustration:

*My siblings are there despite not having much time because they are still in school. I am here in Lagos working. If I do not work, how will I pay the hospital bill? I must confess that I am not happy leaving my mum in such conditions, but what can I do? We need money too to take care of her.* (Mahmoud, 2022)

Caregiving also carried significant financial implications for families of children affected by meningitis sequelae. For some parents, the burden was associated with the necessity of hiring special personnel, while others experienced financial difficulties due to the need to leave their employment.

Caregivers reported feelings of frustration and isolation due to their caregiving responsibilities, which cut them off from their social circles and impacted their emotional well-being.

Despite the challenges, some caregivers felt happier to return the care they once received from their parents.

##### Long-term psychological impact of hospitalisation

Patients in HICs developed trauma and phobias related to their hospitalisation. Some described phobias of hospitals and medical procedures as a direct result of previous traumatic hospitalisation for meningitis:

*I’m (...) afraid of blood tests now. I hate it (...) it’s true that when I find myself in a hospital room, it makes me think about it again (...), blood tests, I have a real problem with them. I pass out every time (...). When I had my last surgery, that was the hardest thing (...) to inject me, it took an hour to get the catheter in (...). The image of my blue arms where they were trying to stick it in has stayed with me... It was horrible! It’s been 5 years and I think it’ll be a long time before I forget.* (Scanferla, 2020)

For some children, fear of medical personnel persisted long after their recovery. One mother shared that her child would cry and scream whenever they saw her in her nurse’s uniform, even 2 years after hospitalisation.

Caregivers also faced the psychological impact of meningitis hospitalisation, manifesting in depressive symptoms and feelings of helplessness. Some parents revealed that they experienced depression that required medication and therapy.

Participants reflected on the lasting impact of the traumatic experience, noting that despite the passage of time, memories of helplessness during hospitalisation persisted. Some carers also described how the illness altered their lives permanently, extending its effects far beyond the period of hospitalisation:

*It’s with me every day and it fuels every aspect of how I parent her. It feels like I’ve taken a deep breath and never exhaled.* (Sweeney, 2013)

##### Parental concerns about potential consequences of meningitis

Parents in HIC were concerned about the future challenges their children might face as a result of meningitis. They were also expressing anxiety over their child’s developmental progress, comparing them to peers and siblings, and worrying about potential behavioural issues related to the disease.

*Now I’m a little better, but there are always times when I think about the future, and I still have fears and anxieties about what might happen later on.* (Scanferla, 2021).

##### Need for care continuity, education, and support

Parents in HICs felt the need for follow-up appointments, as well as educational and psychological support, to ensure their child’s full recovery. Some reported feeling confused and worried due to the lack of follow-up.

Parents felt overwhelmed and struggled to cope with the emotional toll of caring for a child with meningitis sequelae, expressing the need for post-hospitalisation psychological support and counselling.

Some parents also highlighted a lack of knowledge about meningitis sequelae and a desire to receive more information and education regarding potential complications.

##### Perceptions of quality of aftercare for meningitis sequelae

Parents in HICs reported challenges in accessing sufficient or timely aftercare due to staff shortages or budget restrictions. Some parents shared their frustration with inadequate customisation of rehabilitation services, for example, prosthetic limbs. Parents also felt that professionals failed to communicate effectively or listen to their concerns.

On the other hand, when parents were satisfied with the provided rehabilitation services, aftercare was tailored and suitable for their child’s needs. Effective communication and listening to parents’ expectations played a crucial role in good care.

##### Barriers and facilitators

Five themes describing barriers to care delivery (three in LMICs and two in HICs) and one from HICs reflecting facilitators were synthesised.

##### Balancing marital and domestic responsibilities and caregiving

Adult daughters faced the challenge of balancing marital and domestic responsibilities while caring for ailing parents. Marriage distances women from their parental homes, making it difficult for them to provide hands-on care.

##### Preference for home care over institutionalisation

Caregivers from LMICs reported a preference for home-based care over institutionalisation of elderly parents. They believed institutionalisation would worsen the health of their parents, while family care provides care and emotional support:

*Family is the best home where they can see their loved one... However, there would be a significant problem if family members are not available to provide care and love to them.* (Mahmoud, 2022).

This poses a barrier to considering formal care facilities, even when caregivers are unable to provide consistent care due to distance or personal responsibilities.

##### Financial burden as a barrier to aftercare services uptake

Financial constraints were already described in sections dedicated to the acute phase. Similarly, financial aspects were a significant barrier to accessing aftercare services for children who have experienced meningitis sequelae. Caregivers cited the high cost of transport, hospital consultations, and necessary medical devices as reasons for discontinuing aftercare.

These financial burdens prevented some caregivers from accessing essential treatments, including physiotherapy and hearing aids, precluding any rehabilitation for children with long-term consequences of meningitis.

##### Lack of appreciation for less apparent sequelae of meningitis

Parents noted a lack of recognition and understanding of the less visible psychosocial and cognitive after-effects of meningitis. This lack of awareness about sequelae created barriers to accessing support services, especially in educational settings.

Young age acted as an additional barrier to gaining access to aftercare because of the difficulty testing young children, misconceptions about the needs of disabled children, and challenges in predicting cognitive after-effects at the time of discharge.

##### Systemic and organisational barriers to aftercare

Caregivers faced systemic barriers when attempting to navigate the health and social care systems to access aftercare services for their children. While most parents were able to access the support their child needed, this was sometimes achieved only after significant effort. Parents had to ‘learn the language’ of the system, navigating complex bureaucratic processes with little guidance. This added an extra layer of stress and frustration, especially after their child’s discharge from the hospital.

The challenge of navigating these systems was compounded by a lack of communication between different specialists, which resulted in unresponsive care.

Budget restrictions and staff shortages also exacerbated delays in receiving aftercare. In addition to these structural issues, the administrative burden of accessing services was another significant barrier. Some caregivers highlighted the psychological toll of constantly having to prove the legitimacy of their requests for services:

*I always say the biggest handicap is administration. (…) you have to keep fighting.* (Scanferla, 2021)

Parents also faced difficulties gaining access to specific services, such as hearing aids or mobility aids, due to restrictive criteria that did not always account for the child’s needs:

*She was told she would only have thirty five per cent hearing, but then told that she couldn’t at that time apply for a hearing aid because she was borderline... so we went ahead and got one for her.* (Clark, 2013)

##### Third parties as facilitators of meningitis sequelae aftercare

Schools proved to be an important facilitator of support provision for children with long-term sequelae of meningitis, particularly when children had special educational needs. These formalised plans enabled more streamlined access to aftercare services, reducing delays and ensuring that children received the necessary support over an extended period.

Another facilitator in the aftercare process was the role of multidisciplinary team meetings. These meetings, which brought together parents, school staff, and healthcare professionals, improved communication and cooperation, ensuring that the child’s diverse needs were met in a coordinated and effective manner.

For parents who were able to overcome organisational barriers, having a key point of contact who was proactive in managing their child’s care played a crucial role. In some cases, the presence of a consistent healthcare professional, such as a consultant, ensured that care was well-planned and tailored to the child’s specific needs.

## Discussion

This systematic review is the first to comprehensively synthesise qualitative evidence on the experiences of meningitis care across diverse settings, offering valuable insights into patient, caregiver, and healthcare provider perspectives. By including studies from both HICs and LMICs, the review provides a global perspective on the barriers and facilitators of meningitis care and informs the WHO guideline development process to ensure impact of synthesis on healthcare. The thematic synthesis allowed for a rich and nuanced understanding of how cultural, financial, and systemic factors influence care-seeking behaviours and healthcare delivery in different contexts. The findings highlighted the urgent need for evidence-informed policies tailored to different healthcare settings to improve meningitis diagnosis and management throughout care pathway.

In LMICs, delays in care-seeking are frequently driven by financial barriers, limited healthcare accessibility, and sociocultural factors, including reliance on traditional healing practices. Previous research has demonstrated that targeted health education campaigns and community engagement strategies can improve early care-seeking behaviours [27]. Policymakers may consider focusing on strengthening healthcare infrastructure, implementing financial protection schemes such as community-based health insurance, and integrating biomedical and traditional care approaches to foster greater trust in health systems. Further development and implementation of meningitis surveillance programmes can facilitate early detection and response to outbreaks [28].

In HICs, key challenges included delayed symptom recognition, miscommunication between healthcare providers and patients, and insufficient psychological support for meningitis survivors and their families. Standardising early recognition training for primary care physicians and emergency clinicians is crucial, given that symptom variability often leads to misdiagnoses [29]. Policymakers should prioritise structured communication frameworks to improve patient-provider interactions and ensure families receive comprehensive information at all stages of care which is associated with improved outcomes [30]. Additionally, integrating mental health services into meningitis treatment pathways can provide essential emotional and psychological support, particularly for caregivers who experience long-term distress following relative’s diagnosis [31].

At the global level, the findings reinforce the need for harmonised guidelines that address systemic barriers in meningitis care across diverse healthcare settings. The WHO ‘Defeating Meningitis by 2030’ roadmap offers a strategic framework for reducing meningitis burden worldwide, emphasising the importance of vaccine accessibility, improved case management, and surveillance. In line with this, national health authorities should collaborate to ensure as much as possible that local policies account for both structural and sociocultural factors influencing care uptake and provision.

To facilitate the direct translation of synthesised qualitative evidence into operational guidance, we present explicit, context-specific suggestions derived from the four overarching analytical constructs identified across HIC and LMIC (Table 2). These suggestions address structural, workforce, and relational interventions required to strengthen the meningitis care pathway and are framed to provide clear considerations for evidence-to-decision frameworks.

**Table 2:** Policy and practice suggestions derived from qualitative evidence synthesis

Analytical construct	Phase	Key qualitative finding (barrier/need)	Context	Policy suggestion (actionable strategy)
Diagnostic uncertainty (A)	Acute	Healthcare workers (particularly GPs) struggle to recognise atypical symptoms, leading to diagnostic delays	HIC	Mandate high-fidelity simulation training for frontline staff (GPs, ED) focused on identifying rapid deterioration and distinguishing non-specific presentations when classic signs are absent
Initial delays (A)	Acute	Financial constraints (transport and treatment costs) and reliance on traditional healing critically delay care-seeking	LMIC	Implement zero-fee policies for suspected meningitis cases at the point of first contact; establish subsidised emergency transport mechanisms to health facilities; and launch culturally sensitive community awareness campaigns via trusted local leaders
Systemic barriers (B)	Acute	Lack of experience with parenteral antibiotics and protocol-related anxiety delay pre-hospital treatment	HIC	Standardise pre-hospital care protocols, ensuring mandatory recurrent training and certified access to appropriate parenteral antibiotics for primary care providers
Systemic barriers (B)	Acute	Supply shortages (medicines, consumables) and inadequate laboratory capacity impede timely diagnosis (e.g. lumbar puncture)	LMIC	Prioritise ring-fenced national budgets for meningitis care logistics, ensuring consistent availability of essential diagnostics and first-line antibiotics, particularly during epidemic seasons
Relational burden (C)	Acute	Community apprehension leads healthcare workers to compromise informed consent for lumbar puncture by minimising risks	LMIC	Develop standardised, empathetic consent protocols that mandate staged discussions of risks, benefits, and diagnostic necessity, thereby fostering trust without compromising ethical standards
Relational burden (C)	Acute	Miscommunication, blame, and vague explanations heighten parental frustration and distress during hospitalisation	HIC	Implement standardised, empathetic communication protocols, ensuring designated liaison staff provide consistent and comprehensive information to families throughout the acute phase
Sequelae/access (D)	Long-term	Survivors and carers experience severe emotional trauma, anxiety, and long-term psychological impacts	Global (HIC focus)	Establish mandatory, integrated psychological screening and referral pathways for all survivors and primary caregivers post-discharge, including access to specialist trauma counselling and peer support networks
Sequelae/equity (D)	Long-term	Families face systemic difficulties navigating health, social, and educational support for less apparent sequelae (cognitive and psychosocial deficits)	HIC	Designate multidisciplinary case managers or navigators to support families in accessing educational, rehabilitative, and social services, ensuring timely and equitable provision of long-term care

Overarching analytical constructs: (A) diagnostic uncertainty and disparities in initial help-seeking; (B) systemic and operational constraints on acute care delivery; (C) the relational and psychological burden of meningitis; and (D) barriers to long-term equity and continuity of care

*ED*, emergency department; *GP*, general practitioner; *HIC*, high-income country; *LMIC*, low- and middle-income country

This review is associated with several limitations. The inclusion of only English-language studies may have resulted in the exclusion of relevant research published in other languages, particularly from LMICs. Additionally, the variability in study designs, participant demographics, and healthcare settings may limit the transferability of some findings. While thematic synthesis allows for the integration of diverse qualitative data, the heterogeneity of the included studies may have influenced the interpretation of the results. Future research could benefit from including non-English studies and focusing on specific subgroups, such as paediatric populations, rather than parental proxy-reported experiences, to provide more detailed insights into particular aspects of meningitis care delivery and relevant barriers.

## Conclusions

Available evidence underscores the need for context-specific interventions to address the diverse challenges faced in meningitis care across HICs and LMICs. In LMICs, efforts should focus on improving healthcare access, reducing financial barriers, and addressing cultural misconceptions about medical procedures. In HICs, enhancing early recognition of meningitis symptoms, improving patient-provider communication, and integrating psychological support services into care pathways are crucial for improving patient outcomes.

Policymakers, public health experts, and healthcare providers should collaborate to implement these strategies, informed by the qualitative evidence presented in this systematic review, to improve meningitis care globally and reduce the long-term burden of the disease.

## Supplementary Information

Additional file 1. Box 1 Search strategy. Box 2 Reflexivity statement. Table S1 Critical Appraisal Skills Programmechecklist for qualitative data. Table S2 Summary of qualitative findings and GRADE-CERQual assessment. Table S3 Themes synthesised from the available evidence.

## Data Availability

Data is provided within the manuscript or the additional file.

## Abbreviations

CASP: Critical Appraisal Skills Programme
CERQual: Confidence in the Evidence from Reviews of Qualitative Research
CNS: Central nervous system
Com: Community
CSM: Cerebrospinal meningitis
GP: General practitioner
HCW: Healthcare worker
HICs: High-income countries
IMD: Invasive meningococcal disease
IQR: Interquartile range
LMICs: Low- and middle-income countries
LP: Lumbar puncture
MeSH: Medical Subject Headings
*N*: Number
NR: Not reported
P: Patient
PICU: Paediatric intensive care unit
PRISMA: Preferred Reporting Items for Systematic Reviews and Meta-Analyses
SD: Standard deviation
UK: United Kingdom
USA: United States of America
WHO: The World Health Organization
