# Supplementary material for: Experiences and service-uptake factors of receiving and providing care for meningitis and its sequelae: a qualitative systematic review
Source: BMC Med. 2026 Apr 13;24:385. doi: 10.1186/s12916-026-04765-4 (PMC13343739; doi:10.1186/s12916-026-04765-4)
Supplement: Supplementary file 1 — Additional file 1. Box 1 Search strategy. Box 2 Reflexivity statement. Table S1 Critical Appraisal Skills Programmechecklist for qualitative data. Table S2 Summary of qualitative findings and GRADE-CERQual assessment. Table S3 Themes synthesised from the available evidence. [file 12916_2026_4765_MOESM1_ESM.docx]

**SUPPLEMENTARY MATERIAL**

**Experiences and service-uptake factors of receiving and providing care for meningitis and its sequelae: A qualitative systematic review**

Margarita Andreeva ^1#^, Maria Pyatnitskaya ^2#^, Karina Kochneva ^3#^, Ekaterina Sviatskaia ^3#^, Maria Spryshkova ^3#^, Melissa Meyer ^4^, Dina Baimukhambetova ^5^, Mark Kosenko ^5^, Anna Mursalova ^5^, Nina Avdeenko ^5^, Elena Kondrikova ^5^, Jonathan Zheng ^6^, Ka Yan Cheung ^7^, Audrey Dunn Galvin ^4^, Tom Jewell ^8^, Zoe Moula ^8^, Sarah Neill ^9^, Mikhail S Zinchuk ^3^, Alla Guekht ^3^, Daniel Munblit ^5,8#^*

1. University of British Columbia, Vancouver, Canada
2. Erasmus School of Health Policy & Management, Rotterdam, Netherlands
3. Research and Clinical Center for Neuropsychiatry, Moscow, Russia
4. University College Cork, Cork, Ireland
5. Department of Paediatrics and Paediatric Infectious Diseases, Institute of Child’s Health, I.M. Sechenov First Moscow State Medical University, Sechenov University, Moscow, Russia
6. Department of Electrical and Electronic Engineering, Imperial College London, London, UK
7. Faculty of Medicine, Imperial College London, London, UK
8. Care for Long Term Conditions Division, Florence Nightingale Faculty of Nursing, Midwifery and Palliative Care, King's College London, London, United Kingdom
9. University of Plymouth, Plymouth, United Kingdom

# Box 1: Search strategy

1. Database: Embase (Elsevier)

URL: https://www.embase.com/#advancedSearch/

Date Searched: 13.02.2024

| **#** | **Searches** | **Results** |
| --- | --- | --- |
| 1 | ('meningitis'/exp OR (meningiti* OR (Meningococc* NEAR/3 (infection* OR disease*))):ti,ab) | 152502 |
| 2 | bacterial meningitis'/de OR 'epidemic meningitis'/exp OR 'Escherichia coli meningitis'/exp OR 'group B streptococcal meningitis'/exp OR 'Haemophilus meningitis'/exp OR 'leptospiral meningitis'/exp OR 'Listeria meningitis'/exp OR 'Lyme meningitis'/exp OR 'pneumococcal meningitis'/exp OR 'fungal meningitis'/exp OR 'HIV-associated meningitis'/exp OR 'parasitic meningitis'/exp OR 'virus meningitis'/exp OR 'aseptic meningitis'/exp OR 'Staphylococcus aureus'/exp OR 'Staphylococcus'/exp OR 'Enterobacteriaceae'/exp OR 'Streptococcus agalactiae'/exp OR 'Streptococcus pyogenes'/exp OR 'Enterovirus'/exp OR 'Herpesviridae'/exp OR 'herpes virus infection'/exp OR 'Simplexvirus'/exp OR 'Flavivirus'/exp OR 'West Nile virus'/exp OR 'Togaviridae'/exp OR 'Mumps'/exp OR 'Mumps virus'/exp OR 'Orthomyxoviridae'/exp OR 'HIV'/exp OR 'Adenoviridae'/exp OR 'Rubella'/exp OR 'Lymphocytic Choriomeningitis'/exp OR 'Rickettsiales'/exp OR 'Spirochaetales'/exp OR 'Leptospira'/exp OR 'Brucella'/exp OR 'Treponema pallidum'/exp OR 'Coxiella'/exp OR 'Mycoplasma'/exp OR 'Naegleria'/exp OR 'Angiostrongylus'/exp OR 'Coccidioides'/exp OR 'Candida'/exp OR 'Histoplasma'/exp OR 'Blastomyces'/exp OR 'Aspergillus'/exp OR 'Syphilis'/exp OR 'Lyme Disease'/exp OR 'Scrub Typhus'/exp OR ((Bacterial OR bacteraemia OR Viral OR Fungal OR Aseptic OR Parasitic OR community-acquired OR Acute OR fulminat* OR Fulminant OR Sudden-onset) NEAR/5 (meningiti*)):ti,ab,kw,de OR (infectious-meningiti* OR Meningococc* OR Neisseria-meningit* OR N-Meningitidis OR Pneumococc* OR S-pneumoniae* OR Haemophilus-influenzae OR Listeri* OR L-monocytogenes OR Staphylococc* OR Staph-aureus OR Enterobacter* OR Enterococc* OR Escherichia-coli OR E-coli OR Streptococc* OR S-agalactiae* OR S-pyogenes OR Enterovir* OR Coxsackieviruses OR Herpesviridae OR Herpesvirus* OR herpes-virus* OR Varicella-zoster OR flavi-virus* OR Japanese-encephal* OR Tick-borne-encephal* OR Powassan-virus* OR West-Nile-virus OR Togaviridae OR Toga-virus* OR Togavir* OR equine-encephal* OR Bunyavirus* OR crosse-encephal* OR Toscana-virus* OR Reovirus* OR tick-fever* OR paramyxovir* OR Mumps OR morbillivirus* OR parainfluenza* OR Orthomyxovir* OR Influenza OR HIV OR human-immuno-deficienc* OR Adenoviridae OR adenovirus* OR Arenavir* OR Choriomeningit* OR LCMV OR Rickettsi* OR Orientia-spp OR Ehrlichia-spp OR spirochet* OR Borrelia-spp OR B-burgdorferi OR leptospir* OR Treponema-pallidum OR Brucell* OR Coxiella OR Mycoplasma OR spirillum* OR Naegleria OR angiostrongyl* OR Trichinella-spiralis* OR Candida OR Coccidioid* OR Histoplasm* OR Blastomyc* OR Sporothrix* OR Aspergill* OR Lyme OR Syphili* OR Scrub-Typhus OR tsutsugamushi):ti,ab,kw,de | 2793298 |
| 3 | #1 AND #2 | 101270 |
| 4 | qualitative research'/exp OR 'qualitative analysis'/exp OR 'semi structured interview'/exp OR 'grounded theory'/exp OR 'thematic analysis'/exp OR 'observational method'/exp OR 'constant comparative method'/exp OR 'participant observation'/exp OR 'narrative'/exp OR 'field study'/exp OR 'audiovisual recording'/exp OR 'focus group'/exp OR 'interview'/exp OR 'attitude'/exp OR ((experiences OR qualitative OR interview* OR focus-group* OR semi-structured OR semistructured OR phenomenol* OR ethnograph* OR grounded-theory OR grounded-study OR grounded-analysis OR grounded-analyses OR life-story OR life-stories OR content-analysis OR thematic-analysis OR thematic-analyses OR content-analys* OR narrative-analysis OR fieldwork OR field-work OR ((decriptive OR field) NEAR/3 (study)) OR key-informant* OR investigative OR theme* OR Thematic OR participant-observation* OR group-discussion*) OR (("semi-structured" or semistructured or unstructured or informal or "in-depth" or indepth or "face-to-face" or structured or guide*) NEAR/5 (discussion* or questionnaire* OR survey*))):ti,ab | 2264113 |
| 5 | #3 AND #4 | 2307 |

2. Database: Medline (OVID)

URL: Ovid MEDLINE(R) 1946 to January Week 5 2024

Date Searched: 14.02.2024

| **#** | **Searches** | **Results** |
| --- | --- | --- |
| 1 | Meningitis/ OR meningit*.mp. OR ((meningococc*) ADJ3 (infection* OR disease*)) | 77047 |
| 2 | Meningitis, Bacterial/ OR Meningitis, Escherichia coli/ OR Meningitis, Haemophilus/ OR Meningitis, Listeria/ OR Meningitis, Meningococcal/ OR Meningococcal Infections/ OR Meningitis, Pneumococcal/ OR Meningitis, Fungal/ OR Meningitis, Aseptic/ OR Meningitis, Viral/ OR ((Bacterial OR Viral OR Fungal OR Aseptic OR Parasitic OR community-acquired OR Acute OR fulminat* OR Fulminant OR Sudden-onset ) ADJ5 (meningiti*)).ti,ab,kw,kf OR (infectious-meningiti* OR Meningococc* OR Neisseria-meningit* OR N-Meningitidis OR Pneumococc* OR S-pneumoniae* OR Haemophilus-influenzae OR Listeri* OR L-monocytogenes OR Staphylococc* OR Staph-aureus OR Enterobacter* OR Enterococc* OR Escherichia-coli OR E-coli OR Streptococc* OR S-agalactiae* OR S-pyogenes OR Enterovir* OR Coxsackieviruses OR Herpesviridae OR Herpesvirus* OR herpes-virus* OR Varicella-zoster OR flavi-virus* OR Japanese-encephal* OR Tick-borne-encephal* OR Powassan-virus* OR West-Nile-virus OR Togaviridae OR Toga-virus* OR Togavir* OR equine-encephal* OR Bunyavirus* OR crosse-encephal* OR Toscana-virus* OR Reovirus* OR tick-fever* OR paramyxovir* OR Mumps OR morbillivirus* OR parainfluenza* OR Orthomyxovir* OR Influenza OR HIV OR human-immuno-deficienc* OR Adenoviridae OR adenovirus* OR Arenavir* OR Choriomeningit* OR LCMV OR Rickettsi* OR Orientia-spp OR Ehrlichia-spp OR spirochet* OR Borrelia-spp OR B-burgdorferi OR leptospir* OR Treponema-pallidum OR Brucell* OR Coxiella OR Mycoplasma OR spirillum* OR Naegleria OR angiostrongyl* OR Trichinella-spiralis* OR Candida OR Coccidioid* OR Histoplasm* OR Blastomyc* OR Sporothrix* OR Aspergill* OR Lyme OR Syphili* OR Scrub-Typhus OR tsutsugamushi).ti,ab,kw,kf | 1411308 |
| 3 | Qualitative Research/ OR Grounded Theory/ OR Observational Study/ OR Anthropology, Medical/ OR Narrative Medicine/ OR Narration/ OR Sound Recordings/ OR Focus Groups/ OR "Interviews as Topic"/ OR Interview/ OR Personal Narrative/ OR Attitude/ OR "Anecdotes as Topic"/ OR ((experiences OR qualitative OR interview* OR focus-group* OR semi-structured OR semistructured OR phenomenol* OR ethnograph* OR grounded-theory OR grounded-study OR grounded-analysis OR grounded-analyses OR story OR stories OR content-analysis OR thematic-analysis OR thematic-analyses OR content-analys* OR narrative-analysis OR fieldwork OR field-work OR ((decriptive OR field) ADJ3 (study)) OR key-informant* OR investigative OR theme* OR Thematic OR participant-observation* OR group-discussion*) OR (("semi-structured" or semistructured or unstructured or informal or "in-depth" or indepth or "face-to-face" or structured or guide*) ADJ5 (discussion* or questionnaire* OR survey*))).ti,ab | 1229724 |
| 4 | 1 and 2 and 3 | 889 |

3. Database: APA Psycinfo (Ebscohost)

URL: ebscohost.com

Date Searched: 15.02.2024

| **#** | **Searches** | **Results** | **Column1** |
| --- | --- | --- | --- |
| 1 | (DE "Meningitis") OR (TX meningiti*) OR TX ((meningococc*) N3 (infection* OR disease*)) | 1 990 |  |
| 3 | (DE "Qualitative Measures" OR DE "Qualitative Methods" OR DE "Qualitative Methods" OR DE "Focus Group" OR DE "Grounded Theory" OR DE "Interpretative Phenomenological Analysis" OR DE "Narrative Analysis" OR DE "Semi-Structured Interview" OR DE "Thematic Analysis" OR DE "Storytelling" OR DE "Anthropology" OR DE "Ethnography" OR DE "Ethnology" OR DE "Narratives" OR DE "Attitudes" OR DE "Interviews" OR DE "Cognitive Interview" OR DE "Focus Group Interview" OR DE "Psychodiagnostic Interview" OR DE "Semi-Structured Interview") OR (TX (experiences OR qualitative OR interview* OR focus-group* OR semi-structured OR semistructured OR phenomenol* OR ethnograph* OR grounded-theory OR grounded-study OR groundeattituded-analysis OR grounded-analyses OR story OR stories OR content-analysis OR thematic-analysis OR thematic-analyses OR content-analys* OR narrative-analysis OR fieldwork OR field-work OR ((decriptive OR field) N3 (study)) OR key-informant* OR investigative OR theme* OR Thematic OR participant-observation* OR group-discussion*) OR (("semi-structured" or semistructured or unstructured or informal or "in-depth" or indepth or "face-to-face" or structured or guide*) N5 (discussion* or questionnaire* OR survey*))) | 1 522 896 |  |
| 4 | 1 and 2 | 145 |  |
| 2 | (MH "Meningitis, Bacterial") OR (MM "Anthrax Meningitis") OR (MM "Meningitis, Listeria") OR (MM "Meningitis, Meningococcal") OR (MM "Meningitis, Pneumococcal") OR (MH "Meningitis, Viral") OR (MH "Meningitis, Fungal") OR TX ((Bacterial OR Viral OR Fungal OR Aseptic OR Parasitic OR community-acquired OR Acute OR fulminat* OR Fulminant OR Sudden-onset ) N5 (meningiti*)) OR TX (infectious-meningiti* OR Meningococc* OR Neisseria-meningit* OR N-Meningitidis OR Pneumococc* OR S-pneumoniae* OR Haemophilus-influenzae OR Listeri* OR L-monocytogenes OR Staphylococc* OR Staph-aureus OR Enterobacter* OR Enterococc* OR Escherichia-coli OR E-coli OR Streptococc* OR S-agalactiae* OR S-pyogenes OR Enterovir* OR Coxsackieviruses OR Herpesviridae OR Herpesvirus* OR herpes-virus* OR Varicella-zoster OR flavi-virus* OR Japanese-encephal* OR Tick-borne-encephal* OR Powassan-virus* OR West-Nile-virus OR Togaviridae OR Toga-virus* OR Togavir* OR equine-encephal* OR Bunyavirus* OR crosse-encephal* OR Toscana-virus* OR Reovirus* OR tick-fever* OR paramyxovir* OR Mumps OR morbillivirus* OR parainfluenza* OR Orthomyxovir* OR Influenza OR HIV OR human-immuno-deficienc* OR Adenoviridae OR adenovirus* OR Arenavir* OR Choriomeningit* OR LCMV OR Rickettsi* OR Orientia-spp OR Ehrlichia-spp OR spirochet* OR Borrelia-spp OR B-burgdorferi OR leptospir* OR Treponema-pallidum OR Brucell* OR Coxiella OR Mycoplasma OR spirillum* OR Naegleria OR angiostrongyl* OR Trichinella-spiralis* OR Candida OR Coccidioid* OR Histoplasm* OR Blastomyc* OR Sporothrix* OR Aspergill* OR Lyme OR Syphili* OR Scrub-Typhus OR tsutsugamushi) | 278 500 | NOT USED |

4. Database: CINAHL (Ebscohost)

URL: ebscohost.com

Date Searched: 14.02.2024

| **#** | **Searches** | **Results** | **Column1** |
| --- | --- | --- | --- |
| 1 | (MH "Meningitis+") OR (TX meningiti*) OR TX ((meningococc*) N3 (infection* OR disease*)) | 14 720 |  |
| 3 | ((DE "Qualitative Measures" OR DE "Qualitative Methods" OR DE "Focus Group" OR DE "Grounded Theory" OR DE "Interpretative Phenomenological Analysis" OR DE "Narrative Analysis" OR DE "Semi-Structured Interview" OR DE "Thematic Analysis")) OR (TX (experiences OR qualitative OR interview* OR focus-group* OR semi-structured OR semistructured OR phenomenol* OR ethnograph* OR grounded-theory OR grounded-study OR groundeattituded-analysis OR grounded-analyses OR story OR stories OR content-analysis OR thematic-analysis OR thematic-analyses OR content-analys* OR narrative-analysis OR fieldwork OR field-work OR ((decriptive OR field) N3 (study)) OR key-informant* OR investigative OR theme* OR Thematic OR participant-observation* OR group-discussion*) OR (("semi-structured" or semistructured or unstructured or informal or "in-depth" or indepth or "face-to-face" or structured or guide*) N5 (discussion* or questionnaire* OR survey*))) | 993 911 |  |
| 4 | 1 and 2 | 1390 |  |
| 2 | (MH "Meningitis, Bacterial") OR (MM "Anthrax Meningitis") OR (MM "Meningitis, Listeria") OR (MM "Meningitis, Meningococcal") OR (MM "Meningitis, Pneumococcal") OR (MH "Meningitis, Viral") OR (MH "Meningitis, Fungal") OR TX ((Bacterial OR Viral OR Fungal OR Aseptic OR Parasitic OR community-acquired OR Acute OR fulminat* OR Fulminant OR Sudden-onset ) N5 (meningiti*)) OR TX (infectious-meningiti* OR Meningococc* OR Neisseria-meningit* OR N-Meningitidis OR Pneumococc* OR S-pneumoniae* OR Haemophilus-influenzae OR Listeri* OR L-monocytogenes OR Staphylococc* OR Staph-aureus OR Enterobacter* OR Enterococc* OR Escherichia-coli OR E-coli OR Streptococc* OR S-agalactiae* OR S-pyogenes OR Enterovir* OR Coxsackieviruses OR Herpesviridae OR Herpesvirus* OR herpes-virus* OR Varicella-zoster OR flavi-virus* OR Japanese-encephal* OR Tick-borne-encephal* OR Powassan-virus* OR West-Nile-virus OR Togaviridae OR Toga-virus* OR Togavir* OR equine-encephal* OR Bunyavirus* OR crosse-encephal* OR Toscana-virus* OR Reovirus* OR tick-fever* OR paramyxovir* OR Mumps OR morbillivirus* OR parainfluenza* OR Orthomyxovir* OR Influenza OR HIV OR human-immuno-deficienc* OR Adenoviridae OR adenovirus* OR Arenavir* OR Choriomeningit* OR LCMV OR Rickettsi* OR Orientia-spp OR Ehrlichia-spp OR spirochet* OR Borrelia-spp OR B-burgdorferi OR leptospir* OR Treponema-pallidum OR Brucell* OR Coxiella OR Mycoplasma OR spirillum* OR Naegleria OR angiostrongyl* OR Trichinella-spiralis* OR Candida OR Coccidioid* OR Histoplasm* OR Blastomyc* OR Sporothrix* OR Aspergill* OR Lyme OR Syphili* OR Scrub-Typhus OR tsutsugamushi) | 278 500 | NOT USED |

# Box 2. Reflexivity statement

| We acknowledge that subjectivity and context inherently influence the research process. To ensure qualitative research rigor, we employed a range of strategies aimed at addressing individual perspectives and minimising bias throughout the study.  Our research team responsible for data analysis and interpretation consisted of professionals from diverse backgrounds, including experienced (DM) and early-career quantitative researchers (MA, MP); medical practitioners at various stages of their careers, including psychiatrists (KK, ES, MS), and medical students (DB, MK, AM), all contributing to a multidisciplinary approach. Considering a stronger training of our team in quantitative methods, we consulted experienced qualitative researchers (MM, ADG, TJ, ZM, SN) with backgrounds in nursing, psychology, and social sciences at all stages of thematic synthesis, including selecting the data analysis method, coding, and theme identification and refinement. By utilising this multidisciplinary strategy, we aimed to balance any influence of our backgrounds and experiences on the findings, thereby ensuring they both meet high standards in qualitative research and hold practical utility for policymaking.  None of the authors involved in conducting the analysis had a direct experience working with individuals affected by meningitis, and all were from high-income countries. While this context could serve as a source of underestimation and underrepresentation of certain aspects of the data, we reasoned that a lack of prior experience would allow us to approach the analysis with minimal expectations or assumptions about the values, experiences and factors related to meningitis care. This background may have allowed for a more impartial interpretation of the data from primary studies and minimised potential bias in the study findings. Moreover, lack of prior assumptions influenced our decision to adopt a more flexible, exploratory methodology— thematic synthesis—ensuring that the results were driven by the data itself rather than pre-established frameworks. |
| --- |

##

## Table S1. Critical Appraisal Skills Programme (CASP) checklist for qualitative data

| N | Author, year | Was there a clear statement of the aims of the research? | Is a qualitative methodology appropriate? | Was the research design appropriate to address the aims of the research? | Was the recruitment strategy appropriate to the aims of the research? | Was the data collected in a way that addressed the research issue? | Has the relationship between researcher and participants been adequately considered? | Have ethical issues been taken into consideration? | Was the data analysis sufficiently rigorous? | Is there a clear statement of findings? | How valuable is the research? |
| --- | --- | --- | --- | --- | --- | --- | --- | --- | --- | --- | --- |
| 1 | Granier 1998 | Yes | Yes | No¹ | Yes | Yes | Yes | Can't tell | Yes | Yes | Valuable |
| 2 | Adedini 2021 | Yes | Yes | Yes | Yes | Yes | Can't tell | Yes | Yes | Yes | Valuable |
| 3 | Haines 2005 | Yes | Yes | Yes | Yes | Yes | Yes | Yes | Yes | Yes | Valuable |
| 4 | Kupst 1983 | Yes | Yes | Yes | Yes | Yes | Can't tell | Yes | Yes | Yes | Valuable |
| 5 | Jarvinen 2005 | Yes | No² | Yes | Yes | Yes | Can't tell | Can't tell | Can't tell | Yes | Valuable |
| 6 | Brennan 2003 | Yes | Yes | Yes | Yes | Yes | Can't tell | Yes | Yes | Yes | Valuable |
| 7 | Clark 2013 | Yes | Yes | Yes | Yes | Yes | Can't tell | Yes | Yes | Yes | Valuable |
| 8 | Neill 2022 | Yes | Yes | No³ | Yes | Yes | Can't tell | Yes | Yes | Yes | Valuable |
| 9 | Wisemantel 2018 | Yes | Yes | No⁴ | Yes | Yes | Can't tell | Yes | Can't tell | Yes | Valuable |
| 10 | Desmond 2013 | Yes | Yes | Can't tell | Yes | Yes | Can't tell | Yes | Yes | Yes | Valuable |
| 11 | Mahmoud 2022 | Yes | Yes | Yes | Yes | Yes | Can't tell | Yes | Yes | Yes | Valuable |
| 12 | Omoleke 2018 | Yes | Yes | Yes | Yes | Yes | Yes | Yes | Yes | Yes | Valuable |
| 13 | Sweeney 2013 | Yes | Yes | Can't tell | Yes | Yes | Yes | Yes | Yes | Yes | Valuable |
| 14 | Scanferla 2021 | Yes | Yes | No⁵ | Yes | Yes | Yes | Yes | Yes | Yes | Valuable |
| 15 | Scanferla 2020 | Yes | Yes | No⁶ | Yes | Yes | Yes | Yes | Yes | Yes | Valuable |
| 16 | Elafros 2022 | Yes | Yes | Yes | Yes | Yes | Can't tell | Yes | Yes | Yes | Valuable |
| 17 | Erickson 2001 | Yes | Can't tell | Yes | Yes | Yes | Can't tell | Can't tell | Yes | Yes | Valuable |
| 18 | Colombini 2009 | Yes | Yes | Yes | Yes | Yes | Can't tell | Yes | Can't tell | Yes | Valuable |
| 19 | Griffiths 2012 | Yes | Yes | Yes | Yes | Yes | Can't tell | Yes | Yes | Yes | Valuable |

1, 3-6: Recall bias could be introduced due to the time period between the meningitis case and participation in the study

2: The study results mostly quantitative. Qualitative data was not sufficiently described

## Table S2. Summary of Qualitative Findings and GRADE-CERQual Assessment

| **#** | **Summarised review finding** | **GRADE-CERQual Assessment of confidence** | **Explanation of GRADE-CERQual Assessment** | **References** |
| --- | --- | --- | --- | --- |
| **HOSPITALISATION – HICS** | | | | |
| 1 | Theme: Need for HCWs' greater awareness/alertness and rapid decision-making \|\| Patients and caregivers reported suboptimal knowledge about meningitis and IMD among healthcare workers, as evidenced by the fact that many patients received alternative initial diagnoses. The perceived lack of expertise evoked frustration and concern when healthcare workers were unable to respond to questions about the disease. Patients and carers also shared that, even in the face of obviously serious symptoms and poor condition, some doctors were slow to react, with some being passive and others panicking. On the other hand, families were satisfied with the provided medical care when doctors rapidly recognised the symptoms or initiated early treatment. | Low confidence | Serious concerns regarding methodological limitations: potential recall bias in four studies and lack of reflexivity in two studies, which was judged to potentially influence the finding. No/Very minor concerns regarding coherence. Minor concerns regarding adequacy: five studies offered moderately rich data, with less data available on the positive perceptions of doctors' performance. No/Very minor concerns regarding relevance | Scanferla et al. 2020; Neill et al. 2022; Wisemantel et al. 2018; Scanferla et al. 2021; Sweeney et al. 2013; |
| 2 | Theme: Importance of appropriate communication and information from HCWs \|\| Caregivers emphasised the importance of appropriate communication and a simple explanation from healthcare workers (HCWs). Insufficient communication sometimes caused frustration and prompted families to seek information independently. Carers noted that clear communication and more information about meningitis helped alleviate distress. In contrast, some expressed satisfaction with the information provided, particularly in written form, as well as with the support from the Population Health service. However, families also reported instances of disrespectful and dismissive communication, which contributed to their distress. Finally, parents felt that their concerns were unrecognised or underestimated by HCWs. | Moderate confidence | Serious concerns regarding methodological limitations: potential recall bias in four studies, no reflexivity statement in four studies, not sufficient information about data analysis in one study, No concerns regarding coherence, No concerns regarding adequacy, and Very minor concerns regarding relevance: while the finding may not be directly related to healthcare services, it offers information about experiences during hospitalisation in general. | Kupst et al. 1983; Scanferla et al. 2020; Neill et al. 2022; Wisemantel et al. 2018; Scanferla et al. 2021; Haines 2005; Clark et al. 2013; Sweeney et al. 2013; |
| 3 | Theme: Parental emotional turmoil during hospitalisation \|\| The period of hospitalisation was an overwhelmingly difficult emotional experience for parents. Given the serious nature of the disease, parents' primary concern revolved around the survival of their child, with many expressing profound worry about this outcome. After receiving the diagnosis of meningitis, parents were shocked, confused, and daunted. The diagnosis was described as unexpected, as many parents had never considered it could happen to their child. Although the majority of parents described the experience of hospitalisation as traumatising, some parents found hospitalisation a relief, once their child was in a controlled hospital environment and receiving medical attention and care. The experience of hospitalisation in an ICU added to the emotional burden of parents whose children had a particularly severe illness. Parents were distressed, anxious, and emotionally unprepared to see changes in their child's appearance and behaviour caused by support equipment and treatments. | Low confidence | Serious concerns regarding methodological limitations: potential recall bias in three studies, which were judged to influence the finding. Minor concerns regarding coherence: the finding is consistent with the supporting data, but leaves out some experience with a less negative sentiment. No/Very minor concerns regarding adequacy. Minor concerns regarding relevance: the finding does not reflect experiences with healthcare services but hospitalisation in general. | Kupst et al. 1983; Wisemantel et al. 2018; Scanferla et al. 2021; Haines 2005; Sweeney et al. 2013; |
| 4 | Theme: Coping strategies and emotional support during hospitalisation \|\| Caregivers reported diverse experiences regarding emotional support during hospitalisation. While some expressed a need for additional support, such as counselling services, and noted that the assistance from healthcare workers (HCWs) was inadequate, others were satisfied with the care provided in hospitals or felt that support from family and friends was sufficient, eliminating the need for further psychological assistance during their admission. Additionally, caregivers identified several factors that helped them manage stress, including support from family members, shared responsibilities with relatives and friends, interactions with other parents in the hospital, intervenors, religious beliefs, the attentiveness and hospitality of HCWs, the quality of medical care, prior experiences, and a positive attitude. | Low confidence | Serious concerns regarding methodological limitations: potential recall bias in three out of five studies, no reflexivity statement in two studies, insufficient information about data analysis in one study, No concerns regarding coherence, No concerns regarding adequacy, and Moderate concerns regarding relevance: the finding is not directly related to experience with the healthcare services. | Kupst et al. 1983; Wisemantel et al. 2018; Scanferla et al. 2021; Haines 2005; Sweeney et al. 2013; |
| **HOSPITALISATION – LMICS** | | | | |
| 5 | Theme: Perceptions of lumbar puncture outcomes \| Patients and caregivers perceived LP as a potentially fatal procedure associated with adverse outcomes. Most notably, the fear of death and paralysis emerges as a dominant concern. Death was attributed to delayed procedure uptake or a patient’s poor overall condition, along with concerns that the patient's position during or after the procedure could lead to paralysis. These perceptions were further fueled by second-hand experiences with adverse outcomes. However, patients and caregivers have observed improvements in the outcomes of LP in recent years, which they attributed to advances in procedural techniques and, in some instances, to divine intervention. | Moderate confidence | Very minor concerns regarding methodological limitations: no reflexivity in one study, No concerns regarding coherence, Moderate concerns regarding adequacy: one study with moderately rich data contributed to the finding, and Minor concerns regarding relevance: relevant data about the experience with LP, but only from one study. | Elafros et al. 2022; |
| 6 | Theme: Economic impact of medical treatment on families \| Orthodox treatment carried a great financial burden for families: medicaments and prescriptions were costly, requiring caregivers to sell their properties and incur substantial debts to afford treatment expenses. The lack of funds was the primary reason for seeking alternative types of treatment before going to the hospital. | Moderate confidence | Minor concerns regarding methodological limitations: there were concerns about reflexivity and potential recall bias in the single contributing study. Considering the sensitive nature of the finding, this limitation could have influenced participants' responses. No/Very minor concerns regarding coherence. Moderate concerns regarding adequacy: one study contributed to the finding, offering relatively thin data. No/Very minor concerns regarding relevance. | Griffiths et al. 2012; |
| 7 | Theme: Challenges in diagnosing meningitis \| Healthcare workers claimed knowledge of meningitis signs and symptoms but highlighted difficulties in diagnosing the disease. Meningitis was sometimes misdiagnosed as malaria, resulting in the prescription of anti-malarial medication. | Very low confidence | Serious concerns regarding methodological limitations: no reflexivity statement in one study, concerns about research design, No concerns regarding coherence, Serious concerns regarding adequacy: only one study with thin data, and No concerns regarding relevance | Desmond et al. 2013; |
| 8 | Theme: Miscommunication between HCWs and end-users \| Narratives from healthcare workers and patients revealed that it is common for patients to be verbally mistreated, which affects their decision to seek help at conventional healthcare facilities. Doctors stated that patients can be shouted at, disregarded, and blamed, specifically for failing to control the symptoms of meningitis. | Low confidence | Moderate concerns regarding methodological limitations: no reflexivity and potential recall bias. No concerns regarding coherence. Serious concerns regarding adequacy: only one study with relatively thin data. No concerns regarding relevance | Desmond et al. 2013; |
| 9 | Theme: Fear of complications as a barrier to LP uptake \| Patients and caregivers expressed fear about potential complications related to lumbar punctures (LP), specifically death and paralysis. These concerns were shaped by previous negative experiences with the procedure and the historical association of LP with high mortality rates during the HIV epidemic. | Low confidence | Moderate concerns regarding methodological limitations: no reflexivity statement in one study, No concerns regarding coherence, Moderate concerns regarding adequacy: only one study with relatively thin but descriptive data, and No concerns regarding relevance | Elafros et al. 2022; |
| 10 | Theme: Reliance on shared decision-making \| The decision to consent to a medical procedure was influenced by the shared nature of decision-making. Caregivers and patients consulted older family members when deciding to consent to LP, in some cases, to share responsibility and avoid being blamed. Even when the patient was sufficiently well to make their own medical decisions, family consensus may have overruled patient wishes, especially if too much time passes between consent and procedure completion. | Moderate confidence | Very minor concerns regarding methodological limitations: no reflexivity, No concerns regarding coherence, Moderate concerns regarding adequacy: one study with moderately rich data, and No concerns regarding relevance | Elafros et al. 2022; |
| 11 | Theme: Patients' values driving LP consent \| Patients identified several factors influencing their decision to consent to LP. Trust in physicians and confidence in their technical abilities facilitated the acceptance of the procedure. The desire for diagnostic clarity and appropriate treatment also served as a justification for LP, with some viewing the procedure as a way to potentially reduce the length of hospital stays. Concern over the patient’s health and disease progression further motivated families to agree, typically later in the illness course. | Low confidence | Moderate concerns regarding methodological limitations: no reflexivity statement. No concerns regarding coherence. Moderate concerns regarding adequacy: one study with moderately rich, but descriptive data. No concerns regarding relevance | Elafros et al. 2022; |
| 12 | Theme: Systemic and operational barriers in healthcare organisation. Reports amongst healthcare workers highlighted several issues related to the organisation of healthcare services. These include poor hospital logistics, lack of sterility, risk of iatrogenic infections, time constraints, and the requirement for a CT scan prior to performing a lumbar puncture. Additionally, some healthcare workers addressed the lack of expertise and knowledge about contraindications of lumbar puncture among doctors, with some citing it as a reason they would be hesitant to undergo or perform the procedure. Drug supply shortages were also noted in both epidemic and non-epidemic settings. In epidemic setting, it was reported that healthcare workers were often unaware of official guidelines regarding different payment schedules, which led to increased out-of-pocket expenses for medications, despite official government policy. | Moderate confidence | Moderate concerns regarding methodological limitations: no reflexivity, concerns about data analysis and potential recall bias, No concerns regarding coherence, Minor concerns regarding adequacy: one study with moderately rich data, two studies with thin data, and No concerns regarding relevance | Elafros et al. 2022; Colombini et al. 2009; Desmond et al. 2013; |
| 13 | Theme: Community apprehensions influencing HCW's decision making. Healthcare workers (HCWs) reported that community apprehensions influenced their decision to refer patients for LP. HCWs were reluctant to perform LPs on terminally ill patients due to concerns that if the patient died shortly after, others might perceive the procedure as the cause of death. While caregivers were often more willing to consent to LPs later in the admission, HCWs believed that at this stage, the procedure would no longer have a meaningful impact on patient outcomes. | Low confidence | Moderate concerns regarding methodological limitations: no reflexivity statement, which might have influenced HCWs' replies, No concerns regarding coherence, Moderate concerns regarding adequacy: only one study with thin data, and No concerns regarding relevance | Elafros et al. 2022; |
| 14 | Theme: Consent practices facilitating LP delivery \| Firstly, consent was provided only verbally, as it was the norm for LP in this area. By omitting written consent, healthcare workers believed to prevent patients from having misconceptions about the procedure. While consent was obtained only verbally, the LP refusal was formally documented in medical records. Secondly, healthcare workers prioritised patient care over the consent process to save time. While some entirely skipped the consent process, others modified it to obtain consent more rapidly: they recalled manipulating risks (minimising or omitting) of LP during the consent process to reduce the probability of LP refusal. Finally, healthcare workers believed that by clearly explaining the purpose of LP and effectively communicating with patients, they were more likely to obtain consent. | Moderate confidence | Moderate concerns regarding methodological limitations: as the finding reflects consent practices reported by healthcare workers themselves, lack of reflexivity was judged to potentially influence the finding. No/Very minor concerns regarding coherence. Minor concerns regarding adequacy: one study contributed to the finding, offering moderately rich and sufficiently large in quantity data. No/Very minor concerns regarding relevance. | Elafros et al. 2022; |
| **POST-HOSPITALISATION – HICS** | | | | |
| 15 | Theme: Multifaceted impact of meningitis on physical, mental, and social well-being \|\| Meningitis survivors and their carers reported disabling physical and mental health sequelae of meningitis, causing daily frustration and limitations in social activities. Among some of the cited sequelae were limb loss, hearing and vision impairment, paralysis, depression, memory impairment in adults, and developmental delay in children. Additionaly, meningitis survivors highlighted the impact of these sequelae on their social and personal activities and life perspectives. Some patients had to change career, education and family plans, while others reported social isolation and changes in personal motivation. Rehabilitation was an additional source of daily psychological distress, especially when patients were involved in long periods of rehabilitation. | Low confidence | Moderate concerns regarding methodological limitations: potential recall bias in two studies, which is judged to have minimal impact on the finding. Concerns about study design, reflexivity, and ethical approval in one study, which raised concerns about the quality of the provided qualitative data. Minor concerns regarding coherence: generally coherent, but mostly reflects major patterns in the underlying data. No/Very minor concerns regarding adequacy. Serious concerns regarding relevance: indirect relevance; does not reflect experience with healthcare services but meningitis sequelae in general. | Scanferla et al. 2020; Scanferla et al. 2021; Erickson et al. 2001; |
| 16 | Theme: Long-term psychological impact of hospitalisation \|\| Some patients had traumatic experiences and developed phobias related to medical procedures, personnel or hospital environment following their hospitalisation. In some cases, these fears persisted long after their recovery. The psychological impact of meningitis hospitalisation also manifested in distress, feelings of helplessness and depression among both survivors and their caregivers. | Moderate confidence | Moderate concerns regarding methodological limitations: potential recall bias in four studies, no reflexivity statement in two studies, insufficient information about data analysis in one study, Minor concerns regarding coherence: the finding lacks specific descriptions of psychological changes, Very minor concerns regarding adequacy: the overall richness of data was considered to be moderate, and No/Very minor concerns regarding relevance | Kupst et al. 1983; Scanferla et al. 2020; Wisemantel et al. 2018; Scanferla et al. 2021; Haines 2005; Sweeney et al. 2013; |
| 17 | Theme: Parental concerns about potential consequences of meningitis \|\| Caregivers of children who had survived meningitis were concerned about potential long-term consequences of the illness. Some parents questioned if their child's health, development, and behaviour were normal or if any abnormalities could be treated as after-effects of meningitis. Others were anxious about child's future and well-being in general. | Low confidence | Minor concerns regarding methodological limitations: potential recall bias in three studies, which was judged to minimally influence the finding. Insufficient data on reflexivity in two studies and on data analysis in one. These limitations were judged to unlikely influence the finding. No/Very minor concerns regarding coherence, Moderate concerns regarding adequacy: all four studies offered thin data, but one of them provided data from a large sample (n=244). Moderate concerns regarding relevance: the finding mostly reflects general experience with caregiving and not healthcare services. | Kupst et al. 1983; Wisemantel et al. 2018; Scanferla et al. 2021; Sweeney et al. 2013; |
| 18 | Theme: Need for care continuity, education and support \|\| Caregivers emphasized the need for prolonged care, support and education regarding meningitis sequelae. Parents wanted reassurance from healthcare workers about their child's medical condition and highlighted the importance of follow-up appointments, additional medical tests, and specialist assessments to evaluate potential sequelae and identify any special needs. Furthermore, some caregivers reported a lack of knowledge about meningitis sequelae and expressed a desire for more information. Additionally, parents noted feeling overwhelmed and distressed following the diagnosis and indicated a need for psychological support after their child's discharge. | Low confidence | Moderate concerns regarding methodological limitations: potential recall bias in three studies, no reflexivity statement in two studies, insufficient information about data analysis in one study, No concerns regarding coherence, Very minor concerns regarding adequacy, and Moderate concerns regarding relevance: the finding is not directly related to the values and experiences with healthcare services, but it offers information about experiences with meningitis sequelae in general. | Wisemantel et al. 2018; Scanferla et al. 2021; Haines 2005; Clark et al. 2013; Sweeney et al. 2013; |
| 19 | Theme: Perceptions of quality of aftercare for meningitis sequelae \|\| Parents of children with meningitis sequelae had contrasting experiences with aftercare services. Some parents were unhappy with the provided care and reported inadequate customisation of prosthetic limbs and orthopaedic devices. Carers also noted poor communication between different members of the aftercare process and different anticipated goals of rehabilitation, which delayed timely and sufficient care. On the other hand, when parents were satisfied with the provided rehabilitation services, aftercare was tailored and suitable for their child’s needs. Effective communication and listening to parents' expectations of the process were seen to play a crucial role in good care. | Moderate confidence | Moderate concerns regarding methodological limitations: no data to judge if reflexivity was adequate, which could have influenced the finding. No/Very minor concerns regarding coherence, Minor concerns regarding adequacy: one study offered rich data, which was considered adequate for a descriptive finding. No/Very minor concerns regarding relevance | Clark et al. 2013; |
| 20 | Theme: Lack of appreciation for less apparent sequelae of meningitis \|\| A lack of recognition and understanding of the less visible psychosocial and cognitive after-effects of meningitis hindered parental ability to access support services, particularly in educational settings. Young age acted as an additional barrier to gaining access to aftercare because of difficulty testing young children, misconceptions about the needs of disabled children and challenges in predicting cognitive after-effects at the time of discharge. | Moderate confidence | Very minor concerns regarding methodological limitations: no reflexivity statement in one study, No concerns regarding coherence, Moderate concerns regarding adequacy: only one study with relatively thin data, and Minor concerns regarding relevance: while the finding does not directly describe the experiences with healthcare services, it provides information about potential barriers to gaining access for patients with meningitis sequelae. | Clark et al. 2013; |
| 21 | Theme: Systemic and organisational barriers to aftercare \|\| Parents of childhood meningitis survivors reported difficiulties with accessing and navigating aftercare services, including disability living allowance and social care, and expressed a need for support. Carers cited factors such as lack of staff, lack of communication between different members of the process, restricted budget, and complex bureaucratic procedures as barriers to timely, sufficient, and tailored rehabilitation. Additionaly, they emphasised on the limited inclusion criteria, which posed significant barriers for young children and those with cognitive or psychological sequelae. Impairments in these children was sometimes borderline and less apparent, and aftercare services failed to recognise the link between meningitis and such non-physical sequelae, further complicating access to rehabilitation. | High confidence | Minor concerns regarding methodological limitations: potential recall bias in two of the three studies, no reflexivity in one. These limitations would unlikely influence the finding, given its' more generic scope. No/Very minor concerns regarding coherence. Minor concerns regarding adequacy: one study offered rich data, and two offered thin data. The data were judged as adequate, as the finding is descriptive. No/Very minor concerns regarding relevance | Scanferla et al. 2021; Clark et al. 2013; Sweeney et al. 2013; |
| 22 | Theme: Third parties as facilitators of meningitis sequelae aftercare \|\| Parents highly valued tailored care suitable for their child’s needs. The school was seen as having a special role to play in providing accessible, long-term and timely follow-up care. Additionally, parents reported the active involvement of a consultant and multidisciplinary team meetings including parents, school staff, and health visitors as factors helping overcome difficulties in accessing aftercare. | Moderate confidence | Very minor concerns regarding methodological limitations: no reflexivity statement in one study, No concerns regarding coherence, Moderate concerns regarding adequacy: only one study with relatively thin data, and Minor concerns regarding relevance: while the finding does not directly describe the experiences with healthcare services, it provides information about potential facilitators to gaining access for patients with meningitis sequelae. | Clark et al. 2013; |
| **PRE-HOSPITALISATION – HICS** | | | | |
| 23 | Theme: Need for awareness and comprehensive knowledge about meningitis \|\| Patients and caregivers had little or no prior knowledge about meningitis etiology and symptoms before the diagnosis. Once meningitis was diagnosed, people expressed a strong desire for more information about the disease, often approaching the internet or contacting associations to fill in the gaps. Parents and carers were highlighting the need for better public education and awareness campains to recognise the signs of meningitis. | Low confidence | Very minor concerns regarding methodological limitations: concerns about reflexivity, data analysis and recall bias, Very minor concerns regarding coherence, No concerns regarding adequacy, and Serious concerns regarding relevance: indirect relevance; does not reflect experience with healthcare services but meningitis in general. | Scanferla et al. 2020; Wisemantel et al. 2018; Scanferla et al. 2021; Sweeney et al. 2013; |
| 24 | Parental emotional reactions during initial stages of meningitis \|\| Prior to hospitalisation, parents experienced complex emotions ranging from anger and disbelief to fear and a sense of loss of control. Parents revealed they experienced immense fear in front if a serious disease, which sometimes lead to denial of the illness at all. The fear was exacerbated by the sense of helplessness and loss of control over child's condition. Feelings of anger and disbelief came over when parents failed to find the expected confirmation of their concerns and appropriate support during first contact with healthcare services. Transportation to the healthcare facility was another source of emotional burden during the initial staged of the illness. Parents were stressed when they were excluded from accompanying their child, but understanding the rationale behind it and involvement of a team of health specialists helped to ease the stress. | Low confidence | Moderate concerns regarding methodological limitations: potential recall bias in one study could affect the finding. No/Very minor concerns regarding coherence. Minor concerns regarding adequacy: three studies together offered moderately rich data, which was considered sufficient, given the finding is descriptive. Moderate concerns regarding relevance: the finding provides little data on experiences of caregivers with healthcare services before hospitalisation. | Kupst et al. 1983; Neill et al. 2022; Haines 2005; |
| 25 | Theme: Parental intuition and recognition of illness \|\| Intuition stimulated parents to seek medical attention, even when the symptoms did not immediately suggest meningitis. They recognised the overall deterioration of children's health based on subtle changes in their behaviour or physical condition. | Moderate confidence | Moderate concerns regarding methodological limitations: concerns about recall bias, reflexivity, and data analysis, Very minor concerns regarding coherence: in one study, it is not explicitly stated that intuition stimulated help-seeking, Minor concerns regarding adequacy: moderately rich data, however, the finding is rather descriptive, and Minor concerns regarding relevance: the finding is indirectly related to the uptake of healthcare services. | Neill et al. 2022; Wisemantel et al. 2018; Haines 2005; Brennan et al. 2003; |
| 26 | Theme: Many masks of meningitis clinical presentation \|\| General practitioners reported having limited experience in diagnosing meningitis and meningococcal disease, which can present with diverse clinical manifestations and pose diagnostic challenges. Symptoms such as a non-blanching purpuric rash, neck pain, and rapid disease progression increased the certainty of a meningitis diagnosis. Conversely, non-specific symptoms were often less apparent to healthcare workers and could mislead the diagnostic process. Despite the uncertainty in the diagnosis of meningitis, doctors acknowledged that atypical clinical presentations might indicate a more serious underlying illness, prompting them to hospitalise such patients. Additional symptoms that led to the admission of these complex cases included lethargy, decreased mobility, altered consciousness and mental state, pallor, cyanosis, and abnormal crying. | Low confidence | Serious concerns regarding methodological limitations: potential recall bias in one study and lack of reflexivity in the other study, which could potentially influence the finding. Minor concerns regarding coherence: the finding does not reflect some minor opposing data but provides data on major trends in medical practice. No/Very minor concerns regarding adequacy. No/Very minor concerns regarding relevance | Brennan et al. 2003; Granier et al. undefined; |
| 27 | Theme: Role of context and parental input in clinical decision-making \|\| General practitioners (GPs) noted that parental anxiety significantly influenced their clinical decision-making, particularly when they had an established relationship with the family. Some parents expressed fear of meningitis that, according to healthcare workers, emerged as a result of the prevalence of awareness campaigns. GPs recognised that while these fears were sometimes disproportionate, awareness campaigns are still needed due to the severity of meningitis. Parental concerns sometimes served as facilitators for further clinical evaluation, with some parents directly prompting GPs to reconsider their initial assessments. | Moderate confidence | Moderate concerns regarding methodological limitations: concerns about reflexivity, recall bias, and ethics, Minor concerns regarding coherence: several parts of the finding are supported only by one citation, No concerns regarding adequacy, and No concerns regarding relevance | Brennan et al. 2003; Granier et al. undefined; |
| 28 | Theme: Intuitive and evidence-based practice \|\| General practitioners (GPs) revealed that in general practice they rely much more on experience and intuition rather than evidence and logic. GPs acknowledged the utility of guidelines, but expressed skepticism about their application and noted challenges in keeping up with updates. They stressed that guidelines can undermine individualised and personal patient care and interfere with the more intuitive approach to diagnosis, which was deemed more helpful in case of an unusual clinical presentation. Moreover, GPs stated their priority was to identify a serious illness - where intuition was a key factor - rather than to make a definitive diagnosis . When identifying  a serious illness, general practitioners often recognised the overall poor condition, changes in usual behaviour of patients and 'puzzling' findings, rather than specific signs and symptoms. | Low confidence | Serious concerns regarding methodological limitations: no reflexivity in two studies and potential recall bias in one, which could significantly influence the finding. Concerns about qualitative data collection and analysis in one study. No/Very minor concerns regarding coherence. Minor concerns regarding adequacy: one study offered rich and comprehensive data, while the other two offered thin data. No/Very minor concerns regarding relevance | Jarvinen et al. 2005; Brennan et al. 2003; Granier et al. undefined; |
| 29 | Theme: Sociocultural factors influencing help-seeking behaviour \|\| Caregivers expressed hesitance in seeking assistance due to concerns about the potential misuse or overuse of healthcare resources, particularly when uncertain about the severity of the illness. This reluctance to overutilise the healthcare system, along with other parental responsibilities, ultimately delayed their decision to seek medical attention. | Low confidence | Moderate concerns regarding methodological limitations: potential recall bias, no reflexivity, No concerns regarding coherence, Serious concerns regarding adequacy: one study offering thin data, and Minor concerns regarding relevance: the finding is indirectly related to the uptake of healthcare services. | Neill et al. 2022; |
| 30 | Theme: Systemic and operational barriers in healthcare organisation \|\| Healthcare workers in primary care settings reported not being confident with lack experience in treating meningitis, thus, they were more focused on getting the child hospitalized as early as possible rather than starting treatment on their own. The primary source of concern was lack of experience with administering parenteral antibiotics and potential difficulties with intravenous access. Additionally, GPs revealed that treatment could have been delayed due to advice or disapproval from clinical or perscription consultants and lack of immediate access to antibiotics. | Very low confidence | Serious concerns regarding methodological limitations: no reflexivity in two studies, which could significantly influence the finding. Concerns about qualitative data collection and analysis in one study. No/Very minor concerns regarding coherence. Serious concerns regarding adequacy: two studies offered thin data, one of which did not provide any details behind the data. No/Very minor concerns regarding relevance | Jarvinen et al. 2005; Brennan et al. 2003; |
| 31 | Theme: Factors influencing pre-hospital antibiotic treatment initiation \|\| General practitioners (GPs) were more likely to administer antibiotics pre-hospital when they were confident in their diagnosis. In cases with less certainty, the presence of severe symptoms sometimes stimulated GPs to take action. However, some GPs were hesitant to initiate treatment without definitive signs, preferring to wait until the diagnosis was clear. The presence of a non-blanching rash was identified as one of the most reliable indicators that led to the initiation of antibiotic treatment. | Low confidence | Moderate concerns regarding methodological limitations: in one study, a potential recall bias could be introduced, No concerns regarding coherence, Moderate concerns regarding adequacy: only two studies contributed to the finding offering relatively thin data, and No concerns regarding relevance | Brennan et al. 2003; Granier et al. undefined; |
| **PRE-HOSPITALISATION – LMICS** | | | | |
| 32 | Theme: Knowledge and perceptions of meningitis \| Community members perceived meningitis as a dangerous disease typically presenting with stiff neck and seizures. Participants acknowledged that meningitis can result in death and disability, which was particularly scared of in children due to the potential loss of productivity and income in the future. Despite previous educational efforts, community's modern knowledge about the causes of meningitis was limited and centered around spiritual or supernatural influence. Some participants additionally referred to direct contact with an ill person, specific weather conditions, and foods that were associated with meningitis. | Low confidence | Very minor concerns regarding methodological limitations. Moderate concerns regarding coherence: the finding captures only the most dominant patterns, while the data were more varied. Very minor concerns regarding adequacy. Serious concerns regarding relevance: the finding reflects experiences with meningitis in general, not with healthcare services. | Colombini et al. 2009; Desmond et al. 2013; Adedini et al. 2021; Mahmoud et al. 2022; |
| 33 | Theme: Conflict and convergence between biomedical and traditional treatment \| Many caregivers and patients showed a preference for biomedical treatment in managing meningitis, particularly after realizing the limitations of traditional healing methods. It was emphasised that the hospital was the preferred option for treatment due to doctors' expertise despite maintained spiritual beliefs about the disease's origins. However, other end-users still favoured traditional medicine. Some of them expressed doubt about the medical diagnosis of meningitis, attributing their illness to curses, dreams, or old age. | Low confidence | Moderate concerns regarding methodological limitations: no reflexivity statement in two studies; Minor concerns regarding coherence: one of two studies does not provide information about the values of patients, while the other one offers data from both patients and caregivers; Moderate concerns regarding adequacy: one study offers rich data, while the other provides only thin data, and No/Very minor concerns regarding relevance: one study provides only thin data about end users' values on meningitis treatment. | Adedini et al. 2021; Mahmoud et al. 2022; |
| 34 | Theme: Sociocultural factors influencing health-seeking behaviour \| Health-seeking behaviour was largely influenced by the sociocultural norms established in the community. In a hierarchical society, patients and caregivers, especially women, usually sought validation of disease severity from senior and often male family or community members. In contrast to men, many women had limited or no formal education and were unemployed, which constrained their capacity to make independent decisions. Confirmation of disease severity was essential to warrant funding. However, it was commonly recognised only when the disease interfered with a patient's social activity, delaying timely care. Treatment preferences were also driven by the widespread perception among patients, carers of adult and paediatric patients, and community members that meningitis, in case of supernatural causes, should be treated with Islamic or traditional methods. Families consulted traditional healers despite acknowledging the effectiveness of conventional medicine and its availability, particularly to discern if the disease has supernatural origins. | Moderate confidence | Minor concerns regarding methodological limitations: lack of reflexivity in four studies, which could influence the finding. Moderate concerns regarding coherence: the finding captures only the most dominant patterns, leaving out contrasting patterns present in the 'gender inequalities' aspect of the finding. No/Very minor concerns regarding adequacy. No concerns regarding relevance. | Colombini et al. 2009; Omoleke et al. 2018; Desmond et al. 2013; Adedini et al. 2021; Mahmoud et al. 2022; |
| 35 | Theme: Initial response regarding preferred treatment \| The initial response to disease signs in caregivers and patients involved self-medication and alternative treatment. Several families reported administering medications such as paracetamol to alleviate fever or headaches during the early stages of the illness. Caregivers also frequently mentioned favouring alternative medicine, including the help of prayers, traditional healers and soothsayers, to orthodox care. This preference was associated with the prevailing reliance on supernatural explanations for the illness and was particularly evident among older patients and caregiver groups who belonged to rural communities. Some caregivers also clarified that the reasons for favouring alternative medicine were the shorter waiting period, lower cost and less severe illness. Patronage of government hospitals was considered the last resort when the illness became severe and not amenable to alternative care. | Moderate confidence | Moderate concerns regarding methodological limitations: no reflexivity in three out of four studies, one study lacks information about data analysis, and in one study, it is unclear whether the research design was appropriate to address aims; Minor concerns regarding coherence: three studies reported both alternative medicine and self-medication as an initial response, while the other one – only self-medication, Very minor concerns regarding adequacy. No concerns regarding relevance. | Colombini et al. 2009; Omoleke et al. 2018; Desmond et al. 2013; Adedini et al. 2021; |
| 36 | Theme: Lack of awareness and alertness to meningitis symptoms delays timely care \| Community members, patients and caregivers often underestimated meningitis symptoms, attributing them to more familiar causes, such as malaria or traditional illnesses, that were usually treated at home. Some non-specific symptoms, including severe headache, body weakness and loss of appetite, were not considered a real illness, which was associated with the delay in timely help-seeking. | High confidence | No/Very minor concerns regarding methodological limitations. No/Very minor concerns regarding coherence. Minor concerns regarding adequacy: one study offered moderately rich data, and two offered thin data. Data were considered adequate (minor concerns) for a descriptive finding. No/Very minor concerns regarding relevance. | Griffiths et al. 2012; Desmond et al. 2013; Mahmoud et al. 2022; |
| 37 | Theme: Financial barriers to healthcare \| Patients and caregivers delayed seeking treatment due to the financial burden associated with healthcare. These financial constraints included not only direct costs of medical services but also transportation to the health facilities. Some families reported having to borrow money to cover healthcare expenses. In some cases, these financial limitations stimulated them to seek alternative medicine before pursuing hospital care. | Moderate confidence | Very minor concerns regarding methodological limitations: no reflexivity statement in two studies, No concerns regarding coherence, Minor concerns regarding adequacy: one study with rich data, two studies with thin data, and Minor concerns regarding relevance: one study only focused on experiences of caregivers. | Griffiths et al. 2012; Omoleke et al. 2018; Desmond et al. 2013; |
| 38 | Theme: Impact of perceived health service quality on health-seeking behaviour \| Both patients and healthcare workers reported poor organisation of healthcare services with long waiting times, presumptive diagnosis without examination, verbal mistreatment, and lack of follow-up guidance. The perceived suboptimal quality of care prompted patients to avoid hospitals and seek medical advice from alternative service providers. | Low confidence | Moderate concerns regarding methodological limitations: no reflexivity statement in one contributing study, which could have potentially influenced the finding. No/Very minor concerns regarding coherence. Moderate concerns regarding adequacy: two studies offered thin data. No/Very minor concerns regarding relevance. | Omoleke et al. 2018; Desmond et al. 2013; |
| 39 | Theme: Lack of early recognition \| Caregivers reported not recognising the early symptoms of meningitis and only seeking help in healthcare facilities when the disease has progressed. Prior experience with meningitis helped raise suspicion earlier. | Very low confidence | No/Very minor concerns regarding methodological limitations. Minor concerns regarding coherence: one study supported all aspects of the finding while the other only one part of the finding. Serious concerns regarding adequacy: overall richness and quantity of data are relatively low. Minor concerns regarding relevance: the finding provides information about one barrier to healthcare services uptake. | Desmond et al. 2013; Adedini et al. 2021; |
| 40 | Theme: Disease severity initiates help-seeking behaviour \| The key factor that encouraged caregivers to seek help at conventional healthcare facilities was recognition of disease severity rather than recognition of specific signs and symptoms of meningitis. Indicators of severity, such as social life disruption, severe weakness, loss of appetite, and the inability to work, were among the cited reasons driving individuals to seek help. | Moderate confidence | No/Very minor concerns regarding methodological limitations. Moderate concerns regarding coherence: one study reports cases when help-seeking at conventional healthcare facilities was initiated following failed attempts to control the disease with alternative medicine. However, the finding does not cover such cases. Moderate concerns regarding adequacy: one study on which this finding mostly relies offered moderately rich data. One of the contributing studies offered thin data. No/Very minor concerns regarding relevance. | Omoleke et al. 2018; Desmond et al. 2013; |
| **SEQUELAE – LMICS** | | | | |
| 41 | Theme: Multifaceted impact of meningitis on physical, mental, and social well-being \| Patients emphasised the long-term effects of meningitis on their physical, mental, and social well-being. Older patients and caregivers of children reported a range of complications, including cardiovascular problems, paralysis, hearing and vision impairments, cognitive decline and psychological changes. Meningitis sequelae significantly disrupted social activities and reduced the level of independence. | Very low confidence | No/Very minor concerns regarding methodological limitations: no reflexivity in two studies, Moderate concerns regarding coherence: the finding leaves out several reported aspects, Minor concerns regarding adequacy: one study offered relatively thin data and the other one – moderately rich data, and Serious concerns regarding relevance: the finding reflects experiences with meningitis sequelae in general, not with healthcare services. | Griffiths et al. 2012; Mahmoud et al. 2022; |
| 42 | Theme: Experiences with providing care for meningitis sequelae \| Aftercare for family members with meningitis sequelae was associated with some practical and psychological challenges. Carers reported the necessity to balance work comittments and caregiving responsibilities. Those caregivers who continued to work struggled to provide consistent and sufficient care, while others had to abandon their jobs to committ to care for their loved ones. Additionally, a single caregiver reported hiring a specialised perconnel to look after the child with sequelae, which posed a financial strain on the family. Psychologically, caring for older parents was perceived as a rewarding experience by some participants, but as a stressful experience by others. Psychological stress was induced by the feelings of isolation, frustration with taking care of older family members, and fear of economic instability. | Low confidence | No/Very minor concerns regarding methodological limitations. No/Very minor concerns regarding coherence. Moderate concerns regarding adequacy: of the two contributing studies, one offered moderately rich data and one offered thin data. Psychological burden of caregiving was reflected only in one study and supported by relatively thin data. Serious concerns regarding relevance: the finding reflects the general experiences with caregiving and not experiences with rehabilitation services. | Griffiths et al. 2012; Mahmoud et al. 2022; |
| 43 | Theme: Balancing marital and domestic responsibilities and caregiving \| Female caregivers faced challenges in balancing marital and domestic responsibilities while caring for ailing parents. Some women noted that marriage distanced them from their parental homes, complicating their ability for caregiving. In contrast, men were reported to have greater availability to provide care for relatives with meningitis sequelae. | Very low confidence | Very minor concerns regarding methodological limitations: no reflexivity, No concerns regarding coherence, Serious concerns regarding adequacy: one study with thin data, and Serious concerns regarding relevance: the finding reflects experiences with meningitis sequelae in general, not with healthcare services. | Mahmoud et al. 2022; |
| 44 | Theme: Preference for home care over institutionalization \| Most caregivers were sceptic about the benefits of professional aftercare services and feared that institutional care could further deteriorate their loved ones' health. This led to a belief that care should ideally be managed at home within the family. | Moderate confidence | Moderate concerns regarding methodological limitations: concerns regarding reflexivity and potential recall bias, which could have affected the finding. No/Very minor concerns regarding coherence. Moderate concerns regarding adequacy: one study contributed to the finding, offering relatively thin data. No/Very minor concerns regarding relevance. | Mahmoud et al. 2022; |
| 45 | Theme: Financial burden as a barrier to aftercare services uptake \| The financial aspects were a barrier to accessing aftercare services for children and adults who have experienced meningitis. Caregivers reported that the high costs associated with transportation, hospital consultations, and medical devices, such as hearing aids, contributed to the discontinuation of aftercare. | Moderate confidence | Very minor concerns regarding methodological limitations: no reflexivity, No concerns regarding coherence, Moderate concerns regarding adequacy: one study with relatively thin data, and No concerns regarding relevance | Griffiths et al. 2012; |

## Table S4. Evidence Profile Table

| # | Summarised review finding | Methodological limitations | Coherence | Adequacy | Relevance | GRADE-CERQual assessment of confidence | References |
| --- | --- | --- | --- | --- | --- | --- | --- |
| HOSPITALISATION – HICS | | | | | | | |
| 1 | Theme: Need for HCWs' greater awareness/alertness and rapid decision-making \|\| Patients and caregivers reported suboptimal knowledge about meningitis and IMD among healthcare workers, as evidenced by the fact that many patients received alternative initial diagnoses. The perceived lack of expertise evoked frustration and concern when healthcare workers were unable to respond to questions about the disease. Patients and carers also shared that, even in the face of obviously serious symptoms and poor condition, some doctors were slow to react, with some being passive and others panicking. On the other hand, families were satisfied with the provided medical care when doctors rapidly recognised the symptoms or initiated early treatment. | Serious concerns  **Explanation:** Serious concerns regarding methodological limitations. In four of the five studies that contributed to the finding, recall bias could be introduced due to the time between the acute episode of meningitis and the interview. In one study this period was not reported. The two studies did not report the relationship between researcher and participants, one of which also did not provide sufficient details on data analysis. Overall, these methodological limitations were judged to potentially influence the finding. As the finding reflects experiences during hospitalisation and attitudes towards healthcare workers, recall bias and lack of reflexivity could be critical in this case. | No/Very minor concerns  **Explanation:** No concerns regarding coherence. The finding reflects all views on healthcare workers' expertise and clinical behaviour that were shared in the contributing studies. | Minor concerns  **Explanation:** Minor concerns regarding adequacy. Five studies contributed to the finding, together offering moderately rich data. There was less supporting data on perceptions of satisfactory responses from doctors. However, considering the finding is descriptive, we concluded we have minor concerns regarding adequacy. | No/Very minor concerns  **Explanation:** Direct relevance. No concerns regarding relevance. The finding gives insights into caregivers' experiences with healthcare services, answering the review question. | Low confidence  **Explanation:** Serious concerns regarding methodological limitations: potential recall bias in four studies and lack of reflexivity in two studies, which was judged to potentially influence the finding. No/Very minor concerns regarding coherence. Minor concerns regarding adequacy: five studies offered moderately rich data, with less data available on the positive perceptions of doctors' performance. No/Very minor concerns regarding relevance | Neill et al. 2022; Scanferla et al. 2020; Scanferla et al. 2021; Sweeney et al. 2013; Wisemantel et al. 2018; |
| 2 | Theme: Importance of appropriate communication and information from HCWs \|\| Caregivers emphasised the importance of appropriate communication and a simple explanation from healthcare workers (HCWs). Insufficient communication sometimes caused frustration and prompted families to seek information independently. Carers noted that clear communication and more information about meningitis helped alleviate distress. In contrast, some expressed satisfaction with the information provided, particularly in written form, as well as with the support from the Population Health service. However, families also reported instances of disrespectful and dismissive communication, which contributed to their distress. Finally, parents felt that their concerns were unrecognised or underestimated by HCWs. | Serious concerns  **Explanation:** Serious concerns regarding methodological limitations. In four of the eight studies that contributed to the finding, recall bias could be introduced due to the time between the acute episode of meningitis and the interview. In one study, this period was not reported. The four studies did not report the relationship between the researcher and participants, one of which also did not provide sufficient details on data analysis. Overall, these methodological limitations were judged to potentially influence the finding. As the finding reflects experiences during hospitalisation and attitudes towards healthcare workers, recall bias and lack of reflexivity could be critical in this case. | No/Very minor concerns  **Explanation:** No concerns regarding coherence. The finding completely reflects the range of the underlying data. | No/Very minor concerns  **Explanation:** No concerns regarding adequacy. Eight studies contributing to the finding together offered very rich and diverse data. While three studies provided relatively thin data, the remaining five offered detailed and comprehensive information. | No/Very minor concerns  **Explanation:** Very minor concerns regarding relevance. The finding is primarily focused on caregivers' experiences with communication with healthcare workers. While it may not be directly tied to healthcare services, it nonetheless offers valuable information about experiences during hospitalisation more broadly. | Moderate confidence  **Explanation:** Serious concerns regarding methodological limitations: potential recall bias in four studies, no reflexivity statement in four studies, not sufficient information about data analysis in one study, No concerns regarding coherence, No concerns regarding adequacy, and Very minor concerns regarding relevance: while the finding may not be directly related to healthcare services, it offers information about experiences during hospitalisation in general. | Clark et al. 2013; Haines 2005; Kupst et al. 1983; Neill et al. 2022; Scanferla et al. 2020; Scanferla et al. 2021; Sweeney et al. 2013; Wisemantel et al. 2018; |
| 3 | Theme: Parental emotional turmoil during hospitalisation \|\| The period of hospitalisation was an overwhelmingly difficult emotional experience for parents. Given the serious nature of the disease, parents' primary concern revolved around the survival of their child, with many expressing profound worry about this outcome. After receiving the diagnosis of meningitis, parents were shocked, confused, and daunted. The diagnosis was described as unexpected, as many parents had never considered it could happen to their child. Although the majority of parents described the experience of hospitalisation as traumatising, some parents found hospitalisation a relief, once their child was in a controlled hospital environment and receiving medical attention and care. The experience of hospitalisation in an ICU added to the emotional burden of parents whose children had a particularly severe illness. Parents were distressed, anxious, and emotionally unprepared to see changes in their child's appearance and behaviour caused by support equipment and treatments. | Serious concerns  **Explanation:** Serious concerns regarding methodological limitations. In two of the five studies that contributed to the finding recall bias could be introduced due to the time between the acute episode of meningitis and the interview. In one study this period was not reported. Relationship between researcher and participants was not reported in the two studies, one of which also did not provide sufficient details on data analysis. Overall, these methodological limitations were judged to have a potential influence on the finding. As the finding reflects emotional experiences during hospitalisation, recall bias could be critical in this case. | Minor concerns  **Explanation:** Minor concerns regarding coherence. The finding generally supports the underlying data and reflects the terrifying experience of caring for a hospitalised child with meningitis. Some less negative emotions, such as feeling the necessity to be strong for others or feeling lucky after recovery, are not included in the finding. However, this data was very scarce. | No/Very minor concerns  **Explanation:** Very minor concerns regarding adequacy. Five studies contributed to the finding: three offered thin data and two offered rich data, with one providing more insights on hospitalisation in general and the other on hospitalisation to ICU. | Minor concerns  **Explanation:** Minor concerns regarding relevance. While not necessarily commenting on experiences with healthcare services, the finding reflects the relevant experiences of hospitalisation in general. | Low confidence  **Explanation:** Serious concerns regarding methodological limitations: potential recall bias in three studies, which were judged to influence the finding. Minor concerns regarding coherence: the finding is consistent with the supporting data, but leaves out some experience with a less negative sentiment. No/Very minor concerns regarding adequacy. Minor concerns regarding relevance: the finding does not reflect experiences with healthcare services but hospitalisation in general. | Haines 2005; Kupst et al. 1983; Scanferla et al. 2021; Sweeney et al. 2013; Wisemantel et al. 2018; |
| 4 | Theme: Coping strategies and emotional support during hospitalisation \|\| Caregivers reported diverse experiences regarding emotional support during hospitalisation. While some expressed a need for additional support, such as counselling services, and noted that the assistance from healthcare workers (HCWs) was inadequate, others were satisfied with the care provided in hospitals or felt that support from family and friends was sufficient, eliminating the need for further psychological assistance during their admission. Additionally, caregivers identified several factors that helped them manage stress, including support from family members, shared responsibilities with relatives and friends, interactions with other parents in the hospital, intervenors, religious beliefs, the attentiveness and hospitality of HCWs, the quality of medical care, prior experiences, and a positive attitude. | Serious concerns  **Explanation:** Serious concerns regarding methodological limitations. In three of the five studies that contributed to the finding, recall bias could be introduced due to the time between the acute episode of meningitis and the interview. In one study, this period was not reported. This limitation was judged to have a potentially significant influence on participants' responses. Additionally, the two studies did not report the relationship between researchers and participants, and one did not provide sufficient details on data analysis. As the finding reflects experiences during hospitalisation and attitudes towards healthcare workers' support, recall bias and lack of reflexivity could be critical in this case. | No/Very minor concerns  **Explanation:** No concerns regarding coherence. The finding completely reflects the range of the underlying data. | No/Very minor concerns  **Explanation:** No concerns regarding adequacy. Three out of the five studies contributing to the finding offered rich data. Overall, the richness and quantity of all data provided by the five studies were considered to be high, so we concluded that there are no concerns about adequacy. | Moderate concerns  **Explanation:** Indirect relevance. The finding provides information about the emotions and psychological coping strategies of caregivers during hospitalisation, which is not directly related to experiences with healthcare services. | Low confidence  **Explanation:** Serious concerns regarding methodological limitations: potential recall bias in three out of five studies, no reflexivity statement in two studies, insufficient information about data analysis in one study, No concerns regarding coherence, No concerns regarding adequacy, and Moderate concerns regarding relevance: the finding is not directly related to experience with the healthcare services. | Haines 2005; Kupst et al. 1983; Scanferla et al. 2021; Sweeney et al. 2013; Wisemantel et al. 2018; |
| HOSPITALISATION – LMICS | | | | | | | |
| 5 | Theme: Perceptions of lumbar puncture outcomes \| Patients and caregivers perceived LP as a potentially fatal procedure associated with adverse outcomes. Most notably, the fear of death and paralysis emerges as a dominant concern. Death was attributed to delayed procedure uptake or a patient’s poor overall condition, along with concerns that the patient's position during or after the procedure could lead to paralysis. These perceptions were further fueled by second-hand experiences with adverse outcomes. However, patients and caregivers have observed improvements in the outcomes of LP in recent years, which they attributed to advances in procedural techniques and, in some instances, to divine intervention. | No/Very minor concerns  **Explanation:** Very minor concerns regarding methodological limitations because one study supporting the finding lacks a reflexivity statement. However, this limitation was assessed as not having a significant influence on the finding. | No/Very minor concerns  **Explanation:** Since the finding is based on one study and reflects all the relevant data, we had no concerns regarding coherence. | Moderate concerns  **Explanation:** Moderate concerns regarding adequacy because only one study contributed to the finding, even though it provided moderately rich data and had a relatively large sample size. | Minor concerns  **Explanation:** Minor concerns regarding relevance because even though the finding provides relevant information about values and experiences with a specific healthcare service, only one study contributed to the finding. | Moderate confidence  **Explanation:** Very minor concerns regarding methodological limitations: no reflexivity in one study, No concerns regarding coherence, Moderate concerns regarding adequacy: one study with moderately rich data contributed to the finding, and Minor concerns regarding relevance: relevant data about the experience with LP, but only from one study. | Elafros et al. 2022; |
| 6 | Theme: Economic impact of medical treatment on families \| Orthodox treatment carried a great financial burden for families: medicaments and prescriptions were costly, requiring caregivers to sell their properties and incur substantial debts to afford treatment expenses. The lack of funds was the primary reason for seeking alternative types of treatment before going to the hospital. | Minor concerns  **Explanation:** Minor concerns regarding methodological limitations. The single contributing study did not report sufficient information on the relationship between researcher and participants. Considering the sensitive nature of the finding, this limitation could have influenced participants' responses. However, we concluded it would be unlikely. | No/Very minor concerns  **Explanation:** Since the finding is based on one study and reflects all the relevant data, we had no concerns regarding coherence. | Moderate concerns  **Explanation:** Moderate concerns regarding adequacy. One study contributed to the finding, offering relatively thin data. Considering the finding is rather descriptive, we concluded that we had moderate concerns about data adequacy. | No/Very minor concerns  **Explanation:** The finding reflects one of the major barriers to healthcare services, therefore, we had no concerns regarding relevance. | Moderate confidence  **Explanation:** Minor concerns regarding methodological limitations: there were concerns about reflexivity and potential recall bias in the single contributing study. Considering the sensitive nature of the finding, this limitation could have influenced participants' responses. No/Very minor concerns regarding coherence. Moderate concerns regarding adequacy: one study contributed to the finding, offering relatively thin data. No/Very minor concerns regarding relevance. | Griffiths et al. 2012; |
| 7 | Theme: Challenges in diagnosing meningitis \| Healthcare workers claimed knowledge of meningitis signs and symptoms but highlighted difficulties in diagnosing the disease. Meningitis was sometimes misdiagnosed as malaria, resulting in the prescription of anti-malarial medication. | Serious concerns  **Explanation:** Serious concerns regarding methodological limitations. In one study that contributed to the finding, the relationship between researchers and participants is not stated. This limitation was assessed to be critical since the finding describes the experiences of healthcare workers. Additionally, it is unclear whether the research design of the study was appropriate to address the aims of the research. | No/Very minor concerns  **Explanation:** No concerns regarding coherence. | Serious concerns  **Explanation:** Serious concerns regarding adequacy because only one study supports the finding, offering thin data. | No/Very minor concerns  **Explanation:** No concerns about relevance because the finding highlighted the experiences of healthcare workers in providing care for patients with meningitis, particularly in misdiagnosing meningitis with malaria. | Very low confidence  **Explanation:** Serious concerns regarding methodological limitations: no reflexivity statement in one study, concerns about research design, No concerns regarding coherence, Serious concerns regarding adequacy: only one study with thin data, and No concerns regarding relevance | Desmond et al. 2013; |
| 8 | Theme: Miscommunication between HCWs and end-users \| Narratives from healthcare workers and patients revealed that it is common for patients to be verbally mistreated, which affects their decision to seek help at conventional healthcare facilities. Doctors stated that patients can be shouted at, disregarded, and blamed, specifically for failing to control the symptoms of meningitis. | Moderate concerns  **Explanation:** Moderate concerns regarding methodological limitations. The one contributing study did not provide sufficient data on the relationship between the researcher and participants. Considering that the finding reflects participant experiences with and attitudes towards healthcare services, this limitation was judged to potentially influence the finding. Additionally, recall bias could have been introduced, which would not be expected to influence the finding. | No/Very minor concerns  **Explanation:** The finding fully reflects data from the single contributing study, hence, there were no concerns regarding coherence. | Serious concerns  **Explanation:** Serious concerns regarding adequacy because only one study contributed to the finding, offering relatively thin data. | No/Very minor concerns  **Explanation:** No concerns regarding relevance. The finding reflects HCWs' experiences with healthcare services provision. | Low confidence  **Explanation:** Moderate concerns regarding methodological limitations: no reflexivity and potential recall bias. No concerns regarding coherence. Serious concerns regarding adequacy: only one study with relatively thin data. No concerns regarding relevance | Desmond et al. 2013; |
| 9 | Theme: Fear of complications as a barrier to LP uptake \| Patients and caregivers expressed fear about potential complications related to lumbar punctures (LP), specifically death and paralysis. These concerns were shaped by previous negative experiences with the procedure and the historical association of LP with high mortality rates during the HIV epidemic. | Moderate concerns  **Explanation:** Moderate concerns regarding methodological limitations. The one contributing study did not provide sufficient data on the relationship between the researcher and participants. Considering that the finding reflects participants' attitudes towards healthcare services, this limitation was judged to potentially influence the finding. | No/Very minor concerns  **Explanation:** The finding fully reflects data from the single contributing study, hence, there were no concerns regarding coherence. | Moderate concerns  **Explanation:** Moderate concerns regarding adequacy. Only one study contributed to the finding, offering relatively thin data. Considering the finding is rather descriptive, we concluded that there are moderate concerns about data adequacy, even though only one study contributed to the finding. | No/Very minor concerns  **Explanation:** No concerns regarding relevance because the finding reflects factors influencing the uptake of healthcare services. | Low confidence  **Explanation:** Moderate concerns regarding methodological limitations: no reflexivity statement in one study, No concerns regarding coherence, Moderate concerns regarding adequacy: only one study with relatively thin but descriptive data, and No concerns regarding relevance | Elafros et al. 2022; |
| 10 | Theme: Reliance on shared decision-making \| The decision to consent to a medical procedure was influenced by the shared nature of decision-making. Caregivers and patients consulted older family members when deciding to consent to LP, in some cases, to share responsibility and avoid being blamed. Even when the patient was sufficiently well to make their own medical decisions, family consensus may have overruled patient wishes, especially if too much time passes between consent and procedure completion. | No/Very minor concerns  **Explanation:** Very minor concerns regarding methodological limitations because one study supporting the finding lacks a reflexivity statement. However, this limitation was assessed as not having a significant influence on the finding. | No/Very minor concerns  **Explanation:** The finding reflects data from the single contributing study, hence, there were no concerns regarding coherence. | Moderate concerns  **Explanation:** Moderate concerns regarding adequacy. Only one study contributed to the finding, offering moderately rich data that is rather large in quantity. Considering the finding is rather descriptive, we concluded that there are moderate concerns about data adequacy, even though only one study contributed to the finding. | No/Very minor concerns  **Explanation:** No concerns regarding relevance because the finding reflects factors influencing the uptake of healthcare services. | Moderate confidence  **Explanation:** Very minor concerns regarding methodological limitations: no reflexivity, No concerns regarding coherence, Moderate concerns regarding adequacy: one study with moderately rich data, and No concerns regarding relevance | Elafros et al. 2022; |
| 11 | Theme: Patients' values driving LP consent \| Patients identified several factors influencing their decision to consent to LP. Trust in physicians and confidence in their technical abilities facilitated the acceptance of the procedure. The desire for diagnostic clarity and appropriate treatment also served as a justification for LP, with some viewing the procedure as a way to potentially reduce the length of hospital stays. Concern over the patient’s health and disease progression further motivated families to agree, typically later in the illness course. | Moderate concerns  **Explanation:** Moderate concerns regarding methodological limitations. The one contributing study did not provide sufficient data on the relationship between the researcher and participants. Considering that the finding reflects participants' attitudes towards healthcare services, this limitation was judged to potentially influence the finding. | No/Very minor concerns  **Explanation:** The finding fully reflects data from the single contributing study, hence, there were no concerns regarding coherence. | Moderate concerns  **Explanation:** Moderate concerns regarding adequacy. Only one study contributed to the finding, offering moderately rich data. Considering the finding is rather descriptive, we concluded that there are moderate concerns about data adequacy, even though only one study contributed to the finding. | No/Very minor concerns  **Explanation:** No concerns regarding relevance because the finding reflects factors influencing the uptake of healthcare services. | Low confidence  **Explanation:** Moderate concerns regarding methodological limitations: no reflexivity statement. No concerns regarding coherence. Moderate concerns regarding adequacy: one study with moderately rich, but descriptive data. No concerns regarding relevance | Elafros et al. 2022; |
| 12 | Theme: Systemic and operational barriers in healthcare organisation. Reports amongst healthcare workers highlighted several issues related to the organisation of healthcare services. These include poor hospital logistics, lack of sterility, risk of iatrogenic infections, time constraints, and the requirement for a CT scan prior to performing a lumbar puncture. Additionally, some healthcare workers addressed the lack of expertise and knowledge about contraindications of lumbar puncture among doctors, with some citing it as a reason they would be hesitant to undergo or perform the procedure. Drug supply shortages were also noted in both epidemic and non-epidemic settings. In epidemic setting, it was reported that healthcare workers were often unaware of official guidelines regarding different payment schedules, which led to increased out-of-pocket expenses for medications, despite official government policy. | Moderate concerns  **Explanation:** Moderate concerns regarding methodological limitations because all three studies contributing to the finding lack reflexivity statements. Considering the finding describes views of healthcare workers on healthcare organisation, lack of reflexivity could potentially influence the finding. In one study, it is unclear whether recall bias could be introduced. Another study did not provide sufficient information about data analysis. However, these limitations were judged to have minimal effect on the finding. | No/Very minor concerns  **Explanation:** The finding fully reflects data from the single contributing study, hence, there were no concerns regarding coherence. | Minor concerns  **Explanation:** Minor concerns regarding adequacy because one of three contributing studies offered moderately rich data, while two others provided thin data. Additionally, two studies offered information only about drug availability. | No/Very minor concerns  **Explanation:** No concerns regarding relevance because the finding reflects factors influencing the provision of healthcare services. | Moderate confidence  **Explanation:** Moderate concerns regarding methodological limitations: no reflexivity, concerns about data analysis and potential recall bias, No concerns regarding coherence, Minor concerns regarding adequacy: one study with moderately rich data, two studies with thin data, and No concerns regarding relevance | Colombini et al. 2009; Desmond et al. 2013; Elafros et al. 2022; |
| 13 | Theme: Community apprehensions influencing HCW's decision making. Healthcare workers (HCWs) reported that community apprehensions influenced their decision to refer patients for LP. HCWs were reluctant to perform LPs on terminally ill patients due to concerns that if the patient died shortly after, others might perceive the procedure as the cause of death. While caregivers were often more willing to consent to LPs later in the admission, HCWs believed that at this stage, the procedure would no longer have a meaningful impact on patient outcomes. | Moderate concerns  **Explanation:** Moderate concerns regarding methodological limitations. The one contributing study did not provide sufficient data on the relationship between the researcher and HCWs. Considering that the finding reflects the factors influencing the provision of the healthcare service, this limitation was judged to potentially influence the finding. | No/Very minor concerns  **Explanation:** The finding fully reflects data from the single contributing study, hence, there were no concerns regarding coherence. | Moderate concerns  **Explanation:** Moderate concerns regarding adequacy. Only one study contributed to the finding, offering relatively thin data. Considering the finding is rather descriptive, we concluded that there are moderate concerns about data adequacy, even though only one study contributed to the finding. | No/Very minor concerns  **Explanation:** No concerns regarding relevance because the finding reflects factors influencing the provision of healthcare services by healthcare workers. | Low confidence  **Explanation:** Moderate concerns regarding methodological limitations: no reflexivity statement, which might have influenced HCWs' replies, No concerns regarding coherence, Moderate concerns regarding adequacy: only one study with thin data, and No concerns regarding relevance | Elafros et al. 2022; |
| 14 | Theme: Consent practices facilitating LP delivery \| Firstly, consent was provided only verbally, as it was the norm for LP in this area. By omitting written consent, healthcare workers believed to prevent patients from having misconceptions about the procedure. While consent was obtained only verbally, the LP refusal was formally documented in medical records. Secondly, healthcare workers prioritised patient care over the consent process to save time. While some entirely skipped the consent process, others modified it to obtain consent more rapidly: they recalled manipulating risks (minimising or omitting) of LP during the consent process to reduce the probability of LP refusal. Finally, healthcare workers believed that by clearly explaining the purpose of LP and effectively communicating with patients, they were more likely to obtain consent. | Moderate concerns  **Explanation:** Moderate concerns regarding methodological limitations. The study did not provide information about the relationship between researcher and participants. As the finding reflects a rather sensitive topic of consent practices reported by healthcare workers themselves, this limitation was judged to potentially influence the finding. | No/Very minor concerns  **Explanation:** No concerns regarding coherence. The finding fully reflects the range of consent practices reported in the study. | Minor concerns  **Explanation:** Minor concerns regarding adequacy. One study contributed to the finding, offering moderately rich and sufficiently large quantity data. | No/Very minor concerns  **Explanation:** No concerns regarding coherence because the finding encompasses experiences with and facilitators of provision of healthcare services for meningitis. | Moderate confidence  **Explanation:** Moderate concerns regarding methodological limitations: as the finding reflects consent practices reported by healthcare workers themselves, lack of reflexivity was judged to potentially influence the finding. No/Very minor concerns regarding coherence. Minor concerns regarding adequacy: one study contributed to the finding, offering moderately rich and sufficiently large in quantity data. No/Very minor concerns regarding relevance. | Elafros et al. 2022; |
| POST-HOSPITALISATION – HICS | | | | | | | |
| 15 | Theme: Multifaceted impact of meningitis on physical, mental, and social well-being \|\| Meningitis survivors and their carers reported disabling physical and mental health sequelae of meningitis, causing daily frustration and limitations in social activities. Among some of the cited sequelae were limb loss, hearing and vision impairment, paralysis, depression, memory impairment in adults, and developmental delay in children. Additionaly, meningitis survivors highlighted the impact of these sequelae on their social and personal activities and life perspectives. Some patients had to change career, education and family plans, while others reported social isolation and changes in personal motivation. Rehabilitation was an additional source of daily psychological distress, especially when patients were involved in long periods of rehabilitation. | Moderate concerns  **Explanation:** Moderate concerns regarding methodological limitations. In two of the three studies that contributed to the finding, recall bias could be introduced due to the time between the acute episode of meningitis and the interview. Recall bias was judged to have a minimal impact on the finding, as it reflects potentially more recent events of dealing with meningitis after-effects and not the acute episode. One study did not provide sufficient details on study design and reflexivity, raising concerns about the methodological quality of the provided qualitative data. | Minor concerns  **Explanation:** Minor concerns regarding coherence. While the finding is generally coherent with the underlying data, it reflects general patterns and not the full range of experiences with meningitis sequelae. Additionally, the finding mostly focuses on data from adult patients but includes little data on parental and childhood experiences. | No/Very minor concerns  **Explanation:** No concerns regarding adequacy. Three studies contributed to the finding: two offered rich data and one offered moderately rich data. | Serious concerns  **Explanation:** Indirect relevance. Serious concerns regarding relevance. The finding does not reflect experiences of end-users with rehabilitation services but with meningitis sequelae in general. | Low confidence  **Explanation:** Moderate concerns regarding methodological limitations: potential recall bias in two studies, which is judged to have minimal impact on the finding. Concerns about study design, reflexivity, and ethical approval in one study, which raised concerns about the quality of the provided qualitative data. Minor concerns regarding coherence: generally coherent, but mostly reflects major patterns in the underlying data. No/Very minor concerns regarding adequacy. Serious concerns regarding relevance: indirect relevance; does not reflect experience with healthcare services but meningitis sequelae in general. | Erickson et al. 2001; Scanferla et al. 2020; Scanferla et al. 2021; |
| 16 | Theme: Long-term psychological impact of hospitalisation \|\| Some patients had traumatic experiences and developed phobias related to medical procedures, personnel or hospital environment following their hospitalisation. In some cases, these fears persisted long after their recovery. The psychological impact of meningitis hospitalisation also manifested in distress, feelings of helplessness and depression among both survivors and their caregivers. | Moderate concerns  **Explanation:** Moderate concerns regarding methodological limitations. In four of the six studies that contributed to the finding, recall bias could be introduced due to the time between the acute episode of meningitis and the interview. In one study, this period was not reported. The two studies did not report the relationship between the researcher and participants, one of which also did not provide sufficient details on data analysis. Overall, these methodological limitations could potentially influence the finding, so we concluded that there are moderate concerns about methodology. | Minor concerns  **Explanation:** Minor concerns regarding coherence. While the finding is coherent with the underlying data, it reflects general patterns and not the full range of emotions, phobias, and a range of depressive symptoms experienced by patients and caregivers. | No/Very minor concerns  **Explanation:** Very minor concerns regarding adequacy. Together, six studies provided moderately rich and consistent data. | No/Very minor concerns  **Explanation:** Direct relevance. The finding describes experience with healthcare services and the impact of their provision. | Moderate confidence  **Explanation:** Moderate concerns regarding methodological limitations: potential recall bias in four studies, no reflexivity statement in two studies, insufficient information about data analysis in one study, Minor concerns regarding coherence: the finding lacks specific descriptions of psychological changes, Very minor concerns regarding adequacy: the overall richness of data was considered to be moderate, and No/Very minor concerns regarding relevance | Haines 2005; Kupst et al. 1983; Scanferla et al. 2020; Scanferla et al. 2021; Sweeney et al. 2013; Wisemantel et al. 2018; |
| 17 | Theme: Parental concerns about potential consequences of meningitis \|\| Caregivers of children who had survived meningitis were concerned about potential long-term consequences of the illness. Some parents questioned if their child's health, development, and behaviour were normal or if any abnormalities could be treated as after-effects of meningitis. Others were anxious about child's future and well-being in general. | Minor concerns  **Explanation:** Minor concerns regarding methodological limitations. In two of the four studies that contributed to the finding, recall bias could be introduced due to the time between the acute episode of meningitis and the interview. In one study this period was not reported. Recall bias was judged to have a minimal impact on the finding, as it reflects potentially more recent events of dealing with meningitis consequences and not the acute episode. Relationship between researcher and participants was not sufficiently reported in the two studies. Additionally, one study did not provide sufficient details about data analysis, raising concerns about this methodological aspect. We concluded, that these limitations were unlikely to influence the finding. | No/Very minor concerns  **Explanation:** No concerns regarding coherence. The finding fully reflects the underlying data. | Moderate concerns  **Explanation:** Moderate concerns regarding adequacy. Four studies contributed to the finding: all offered relatively superficial data, with one of them providing data that was large in quantity. | Moderate concerns  **Explanation:** Moderate concerns regarding relevance. While the finding hints towards a lack of support from aftercare services, it explicitly covers only the experience of caregiving for a child with meningitis sequelae. | Low confidence  **Explanation:** Minor concerns regarding methodological limitations: potential recall bias in three studies, which was judged to minimally influence the finding. Insufficient data on reflexivity in two studies and on data analysis in one. These limitations were judged to unlikely influence the finding. No/Very minor concerns regarding coherence, Moderate concerns regarding adequacy: all four studies offered thin data, but one of them provided data from a large sample (n=244). Moderate concerns regarding relevance: the finding mostly reflects general experience with caregiving and not healthcare services. | Kupst et al. 1983; Scanferla et al. 2021; Sweeney et al. 2013; Wisemantel et al. 2018; |
| 18 | Theme: Need for care continuity, education and support \|\| Caregivers emphasized the need for prolonged care, support and education regarding meningitis sequelae. Parents wanted reassurance from healthcare workers about their child's medical condition and highlighted the importance of follow-up appointments, additional medical tests, and specialist assessments to evaluate potential sequelae and identify any special needs. Furthermore, some caregivers reported a lack of knowledge about meningitis sequelae and expressed a desire for more information. Additionally, parents noted feeling overwhelmed and distressed following the diagnosis and indicated a need for psychological support after their child's discharge. | Moderate concerns  **Explanation:** Moderate concerns regarding methodological limitations. In three of the five studies that contributed to the finding, recall bias could be introduced due to the time between the acute episode of meningitis and the interview. In one study, this period was not reported. The two studies did not report the relationship between the researcher and participants, one of which also did not provide sufficient details on data analysis. Overall, these methodological limitations were judged to potentially influence the finding, so we concluded that there are moderate concerns regarding methodology. | No/Very minor concerns  **Explanation:** No concerns regarding coherence. The finding completely reflects the range of the underlying data. | No/Very minor concerns  **Explanation:** Very minor concerns regarding adequacy. Out of five studies, one provided very rich data, another one – moderately rich, and three other studies – only thin data. However, the overall richness of data was assessed as high. | Moderate concerns  **Explanation:** Indirect relevance. The finding is not directly related to the values and experiences with healthcare services. However, while the finding may not be directly related to healthcare services, it offers information about experiences with meningitis sequelae in general. | Low confidence  **Explanation:** Moderate concerns regarding methodological limitations: potential recall bias in three studies, no reflexivity statement in two studies, insufficient information about data analysis in one study, No concerns regarding coherence, Very minor concerns regarding adequacy, and Moderate concerns regarding relevance: the finding is not directly related to the values and experiences with healthcare services, but it offers information about experiences with meningitis sequelae in general. | Clark et al. 2013; Haines 2005; Scanferla et al. 2021; Sweeney et al. 2013; Wisemantel et al. 2018; |
| 19 | Theme: Perceptions of quality of aftercare for meningitis sequelae \|\| Parents of children with meningitis sequelae had contrasting experiences with aftercare services. Some parents were unhappy with the provided care and reported inadequate customisation of prosthetic limbs and orthopaedic devices. Carers also noted poor communication between different members of the aftercare process and different anticipated goals of rehabilitation, which delayed timely and sufficient care. On the other hand, when parents were satisfied with the provided rehabilitation services, aftercare was tailored and suitable for their child’s needs. Effective communication and listening to parents' expectations of the process were seen to play a crucial role in good care. | Moderate concerns  **Explanation:** Moderate concerns regarding methodological limitations. One study that contributed to the finding did not provide any information on reflexivity. As the finding reflects experiences with and attitudes towards healthcare services, this limitation was judged to potentially influence the finding. | No/Very minor concerns  **Explanation:** No concerns regarding coherence. The finding fully reflect the underlying data. | Minor concerns  **Explanation:** Minor concerns regarding adequacy. Only one study contributed to the finding, offering moderately rich data. Given the finding is exploratory and rather superficial, we concluded we have minor concerns regarding adequacy. | No/Very minor concerns  **Explanation:** Direct relevance. No concerns regarding relevance. The finding answers the review question by providing data on caregivers' experience with aftercare services. | Moderate confidence  **Explanation:** Moderate concerns regarding methodological limitations: no data to judge if reflexivity was adequate, which could have influenced the finding. No/Very minor concerns regarding coherence, Minor concerns regarding adequacy: one study offered rich data, which was considered adequate for a descriptive finding. No/Very minor concerns regarding relevance | Clark et al. 2013; |
| 20 | Theme: Lack of appreciation for less apparent sequelae of meningitis \|\| A lack of recognition and understanding of the less visible psychosocial and cognitive after-effects of meningitis hindered parental ability to access support services, particularly in educational settings. Young age acted as an additional barrier to gaining access to aftercare because of difficulty testing young children, misconceptions about the needs of disabled children and challenges in predicting cognitive after-effects at the time of discharge. | No/Very minor concerns  **Explanation:** Very minor concerns regarding methodological limitations. One study contributing to the finding did not report the relationship between the researcher and participants. However, this limitation was judged as unlikely to influence the finding. | No/Very minor concerns  **Explanation:** No concerns regarding coherence. The finding completely reflects the range of the underlying data. | Moderate concerns  **Explanation:** Moderate concerns regarding adequacy because only one study provides relatively thin data. Given the descriptive nature of the finding, we concluded that there are moderate concerns regarding adequacy. | Minor concerns  **Explanation:** Minor concerns regarding relevance. While the finding does not directly describe the experiences of caregivers with healthcare services, it provides information about potential barriers that make it difficult to gain access to healthcare services for patients with meningitis sequelae. | Moderate confidence  **Explanation:** Very minor concerns regarding methodological limitations: no reflexivity statement in one study, No concerns regarding coherence, Moderate concerns regarding adequacy: only one study with relatively thin data, and Minor concerns regarding relevance: while the finding does not directly describe the experiences with healthcare services, it provides information about potential barriers to gaining access for patients with meningitis sequelae. | Clark et al. 2013; |
| 21 | Theme: Systemic and organisational barriers to aftercare \|\| Parents of childhood meningitis survivors reported difficiulties with accessing and navigating aftercare services, including disability living allowance and social care, and expressed a need for support. Carers cited factors such as lack of staff, lack of communication between different members of the process, restricted budget, and complex bureaucratic procedures as barriers to timely, sufficient, and tailored rehabilitation. Additionaly, they emphasised on the limited inclusion criteria, which posed significant barriers for young children and those with cognitive or psychological sequelae. Impairments in these children was sometimes borderline and less apparent, and aftercare services failed to recognise the link between meningitis and such non-physical sequelae, further complicating access to rehabilitation. | Minor concerns  **Explanation:** Minor concerns regarding methodological limitations. In one of the three studies that contributed to the finding, recall bias could be introduced due to the time between the acute episode of meningitis and the interview. In one study this period was not reported. Relationship between researcher and participants was not sufficiently reported in one study. These limitations were judged to have a minimal impact on the finding, as it reflects potentially more recent events during rehabilitation and not the acute episode. Additionally, the study where the recall bias is more likely contributed only thin data. | No/Very minor concerns  **Explanation:** No concerns regarding coherence. The finding fully reflects the underlying data. | Minor concerns  **Explanation:** Minor concerns regarding adequacy. Three studies contributed to the finding: two offered thin data, and one offered rich data. Due to the descriptive and superficial nature of the finding, we concluded the data is sufficient and we have minor concerns regarding limitations. | No/Very minor concerns  **Explanation:** Direct relevance. No concerns regarding relevance. The finding answers the review question by providing insights into barriers to meningitis rehabilitation services. | High confidence  **Explanation:** Minor concerns regarding methodological limitations: potential recall bias in two of the three studies, no reflexivity in one. These limitations would unlikely influence the finding, given its' more generic scope. No/Very minor concerns regarding coherence. Minor concerns regarding adequacy: one study offered rich data, and two offered thin data. The data were judged as adequate, as the finding is descriptive. No/Very minor concerns regarding relevance | Clark et al. 2013; Scanferla et al. 2021; Sweeney et al. 2013; |
| 22 | Theme: Third parties as facilitators of meningitis sequelae aftercare \|\| Parents highly valued tailored care suitable for their child’s needs. The school was seen as having a special role to play in providing accessible, long-term and timely follow-up care. Additionally, parents reported the active involvement of a consultant and multidisciplinary team meetings including parents, school staff, and health visitors as factors helping overcome difficulties in accessing aftercare. | No/Very minor concerns  **Explanation:** Very minor concerns regarding methodological limitations. One study contributing to the finding did not report the relationship between the researcher and participants. However, this limitation was judged as unlikely to influence the finding. | No/Very minor concerns  **Explanation:** No concerns regarding coherence. The finding completely reflects the range of the underlying data. | Moderate concerns  **Explanation:** Moderate concerns regarding adequacy because only one study provides relatively thin data. Given the descriptive nature of the finding, we concluded that there are moderate concerns regarding adequacy. | Minor concerns  **Explanation:** Minor concerns regarding relevance. While the finding does not directly describe the experiences of caregivers with healthcare services, it provides information about potential factors that improve access to healthcare services for patients with meningitis sequelae. | Moderate confidence  **Explanation:** Very minor concerns regarding methodological limitations: no reflexivity statement in one study, No concerns regarding coherence, Moderate concerns regarding adequacy: only one study with relatively thin data, and Minor concerns regarding relevance: while the finding does not directly describe the experiences with healthcare services, it provides information about potential facilitators to gaining access for patients with meningitis sequelae. | Clark et al. 2013; |
| PRE-HOSPITALISATION – HICS | | | | | | | |
| 23 | Theme: Need for awareness and comprehensive knowledge about meningitis \|\| Patients and caregivers had little or no prior knowledge about meningitis etiology and symptoms before the diagnosis. Once meningitis was diagnosed, people expressed a strong desire for more information about the disease, often approaching the internet or contacting associations to fill in the gaps. Parents and carers were highlighting the need for better public education and awareness campains to recognise the signs of meningitis. | No/Very minor concerns  **Explanation:** Very minor concerns regarding methodological limitations. In three of the four studies that contributed to the finding, recall bias could be introduced due to the time period between the meningitis case and participation in the study. In one study this period could not be identified. Additionaly, there were concerns regarding reflexivity and data analysis in one study, as these aspects were not sufficiently reported. However, these limitations were judged to be unlikely to influence the finding, as it encompasses more general concepts around meningitis rather than individual. | No/Very minor concerns  **Explanation:** Very minor concerns regarding coherence. While some parents were aware of several symptoms of meningitis, their overall knowledge was still considered insufficient. | No/Very minor concerns  **Explanation:** No concerns about adequacy. Four studies contributed to the finding: one offered rich data, and the others together provided rich data as well. Given that the finding is descriptive and that one study offered comprehensive data, we concluded that we have no concerns regarding adequacy. | Serious concerns  **Explanation:** Indirect relevance. Serious concerns regarding relevance. The finding does not reflect the experiences of end-users with healthcare services but with meningitis in general. | Low confidence  **Explanation:** Very minor concerns regarding methodological limitations: concerns about reflexivity, data analysis and recall bias, Very minor concerns regarding coherence, No concerns regarding adequacy, and Serious concerns regarding relevance: indirect relevance; does not reflect experience with healthcare services but meningitis in general. | Scanferla et al. 2020; Scanferla et al. 2021; Sweeney et al. 2013; Wisemantel et al. 2018; |
| 24 | Parental emotional reactions during initial stages of meningitis \|\| Prior to hospitalisation, parents experienced complex emotions ranging from anger and disbelief to fear and a sense of loss of control. Parents revealed they experienced immense fear in front if a serious disease, which sometimes lead to denial of the illness at all. The fear was exacerbated by the sense of helplessness and loss of control over child's condition. Feelings of anger and disbelief came over when parents failed to find the expected confirmation of their concerns and appropriate support during first contact with healthcare services. Transportation to the healthcare facility was another source of emotional burden during the initial staged of the illness. Parents were stressed when they were excluded from accompanying their child, but understanding the rationale behind it and involvement of a team of health specialists helped to ease the stress. | Moderate concerns  **Explanation:** Moderate concerns regarding methodological limitations. In one of the three contributing studies, recall bias could be introduced due to the time passed between the acute episode of meningitis and the interview. Considering the finding reflects caregivers' emotional experiences prior to hospitalisation, the potential recall bias could affect the finding. Additionally, two of the three studies did not provide sufficient information on the relationship between researcher and participants. Lack of reflexivity was judged as unlikely to influence the finding, as is does not cover the direct experiences with healthcare services. | No/Very minor concerns  **Explanation:** No concerns regarding coherence. The finding fully reflect the supporting data. | Minor concerns  **Explanation:** Three studies contributed to the finding, together offering moderately rich data. Given the descriptive nature of the finding, we concluded we have minor concerns regarding adequacy. | Moderate concerns  **Explanation:** Indirect relevance. Moderate concerns regarding relevance. Overall, the finding focuses on emotional experiences of caregivers before hospitalisation. However, it provides some data on experiences with transportation to healthcare facilities and contact with healthcare services, hence, partially answering the review question. | Low confidence  **Explanation:** Moderate concerns regarding methodological limitations: potential recall bias in one study could affect the finding. No/Very minor concerns regarding coherence. Minor concerns regarding adequacy: three studies together offered moderately rich data, which was considered sufficient, given the finding is descriptive. Moderate concerns regarding relevance: the finding provides little data on experiences of caregivers with healthcare services before hospitalisation. | Haines 2005; Kupst et al. 1983; Neill et al. 2022; |
| 25 | Theme: Parental intuition and recognition of illness \|\| Intuition stimulated parents to seek medical attention, even when the symptoms did not immediately suggest meningitis. They recognised the overall deterioration of children's health based on subtle changes in their behaviour or physical condition. | Moderate concerns  **Explanation:** Moderate concerns regarding methodological limitations. In two of the four studies contributing to the finding, recall bias could be introduced due to the time period between the meningitis case and participation in the study. Considering the finding reflects caregivers' memories before hospitalisation, the potential recall bias could affect the finding. Additionally, there were concerns regarding reflexivity in three out of four studies, as well as about data analysis in one study, as these aspects were not sufficiently reported. However, these limitations were judged to be unlikely to influence the finding. | No/Very minor concerns  **Explanation:** Very minor concerns about coherence because, in one study, it is not directly stated that parental intuition stimulated help-seeking behaviour. However, this limitation was judged as unlikely to decrease the confidence in the finding. | Minor concerns  **Explanation:** Minor concerns regarding adequacy. Four studies contributed to the finding together offering moderately rich data. Due to the descriptive nature of the finding, we concluded that there are minor concerns about adequacy. | Minor concerns  **Explanation:** Indirect relevance. Even though the data does not reflect experiences with healthcare services in general, the finding describes the patterns of help-seeking behaviour that potentially influenced the uptake of these services, so we concluded that there are minor concerns about relevance. | Moderate confidence  **Explanation:** Moderate concerns regarding methodological limitations: concerns about recall bias, reflexivity, and data analysis, Very minor concerns regarding coherence: in one study, it is not explicitly stated that intuition stimulated help-seeking, Minor concerns regarding adequacy: moderately rich data, however, the finding is rather descriptive, and Minor concerns regarding relevance: the finding is indirectly related to the uptake of healthcare services. | Brennan et al. 2003; Haines 2005; Neill et al. 2022; Wisemantel et al. 2018; |
| 26 | Theme: Many masks of meningitis clinical presentation \|\| General practitioners reported having limited experience in diagnosing meningitis and meningococcal disease, which can present with diverse clinical manifestations and pose diagnostic challenges. Symptoms such as a non-blanching purpuric rash, neck pain, and rapid disease progression increased the certainty of a meningitis diagnosis. Conversely, non-specific symptoms were often less apparent to healthcare workers and could mislead the diagnostic process. Despite the uncertainty in the diagnosis of meningitis, doctors acknowledged that atypical clinical presentations might indicate a more serious underlying illness, prompting them to hospitalise such patients. Additional symptoms that led to the admission of these complex cases included lethargy, decreased mobility, altered consciousness and mental state, pallor, cyanosis, and abnormal crying. | Serious concerns  **Explanation:** Serious concerns regarding methodological limitations. In one of the two contributing studies recall bias could be introduced due to the time passed between treating a meningitis case and the interview. Additionally, one study did not provide sufficient information on the relationship between researcher and participants. Given the finding reflects healthcare workers' experiences with provision of medical care and provides rather detailed information, these limitations were judged to potentially influence the finding. | Minor concerns  **Explanation:** Minor concerns regarding coherence. While the finding is generally consistent with the underlying data, it reflects the major trends in doctors' experiences with different symptoms of meningitis. However, sometimes doctors had opposing experiences with some symptoms (purpuric rash, for example). | No/Very minor concerns  **Explanation:** No concerns regarding adequacy. Two studies contributed to the finding: one offered rich data and the other offered thin data. Given the finding is descriptive and one study offered comprehensive data, we concluded we have no concerns regarding adequacy. | No/Very minor concerns  **Explanation:** Direct relevance. No concerns regarding relevance. The finding directly answers the review question by providing insights into healthcare workers' experiences with diagnosing meningitis. | Low confidence  **Explanation:** Serious concerns regarding methodological limitations: potential recall bias in one study and lack of reflexivity in the other study, which could potentially influence the finding. Minor concerns regarding coherence: the finding does not reflect some minor opposing data but provides data on major trends in medical practice. No/Very minor concerns regarding adequacy. No/Very minor concerns regarding relevance | Brennan et al. 2003; Granier et al. ; |
| 27 | Theme: Role of context and parental input in clinical decision-making \|\| General practitioners (GPs) noted that parental anxiety significantly influenced their clinical decision-making, particularly when they had an established relationship with the family. Some parents expressed fear of meningitis that, according to healthcare workers, emerged as a result of the prevalence of awareness campaigns. GPs recognised that while these fears were sometimes disproportionate, awareness campaigns are still needed due to the severity of meningitis. Parental concerns sometimes served as facilitators for further clinical evaluation, with some parents directly prompting GPs to reconsider their initial assessments. | Moderate concerns  **Explanation:** Moderate concerns regarding methodological limitations. One of the two contributing studies did not provide enough information on the relationship between the researcher and participants. Given the finding reflects healthcare workers' experiences with the provision of medical care, lack of reflexivity could significantly influence the finding. Additionally, in one of the two contributing studies, recall bias could be introduced due to the time passed between the acute episode of meningitis and the interview, which limits our confidence in the provided data. Furthermore, one study did not explicitly describe ethical issues. | Minor concerns  **Explanation:** Minor concerns regarding coherence. While several GPs noted that parental fear of meningitis is disproportionate, only one stated that awareness campaigns are reasonable. | No/Very minor concerns  **Explanation:** No concerns about adequacy. Both studies contributing to the finding provide very rich data. | No/Very minor concerns  **Explanation:** Direct relevance. The finding describes the views and experiences of healthcare workers with the provision of healthcare services. | Moderate confidence  **Explanation:** Moderate concerns regarding methodological limitations: concerns about reflexivity, recall bias, and ethics, Minor concerns regarding coherence: several parts of the finding are supported only by one citation, No concerns regarding adequacy, and No concerns regarding relevance | Brennan et al. 2003; Granier et al. ; |
| 28 | Theme: Intuitive and evidence-based practice \|\| General practitioners (GPs) revealed that in general practice they rely much more on experience and intuition rather than evidence and logic. GPs acknowledged the utility of guidelines, but expressed skepticism about their application and noted challenges in keeping up with updates. They stressed that guidelines can undermine individualised and personal patient care and interfere with the more intuitive approach to diagnosis, which was deemed more helpful in case of an unusual clinical presentation. Moreover, GPs stated their priority was to identify a serious illness - where intuition was a key factor - rather than to make a definitive diagnosis . When identifying  a serious illness, general practitioners often recognised the overall poor condition, changes in usual behaviour of patients and 'puzzling' findings, rather than specific signs and symptoms. | Serious concerns  **Explanation:** Serious concerns regarding methodological limitations. Two of the three of contributing studies did not provide enough information on the relationship between researcher and participants. Given the finding reflects healthcare workers' experiences with provision of medical care, lack of reflexivity could significantly influence the finding. In one of the studies recall bias could be introduced due to the time passed since treating meningitis cases and the interview, which limits our confidence in the provided data. Additionally, one of the studies was a mixed-method study and did not provide enough information on qualitative data collection and analysis. However, this study contributes minimal data to the finding. | No/Very minor concerns  **Explanation:** No concerns regarding coherence. The finding fully supports the underlying data. | Minor concerns  **Explanation:** Minor concerns regarding adequacy. Three studies contributed to the finding: one offered rich data and two offered thin data. Due to the descriptive nature of the finding and comprehensive information from one study, we concluded we have minor concerns regarding adequacy. | No/Very minor concerns  **Explanation:** No concerns regarding relevance. The finding directly answers the review question by providing insights into healthcare workers' experiences with diagnosing meningitis. | Low confidence  **Explanation:** Serious concerns regarding methodological limitations: no reflexivity in two studies and potential recall bias in one, which could significantly influence the finding. Concerns about qualitative data collection and analysis in one study. No/Very minor concerns regarding coherence. Minor concerns regarding adequacy: one study offered rich and comprehensive data, while the other two offered thin data. No/Very minor concerns regarding relevance | Brennan et al. 2003; Granier et al. ; Jarvinen et al. 2005; |
| 29 | Theme: Sociocultural factors influencing help-seeking behaviour \|\| Caregivers expressed hesitance in seeking assistance due to concerns about the potential misuse or overuse of healthcare resources, particularly when uncertain about the severity of the illness. This reluctance to overutilise the healthcare system, along with other parental responsibilities, ultimately delayed their decision to seek medical attention. | Moderate concerns  **Explanation:** Moderate concerns regarding methodological limitations. In one study contributing to the finding, recall bias could be introduced due to the time passed since treating meningitis cases and the interview, which limits our confidence in the provided data. Additionally, the study did not provide enough information on the relationship between the researcher and participants. | No/Very minor concerns  **Explanation:** No concerns about coherence. The finding completely reflects the range of the underlying data. | Serious concerns  **Explanation:** Serious concerns regarding adequacy because only one study contributed to the finding offering thin data. | Minor concerns  **Explanation:** Indirect relevance. Even though the data is not explicitly related to the utilisation of healthcare services, the finding describes the patterns of help-seeking behaviour that potentially influenced the uptake of these services, so we concluded that there are minor concerns about relevance. | Low confidence  **Explanation:** Moderate concerns regarding methodological limitations: potential recall bias, no reflexivity, No concerns regarding coherence, Serious concerns regarding adequacy: one study offering thin data, and Minor concerns regarding relevance: the finding is indirectly related to the uptake of healthcare services. | Neill et al. 2022; |
| 30 | Theme: Systemic and operational barriers in healthcare organisation \|\| Healthcare workers in primary care settings reported not being confident with lack experience in treating meningitis, thus, they were more focused on getting the child hospitalized as early as possible rather than starting treatment on their own. The primary source of concern was lack of experience with administering parenteral antibiotics and potential difficulties with intravenous access. Additionally, GPs revealed that treatment could have been delayed due to advice or disapproval from clinical or perscription consultants and lack of immediate access to antibiotics. | Serious concerns  **Explanation:** Serious concerns regarding methodological limitations. All two of the contributing studies did not provide enough information on the relationship between researcher and participants. Given the finding reflects healthcare workers' experiences with provision of medical care, lack of reflexivity could significantly influence the finding. Additionally, one of the studies was a mixed-method study and did not provide enough information on qualitative data collection and analysis. However, this study contributes minimal data to the finding. | No/Very minor concerns  **Explanation:** No concerns regarding coherence. The finding fully reflect the underlying data. | Serious concerns  **Explanation:** Serious concerns regarding adequacy. Two studies contributed to the finding, offering thin data. While one of the studies provided some details and primary data to support the statements, the other lacked explanation. | No/Very minor concerns  **Explanation:** Direct relevance. No concerns regarding relevancy. The finding reflects general practitioners' experiences with providing care for patients with meningitis and barriers to provision, which answers the review question. | Very low confidence  **Explanation:** Serious concerns regarding methodological limitations: no reflexivity in two studies, which could significantly influence the finding. Concerns about qualitative data collection and analysis in one study. No/Very minor concerns regarding coherence. Serious concerns regarding adequacy: two studies offered thin data, one of which did not provide any details behind the data. No/Very minor concerns regarding relevance | Brennan et al. 2003; Jarvinen et al. 2005; |
| 31 | Theme: Factors influencing pre-hospital antibiotic treatment initiation \|\| General practitioners (GPs) were more likely to administer antibiotics pre-hospital when they were confident in their diagnosis. In cases with less certainty, the presence of severe symptoms sometimes stimulated GPs to take action. However, some GPs were hesitant to initiate treatment without definitive signs, preferring to wait until the diagnosis was clear. The presence of a non-blanching rash was identified as one of the most reliable indicators that led to the initiation of antibiotic treatment. | Moderate concerns  **Explanation:** Moderate concerns regarding methodological limitations. In one of the two contributing studies, recall bias could be introduced due to the time passed between treating a meningitis case and the interview, which could impact the responses of healthcare workers. Additionally, one study did not provide sufficient information on the relationship between the researcher and participants, but this limitation was judged as unlikely to influence the finding. | No/Very minor concerns  **Explanation:** No concerns about coherence. The finding completely reflects the range of the underlying data. | Moderate concerns  **Explanation:** Moderate concerns regarding adequacy because only two studies contributed to the finding together, offering relatively thin data. Given the finding is rather descriptive, we concluded we have no concerns regarding adequacy. | No/Very minor concerns  **Explanation:** Direct relevance. The finding provide information directly related to the provision of healthcare services. | Low confidence  **Explanation:** Moderate concerns regarding methodological limitations: in one study, a potential recall bias could be introduced, No concerns regarding coherence, Moderate concerns regarding adequacy: only two studies contributed to the finding offering relatively thin data, and No concerns regarding relevance | Brennan et al. 2003; Granier et al. ; |
| PRE-HOSPITALISATION – LMICS | | | | | | | |
| 32 | Theme: Knowledge and perceptions of meningitis \| Community members perceived meningitis as a dangerous disease typically presenting with stiff neck and seizures. Participants acknowledged that meningitis can result in death and disability, which was particularly scared of in children due to the potential loss of productivity and income in the future. Despite previous educational efforts, community's modern knowledge about the causes of meningitis was limited and centered around spiritual or supernatural influence. Some participants additionally referred to direct contact with an ill person, specific weather conditions, and foods that were associated with meningitis. | No/Very minor concerns  **Explanation:**  Very minor concerns regarding methodological limitations. Reflexivity statement was not provided in all four of the studies that contributed to the finding. Additionally, one study did not provide sufficient details on data analysis and one study did not report the time passed between the acute episode and interview. However, these limitations were judged to have minimal effect on the finding. | Moderate concerns  **Explanation:** Moderate concerns regarding coherence. Overall, the included studies were consistent with the finding. However, the finding captures only the most dominant patterns, while the data, especially on the known symptoms and sequelae of meningitis, were more varied. | No/Very minor concerns  **Explanation:** Very minor concerns regarding adequacy. Four studies contributed to the finding: two offered rich, and two offered moderately rich data. Additionally, data in all studies were large enough in quantity. | Serious concerns  **Explanation:** Serious concerns regarding relevance. Despite providing data on meningitis in general, the finding does not address experiences with healthcare services. | Low confidence  **Explanation:** Very minor concerns regarding methodological limitations. Moderate concerns regarding coherence: the finding captures only the most dominant patterns, while the data were more varied. Very minor concerns regarding adequacy. Serious concerns regarding relevance: the finding reflects experiences with meningitis in general, not with healthcare services. | Adedini et al. 2021; Colombini et al. 2009; Desmond et al. 2013; Mahmoud et al. 2022; |
| 33 | Theme: Conflict and convergence between biomedical and traditional treatment \| Many caregivers and patients showed a preference for biomedical treatment in managing meningitis, particularly after realizing the limitations of traditional healing methods. It was emphasised that the hospital was the preferred option for treatment due to doctors' expertise despite maintained spiritual beliefs about the disease's origins. However, other end-users still favoured traditional medicine. Some of them expressed doubt about the medical diagnosis of meningitis, attributing their illness to curses, dreams, or old age. | Moderate concerns  **Explanation:** Moderate concerns regarding methodological limitations. Two of the supporting studies did not clearly state the relationship between researchers and participants. This limitation was judged to potentially influence the finding since it is related to preferences for treatment type. | Minor concerns  **Explanation:** The finding reflects the complexity and variation of the data regarding the preferred treatment of meningitis. However, there are minor concerns regarding coherence because one of the assessed studies demonstrated the experience of caregivers and the community only, not the patients. | Moderate concerns  **Explanation:** Moderate concerns regarding adequacy because one of the two studies offers rich data, while the other provides only thin data. | No/Very minor concerns  **Explanation:** Minor concerns about relevance because although both studies provide information about end-users' values regarding meningitis treatment, one of the two studies provides thin data. | Low confidence  **Explanation:** Moderate concerns regarding methodological limitations: no reflexivity statement in two studies; Minor concerns regarding coherence: one of two studies does not provide information about the values of patients, while the other one offers data from both patients and caregivers; Moderate concerns regarding adequacy: one study offers rich data, while the other provides only thin data, and No/Very minor concerns regarding relevance: one study provides only thin data about end users' values on meningitis treatment. | Adedini et al. 2021; Mahmoud et al. 2022; |
| 34 | Theme: Sociocultural factors influencing health-seeking behaviour \| Health-seeking behaviour was largely influenced by the sociocultural norms established in the community. In a hierarchical society, patients and caregivers, especially women, usually sought validation of disease severity from senior and often male family or community members. In contrast to men, many women had limited or no formal education and were unemployed, which constrained their capacity to make independent decisions. Confirmation of disease severity was essential to warrant funding. However, it was commonly recognised only when the disease interfered with a patient's social activity, delaying timely care. Treatment preferences were also driven by the widespread perception among patients, carers of adult and paediatric patients, and community members that meningitis, in case of supernatural causes, should be treated with Islamic or traditional methods. Families consulted traditional healers despite acknowledging the effectiveness of conventional medicine and its availability, particularly to discern if the disease has supernatural origins. | Minor concerns  **Explanation:** Minor concerns regarding methodological limitations. Reflexivity statement was not provided in four out of five of the studies that contributed to the finding. Lack of reflexivity was judged to have a potential influence on the finding, as it encompasses views on preferred treatment. However, the main focus of the finding is the underlying reasons for the preferred treatment, hence, we concluded the influence would be minimal. Additionally, one study did not provide sufficient details on data analysis and one study did not report the time passed between the acute episode and interview. However, this limitation was judged to have minimal effect on the finding. | Moderate concerns  **Explanation:** Moderate concerns regarding coherence. All studies generally support at least one of the aspects of the finding. However, the finding captures only the most dominant patterns, leaving out contrasting patterns present in the 'gender inequalities' aspect of the finding. | No/Very minor concerns  **Explanation:** Very minor concerns regarding adequacy. Five studies contributed to the finding: two studies provided rich data, two - moderately rich, and one - thin data. The quantity of data was large enough. | No/Very minor concerns  **Explanation:** No concerns regarding relevance. The finding does not specifically address experiences with healthcare services, however, describes values and underlying causes of behaviour that may be considered a barrier to the uptake of these services. | Moderate confidence  **Explanation:** Minor concerns regarding methodological limitations: lack of reflexivity in four studies, which could influence the finding. Moderate concerns regarding coherence: the finding captures only the most dominant patterns, leaving out contrasting patterns present in the 'gender inequalities' aspect of the finding. No/Very minor concerns regarding adequacy. No concerns regarding relevance. | Adedini et al. 2021; Colombini et al. 2009; Desmond et al. 2013; Mahmoud et al. 2022; Omoleke et al. 2018; |
| 35 | Theme: Initial response regarding preferred treatment \| The initial response to disease signs in caregivers and patients involved self-medication and alternative treatment. Several families reported administering medications such as paracetamol to alleviate fever or headaches during the early stages of the illness. Caregivers also frequently mentioned favouring alternative medicine, including the help of prayers, traditional healers and soothsayers, to orthodox care. This preference was associated with the prevailing reliance on supernatural explanations for the illness and was particularly evident among older patients and caregiver groups who belonged to rural communities. Some caregivers also clarified that the reasons for favouring alternative medicine were the shorter waiting period, lower cost and less severe illness. Patronage of government hospitals was considered the last resort when the illness became severe and not amenable to alternative care. | Moderate concerns  **Explanation:** Moderate concerns regarding methodological limitations. In three out of four studies, the relationship between researchers and participants is not specified. Since the data describes attitudes towards preferred treatment, this limitation could potentially influence the finding. Additionally, in one study, it is unclear whether the research design was appropriate to address aims. Another study does not contain rich information about data analysis. | Minor concerns  **Explanation:** Minor concerns regarding coherence because the finding is more focused on alternative medicine than on self-medication. However, even though the finding miss some details from studies, overall, data from studies about self-medication is relatively superficial, so we concluded that there are only minor concerns about coherence. | No/Very minor concerns  **Explanation:** Very minor concerns regarding adequacy because three out of four studies offered rich data, while one study provided information only about self-medication as an initial response (thin data). | No/Very minor concerns  **Explanation:** No concerns about relevance all studies provide information about the end-users' values and experiences with meningitis treatment. | Moderate confidence  **Explanation:** Moderate concerns regarding methodological limitations: no reflexivity in three out of four studies, one study lacks information about data analysis, and in one study, it is unclear whether the research design was appropriate to address aims; Minor concerns regarding coherence: three studies reported both alternative medicine and self-medication as an initial response, while the other one – only self-medication, Very minor concerns regarding adequacy. No concerns regarding relevance. | Adedini et al. 2021; Colombini et al. 2009; Desmond et al. 2013; Omoleke et al. 2018; |
| 36 | Theme: Lack of awareness and alertness to meningitis symptoms delays timely care \| Community members, patients and caregivers often underestimated meningitis symptoms, attributing them to more familiar causes, such as malaria or traditional illnesses, that were usually treated at home. Some non-specific symptoms, including severe headache, body weakness and loss of appetite, were not considered a real illness, which was associated with the delay in timely help-seeking. | No/Very minor concerns  **Explanation:** Very minor concerns regarding methodological limitations. All three of the contributing studies did not provide any data on the relationship between researcher and participants. Additionally, one study did not report the time passed between the interview and the acute episode of meningitis. However, these limitations were judged to have a minimal influence on the finding. | No/Very minor concerns  **Explanation:** No concerns regarding coherence. The finding completely reflects the range of the underlying data. | Minor concerns  **Explanation:** Minor concerns regarding adequacy. Three studies contributed to the finding: one offered moderately rich data, and two offered thin data. However, due to the descriptive nature of the finding, the underlying data were considered adequate and raised minor concerns. | No/Very minor concerns  **Explanation:** No concerns regarding relevance. The finding does not specifically address experiences with healthcare services, however, describes an underlying cause of behaviour that may be considered a barrier to the uptake of these services. | High confidence  **Explanation:** No/Very minor concerns regarding methodological limitations. No/Very minor concerns regarding coherence. Minor concerns regarding adequacy: one study offered moderately rich data, and two offered thin data. Data were considered adequate (minor concerns) for a descriptive finding. No/Very minor concerns regarding relevance. | Desmond et al. 2013; Griffiths et al. 2012; Mahmoud et al. 2022; |
| 37 | Theme: Financial barriers to healthcare \| Patients and caregivers delayed seeking treatment due to the financial burden associated with healthcare. These financial constraints included not only direct costs of medical services but also transportation to the health facilities. Some families reported having to borrow money to cover healthcare expenses. In some cases, these financial limitations stimulated them to seek alternative medicine before pursuing hospital care. | No/Very minor concerns  **Explanation:** Very minor concerns regarding methodology because two of the three studies did not explicitly state the relationship between researchers and participants (no reflexivity statement), and one study did not clearly justify the research design. These limitations, however, were assessed as not influencing the finding. | No/Very minor concerns  **Explanation:** No concerns regarding coherence | Minor concerns  **Explanation:** Minor concerns regarding adequacy because out of three studies, one study offered rich data, while two other studies together offered thin data. | Minor concerns  **Explanation:** Minor concerns regarding relevance. While all three studies provided data relevant to the research question, one of the studies only focused on caregivers, while the other two offered information about both patients and caregivers. | Moderate confidence  **Explanation:** Very minor concerns regarding methodological limitations: no reflexivity statement in two studies, No concerns regarding coherence, Minor concerns regarding adequacy: one study with rich data, two studies with thin data, and Minor concerns regarding relevance: one study only focused on experiences of caregivers. | Desmond et al. 2013; Griffiths et al. 2012; Omoleke et al. 2018; |
| 38 | Theme: Impact of perceived health service quality on health-seeking behaviour \| Both patients and healthcare workers reported poor organisation of healthcare services with long waiting times, presumptive diagnosis without examination, verbal mistreatment, and lack of follow-up guidance. The perceived suboptimal quality of care prompted patients to avoid hospitals and seek medical advice from alternative service providers. | Moderate concerns  **Explanation:** Moderate concerns regarding methodological limitations. One of the two contributing studies did not provide sufficient data on the relationship between researcher and participants. Considering that the finding reflects participant experiences with and attitudes towards healthcare services, this limitation was judged to potentially influence the finding. Additionally, recall bias could have been introduced, which would be not expected to influence the finding. | No/Very minor concerns  **Explanation:** The finding fully reflects data from one of the two contributing studies. Besides long waiting times, another study also reported higher costs of care in governmental hospitals. However, this aspect was not considered to reflect health services quality, hence, was judged to not impact coherence. | Moderate concerns  **Explanation:** Moderate concerns regarding adequacy. Two studies contributed to the finding: and both offered relatively thin data. Considering the finding is rather descriptive, we concluded that we had moderate concerns about data adequacy. | No/Very minor concerns  **Explanation:** No concerns regarding relevance. The finding reflects end-users' experiences with healthcare services. | Low confidence  **Explanation:** Moderate concerns regarding methodological limitations: no reflexivity statement in one contributing study, which could have potentially influenced the finding. No/Very minor concerns regarding coherence. Moderate concerns regarding adequacy: two studies offered thin data. No/Very minor concerns regarding relevance. | Desmond et al. 2013; Omoleke et al. 2018; |
| 39 | Theme: Lack of early recognition \| Caregivers reported not recognising the early symptoms of meningitis and only seeking help in healthcare facilities when the disease has progressed. Prior experience with meningitis helped raise suspicion earlier. | No/Very minor concerns  **Explanation:** Very minor concerns regarding methodological limitations. Reflexivity statement was not provided in both studies that contributed to the finding. Additionally, in one study, it is unclear whether the research design was appropriate to address the aims. However, these limitations were judged to have minimal effect on the finding. | Minor concerns  **Explanation:** Minor concerns regarding coherence because one of the two studies contributed fully to the finding, while the other study only offered data regarding delayed help-seeking behaviour. | Serious concerns  **Explanation:** Serious concerns regarding adequacy because the overall richness and the quantity of the data are low. Both studies provided thin data. | Minor concerns  **Explanation:** Minor concerns regarding relevance. Even though the finding does not specifically address experiences with healthcare services, it describes a reason why the uptake of these services might be delayed. | Very low confidence  **Explanation:** No/Very minor concerns regarding methodological limitations. Minor concerns regarding coherence: one study supported all aspects of the finding while the other only one part of the finding. Serious concerns regarding adequacy: overall richness and quantity of data are relatively low. Minor concerns regarding relevance: the finding provides information about one barrier to healthcare services uptake. | Adedini et al. 2021; Desmond et al. 2013; |
| 40 | Theme: Disease severity initiates help-seeking behaviour \| The key factor that encouraged caregivers to seek help at conventional healthcare facilities was recognition of disease severity rather than recognition of specific signs and symptoms of meningitis. Indicators of severity, such as social life disruption, severe weakness, loss of appetite, and the inability to work, were among the cited reasons driving individuals to seek help. | No/Very minor concerns  **Explanation:** Very minor concerns regarding methodological limitations. One study did not clarify the time between meningitis onset and the interview, raising concerns regarding potential recall bias. Additionally, the relationship between researcher and participants was unclear in this study. However, it is unlikely that these limitations affected participants' responses. | Moderate concerns  **Explanation:** Moderate concerns regarding coherence. The finding generally supports the underlying data from two studies, reflecting initiation of help-seeking in conventional healthcare facilities following recognition of disease severity. However, one study does not specifically state that disease severity was judged by any specific factor. Additionally, the finding does not cover cases when help-seeking at conventional healthcare facilities was initiated following failed attempts to control the disease with alternative medicine. | Moderate concerns  **Explanation:** Moderate concerns regarding adequacy. Two studies contributed to the finding: one offered moderately rich data, the other one - thin data. However, the descriptive finding relies mostly on the moderately rich data provided in one study, so we concluded we have moderate concerns regarding adequacy. | No/Very minor concerns  **Explanation:** No concerns regarding relevance. The finding reflects acceptability of conventional healthcare services among end-users and factors influencing the uptake of these services, therefore, there were no concerns regarding relevance. | Moderate confidence  **Explanation:** No/Very minor concerns regarding methodological limitations. Moderate concerns regarding coherence: one study reports cases when help-seeking at conventional healthcare facilities was initiated following failed attempts to control the disease with alternative medicine. However, the finding does not cover such cases. Moderate concerns regarding adequacy: one study on which this finding mostly relies offered moderately rich data. One of the contributing studies offered thin data. No/Very minor concerns regarding relevance. | Desmond et al. 2013; Omoleke et al. 2018; |
| SEQUELAE – LMICS | | | | | | | |
| 41 | Theme: Multifaceted impact of meningitis on physical, mental, and social well-being \| Patients emphasised the long-term effects of meningitis on their physical, mental, and social well-being. Older patients and caregivers of children reported a range of complications, including cardiovascular problems, paralysis, hearing and vision impairments, cognitive decline and psychological changes. Meningitis sequelae significantly disrupted social activities and reduced the level of independence. | No/Very minor concerns  **Explanation:** No concerns regarding methodology. Both studies did not explicitly state the relationship between researchers and participants. These limitations, however, were assessed as not having an influence on the finding. | Moderate concerns  **Explanation:** Moderate concerns regarding coherence because the finding lacks several mentioned conditions which were reported by end-users after acute meningitis. | Minor concerns  **Explanation:** Minor concerns regarding adequacy. Two studies contributed to the finding: one offered relatively thin data, and one offered moderately rich data. Considering the finding is rather descriptive, we concluded that there are moderate concerns about data adequacy. | Serious concerns  **Explanation:** Serious concerns regarding relevance. Despite providing data on meningitis sequelae in general, the finding does not address experiences with healthcare services. | Very low confidence  **Explanation:** No/Very minor concerns regarding methodological limitations: no reflexivity in two studies, Moderate concerns regarding coherence: the finding leaves out several reported aspects, Minor concerns regarding adequacy: one study offered relatively thin data and the other one – moderately rich data, and Serious concerns regarding relevance: the finding reflects experiences with meningitis sequelae in general, not with healthcare services. | Griffiths et al. 2012; Mahmoud et al. 2022; |
| 42 | Theme: Experiences with providing care for meningitis sequelae \| Aftercare for family members with meningitis sequelae was associated with some practical and psychological challenges. Carers reported the necessity to balance work comittments and caregiving responsibilities. Those caregivers who continued to work struggled to provide consistent and sufficient care, while others had to abandon their jobs to committ to care for their loved ones. Additionally, a single caregiver reported hiring a specialised perconnel to look after the child with sequelae, which posed a financial strain on the family. Psychologically, caring for older parents was perceived as a rewarding experience by some participants, but as a stressful experience by others. Psychological stress was induced by the feelings of isolation, frustration with taking care of older family members, and fear of economic instability. | No/Very minor concerns  **Explanation:** Very minor concerns regarding methodological limitations. Two contributing studies did not provide enough information on the relationship between researcher and participants. However, this limitation was judged to have a minimal effect on the finding. | No/Very minor concerns  **Explanation:** No concerns regarding coherence. The finding fully reflects the data from the two contributing studies. | Moderate concerns  **Explanation:** Moderate concerns regarding adequacy. Two studies contributed to the finding: one offered moderately rich data, the other - thin data. Moreover, psychological burden of caregiving was reflected only in one study and supported by relatively thin data. Considering the finding is rather descriptive, we concluded that we had moderate concerns about data adequacy. | Serious concerns  **Explanation:** Serious concerns regarding relevance because the finding reflects the general experiences with caregiving for people with meningitis sequelae and not experiences with rehabilitation services. | Low confidence  **Explanation:** No/Very minor concerns regarding methodological limitations. No/Very minor concerns regarding coherence. Moderate concerns regarding adequacy: of the two contributing studies, one offered moderately rich data and one offered thin data. Psychological burden of caregiving was reflected only in one study and supported by relatively thin data. Serious concerns regarding relevance: the finding reflects the general experiences with caregiving and not experiences with rehabilitation services. | Griffiths et al. 2012; Mahmoud et al. 2022; |
| 43 | Theme: Balancing marital and domestic responsibilities and caregiving \| Female caregivers faced challenges in balancing marital and domestic responsibilities while caring for ailing parents. Some women noted that marriage distanced them from their parental homes, complicating their ability for caregiving. In contrast, men were reported to have greater availability to provide care for relatives with meningitis sequelae. | No/Very minor concerns  **Explanation:** Very minor concerns regarding methodological limitations. One study that contributed to the finding did not provide a reflexivity statement. However, these limitations were judged to have minimal effect on the finding. | No/Very minor concerns  **Explanation:** The finding fully reflects data from the single contributing study, hence, there were no concerns regarding coherence. | Serious concerns  **Explanation:** Serious concerns regarding adequacy. Only one study contributed to the finding, offering relatively thin data. | Serious concerns  **Explanation:** Serious concerns regarding relevance. Despite providing data on meningitis sequelae in general, the finding does not address experiences with healthcare services. | Very low confidence  **Explanation:** Very minor concerns regarding methodological limitations: no reflexivity, No concerns regarding coherence, Serious concerns regarding adequacy: one study with thin data, and Serious concerns regarding relevance: the finding reflects experiences with meningitis sequelae in general, not with healthcare services. | Mahmoud et al. 2022; |
| 44 | Theme: Preference for home care over institutionalization \| Most caregivers were sceptic about the benefits of professional aftercare services and feared that institutional care could further deteriorate their loved ones' health. This led to a belief that care should ideally be managed at home within the family. | Moderate concerns  **Explanation:** Moderate concerns regarding methodological limitations. The study that contributed to the finding did not explicitly report the relationship between researcher and participants. Considering the finding addresses views on healthcare services, we concluded this limitation could influence the finding. | No/Very minor concerns  **Explanation:** As the finding is based on one study and reflects all the relevant data, we had no concerns regarding coherence. | Moderate concerns  **Explanation:** Moderate concerns regarding adequacy. One study contributed to the finding, offering relatively thin data. Considering the finding is rather descriptive, we concluded that we had moderate concerns about data adequacy. | No/Very minor concerns  **Explanation:** The finding reflects a barrier to rehabillitation services, therefore, we had no concerns regarding relevance. | Moderate confidence  **Explanation:** Moderate concerns regarding methodological limitations: concerns regarding reflexivity and potential recall bias, which could have affected the finding. No/Very minor concerns regarding coherence. Moderate concerns regarding adequacy: one study contributed to the finding, offering relatively thin data. No/Very minor concerns regarding relevance. | Mahmoud et al. 2022; |
| 45 | Theme: Financial burden as a barrier to aftercare services uptake \| The financial aspects were a barrier to accessing aftercare services for children and adults who have experienced meningitis. Caregivers reported that the high costs associated with transportation, hospital consultations, and medical devices, such as hearing aids, contributed to the discontinuation of aftercare. | No/Very minor concerns  **Explanation:** Very minor concerns regarding methodology because the study did not explicitly state the relationship between researchers and participants. This limitation, however, was assessed as not having an influence on the finding. | No/Very minor concerns  **Explanation:** The finding fully reflects data from the single contributing study, hence, there were no concerns regarding coherence. | Moderate concerns  **Explanation:** Moderate concerns regarding adequacy. Only one study contributed to the finding, offering relatively thin data. Considering the finding is rather descriptive, we concluded that there are moderate concerns about data adequacy, even though only one study contributed to the finding. | No/Very minor concerns  **Explanation:** No concerns regarding relevance because the finding reflects factors influencing the uptake of healthcare services. | Moderate confidence  **Explanation:** Very minor concerns regarding methodological limitations: no reflexivity, No concerns regarding coherence, Moderate concerns regarding adequacy: one study with relatively thin data, and No concerns regarding relevance | Griffiths et al. 2012; |

## Table S3. Themes synthesised from the available evidence

| Theme | Participant type | E/B/F | Country |
| --- | --- | --- | --- |
| Pre-hospitalisation | | | |
| Knowledge and perceptions of meningitis | Comm | E | LMIC |
| Conflict and convergence between biomedical and traditional treatment | P, C | E | LMIC |
| Sociocultural factors influencing help-seeking behaviour | P, C | B | LMIC |
| Initial response regarding preferred treatment | P, C | B | LMIC |
| Lack of awareness and alertness to meningitis symptoms delays timely care | P, C | B | LMIC |
| Financial barriers to healthcare | P, C | B | LMIC |
| Impact of perceived health service quality on help-seeking behaviour | P | B | LMIC |
| Lack of early recognition | C | B | LMIC |
| Recognition of severity as a trigger for help-seeking initiation | C | F | LMIC |
| Need for awareness and comprehensive knowledge about meningitis | P, C | E | HIC |
| Parental emotional reactions during initial stages of meningitis | C | E | HIC |
| Parental intuition and recognition of illness | C | E | HIC |
| Many masks of meningitis clinical presentation | HCW | E | HIC |
| Role of context and parental input in clinical decision-making | HCW | E | HIC |
| Intuitive and evidence-based practice | HCW | E | HIC |
| Sociocultural factors influencing help-seeking behaviour | C | B | HIC |
| Systemic and operational barriers in healthcare organisation | HCW | B | HIC |
| Factors influencing pre-hospital antibiotic treatment initiation | HCW | F | HIC |
| Hospitalisation | | | |
| Perceptions of lumbar puncture outcomes | P, C | E | LMIC |
| Economic impact of medical treatment on families | C | E | LMIC |
| Challenges in diagnosing meningitis | HCW | E | LMIC |
| Miscommunication between healthcare workers and patients/carers | HCW | E | LMIC |
| Fear of complications as a barrier to lumbar puncture uptake | P, C | B | LMIC |
| Reliance on shared decision-making | P, C | B | LMIC |
| Patients' values driving consent to lumbar puncture | P, C | F | LMIC |
| Systemic and operational barriers in healthcare organisation | HCW | B | LMIC |
| Community apprehensions influencing healthcare workers' decision-making | HCW | B | LMIC |
| Consent practices facilitating lumbar puncture delivery | HCW | F | LMIC |
| Need for healthcare workers’ greater awareness/alertness and rapid decision-making | P, C | E | HIC |
| Importance of appropriate communication and information from healthcare workers | C | E | HIC |
| Parental emotional turmoil during hospitalisation | C | E | HIC |
| Coping strategies and emotional support during hospitalisation | C | E | HIC |
| Sequelae | | | |
| Multifaceted impact of meningitis on physical, mental, and social well-being | P | E | LMIC |
| Experiences with providing care for meningitis sequelae | C | E | LMIC |
| Balancing marital and domestic responsibilities and caregiving | C | B | LMIC |
| Preference for home care over institutionalisation | C | B | LMIC |
| Financial burden as a barrier to aftercare services uptake | C | B | LMIC |
| Multifaceted impact of meningitis on physical, mental, and social well-being | P, C | E | HIC |
| Long-term psychological impact of hospitalisation | P, C | E | HIC |
| Parental concerns about potential consequences of meningitis | C | E | HIC |
| Need for care continuity, education and support | C | E | HIC |
| Perceptions of quality of aftercare for meningitis sequelae | C | E | HIC |
| Lack of appreciation for less apparent sequelae of meningitis | C | B | HIC |
| Systemic and organisational barriers to aftercare | C | B | HIC |
| Third parties as facilitators of meningitis sequelae aftercare | C | F | HIC |

P, patients; C, caregivers; Comm, Community; HCW, healthcare workers; E, experiences; B, barriers; F, facilitators; LMIC, low-/middle-income country; HIC, high-income country.
